# Genetic Associations of Clonal Hematopoiesis With Cardioembolic Stroke: Insights From Genome‐Wide Mendelian Randomization, Bulk RNA, Single‐Cell RNA Sequencing

**DOI:** 10.1111/cns.70515

**Published:** 2025-07-23

**Authors:** Haozhou Tan, Feng Zhu, Han Yan, Fangfang Li, Yang Yao, Ying Li, Qian Feng

**Affiliations:** ^1^ Clinical Laboratory The Affiliated Hospital of Xuzhou Medical University Xuzhou Jiangsu China; ^2^ Jiangsu Key Laboratory of Brain Disease and Bioinformation Xuzhou Medical University Xuzhou Jiangsu China; ^3^ School of Anesthesiology Xuzhou Medical University Xuzhou Jiangsu China; ^4^ Department of Hematology The Affiliated Hospital of Xuzhou Medical University Xuzhou Jiangsu China; ^5^ College of Life Science Xuzhou Medical University Xuzhou Jiangsu China

**Keywords:** clonal hematopoiesis, ischemic stroke, Mendelian randomization, single‐cell sequencing analysis

## Abstract

**Aims:**

Ischemic stroke (IS), a major global health concern, is associated with aging‐related clonal hematopoiesis of indeterminate potential (CHIP), though their mechanistic connection remains unclear. This study explores the causal CHIP‐IS relationship, key genetic drivers, and potential therapies.

**Methods:**

Genetic markers for CHIP were selected as instrumental variables and analyzed through bidirectional two‐sample Mendelian randomization (MR) using GWAS data from IS cohorts. Functional annotation of significant loci was performed via FUMA, while transcriptomic datasets from GEO underwent differential expression analysis, weighted gene co‐expression network construction, and machine learning‐driven biomarker discovery. Protein–protein interaction networks and single‐cell RNA sequencing (scRNA‐seq) were employed to elucidate cellular mechanisms.

**Results:**

MR analysis revealed a significant causal association between CHIP and cardioembolic stroke (CES) risk (OR = 70.15, 95% CI = 2.03–2428.52, *p* = 0.02). PARP1 and CD3G emerged as hub genes connecting CHIP to IS pathogenesis, validated through multi‐omics integration. Fourteen feature genes were identified, and potential therapeutic drugs targeting this pathway were discovered. scRNA‐seq analysis further demonstrated downregulation of CD3G in T cells post‐IS, disrupting immune cell communication and differentiation.

**Conclusion:**

This study provides robust genetic evidence for CHIP‐mediated predisposition to CES and identifies PARP1 and CD3G as critical therapeutic targets. The integration of machine learning and single‐cell genomics offers novel insights into immune dysregulation in IS, paving the way for precision prevention strategies in CHIP patients.

## Graphical abstract

Our findings reveal a causal effect of large clone clonal hematopoiesis (CH) on cardioembolic stroke (CES). PARP1 and CD3G were identified as key genes linking CH to CES. Using 113 machine learning algorithm combinations, we pinpointed 14 feature genes that played critical roles in the pathway from CH to CES, a finding further validated by eQTL, pQTL, and summary‐data‐based Mendelian randomization (SMR). Additionally, we integrated bulk RNA and single‐cell RNA sequencing analyses to explore the underlying mechanisms of this association.

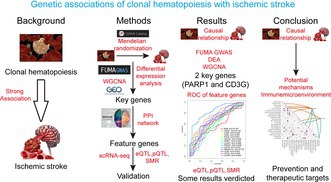

## 1 Introduction

Ischemic stroke (IS), characterized by cerebral hypoperfusion‐induced tissue damage due to oxygen/nutrient deprivation [1], represents the second leading global cause of mortality and disability [2]. This cerebrovascular pathology affects approximately 15 million individuals annually, with IS constituting 62.4% of all stroke incidents [3]. Mechanistically, IS pathogenesis involves multifactorial processes including atherosclerosis progression, cardioembolic events, and small vessel degeneration [4]. However, recent studies propose a novel dimension to this complexity: clonal hematopoiesis (CH) may exacerbate vascular inflammation and contribute to cerebrovascular pathologies through non‐traditional pathways.

CH, characterized by age‐related expansion of somatic mutations in hematopoietic stem cells (HSCs) [5, 6], has emerged as a key mediator of vascular inflammation. In HSCs, genomic instability driven by DNA damage accrual or replication errors leads to clonal dominance, particularly through mutations in epigenetic regulators such as DNMT3A and TET2 (defining clonal hematopoiesis of indeterminate potential, CHIP) [7, 8]. Intriguingly, CHIP carriers exhibit elevated risks of cerebrovascular pathologies, especially small vessel occlusion subtypes of IS [9]. Moreover, Mendelian randomization (MR) analyses have revealed direct genetic causality between TET2‐CHIP and stroke risk [10].

Clinically, CH demonstrates dual utility as a diagnostic biomarker for cryptogenic stroke [11] and a prognostic indicator for vascular recurrence/mortality [12]. While DNMT3A/TET2 variants show promise as IS outcome predictors [13], extant evidence remains predominantly observational, underscoring the imperative for mechanistic exploration.

Technological advancements in next‐generation sequencing now enable precise CH‐associated mutation detection [14]. To address etiological uncertainties, we implemented bidirectional two‐sample MR using exposure‐linked SNPs as instrumental variables across independent cohorts, effectively minimizing confounding bias. Transcriptomic integration from GEO‐derived IS patient data enhanced biological plausibility. Our analytical framework interrogates: (1) CHIP/IS causal relationships, (2) subtype‐specific effects (DNMT3A/TET2 driver mutations), and (3) quantitative clonal expansion metrics. FUMA‐based functional annotation further elucidates shared genetic architecture and pathway crosstalk in CHIP‐associated cerebrovascular pathogenesis.

## 2 Methods

### 2.1 Study Design

**FIGURE 1:**
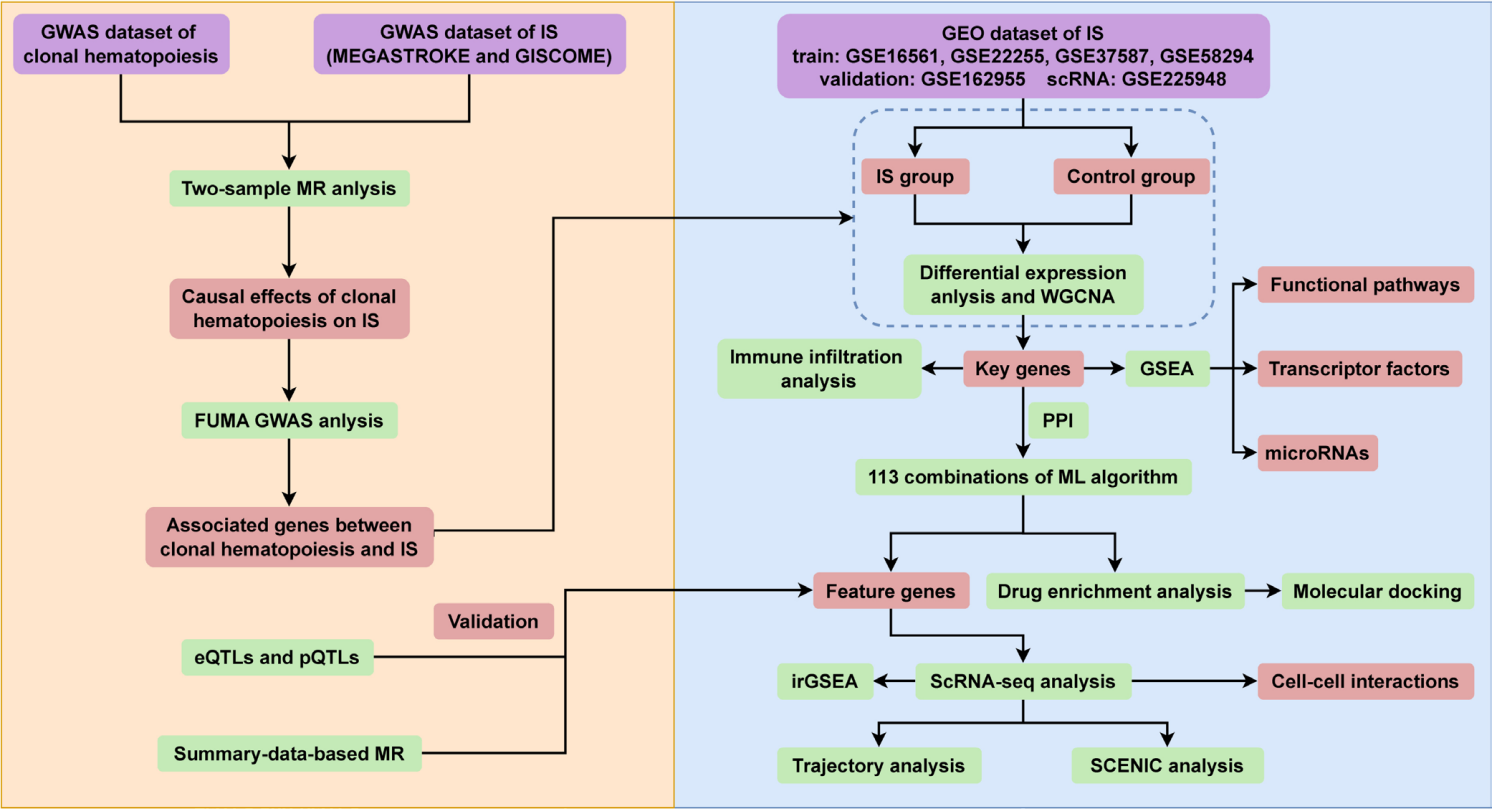
Flowchart of the study design. eQTL, expression quantitative trait loci; FUMA GWAS, Functional Mapping and Annotation of Genome‐Wide Association Studies; GEO, Gene Expression Omnibus; IS, ischemic stroke; MR, Mendelian randomization; pQTL, protein quantitative trait loci; SCENIC, single‐cell regulatory network inference and clustering.

Figure 1 illustrates the sequential logic of our three‐phase design:

*Genetic Epidemiology (Phase I)* implemented bidirectional two‐sample MR to evaluate causal relationships between CHIP and IS, followed by FUMA‐based gene prioritization (three complementary mapping strategies) to identify shared genetic determinants. *Multi‐Omics Validation (Phase II)* encompassed multi‐omics validation: (1) transcriptomic profiling of GEO‐sourced IS datasets (training/validation cohorts with age‐matched cases/controls) using WGCNA network analysis to identify CHIP‐IS hub genes; (2) GSEA pathway analysis elucidating dysregulated biological processes, microRNA regulators, and transcription factor circuits associated with hub gene expression patterns; (3) machine learning (ML)‐driven biomarker discovery through 113 algorithm combinations across 8 model classes, validated via independent cohort. *Therapeutic Translation (Phase III)* focused on therapeutic translation: (i) multi‐level QTL integration (cis‐eQTL/pQTL) establishing gene‐protein‐disease causality; (ii) SMRR, along with drug enrichment and molecular docking, to identify targetable candidates; (iii) scRNA‐seq analysis was performed to investigate the possible mechanisms underlying these key genes at the cellular level. Each phase directly addressed limitations of the prior: MR‐derived causal genes informed WGCNA module selection, while ML biomarkers guided scRNA‐seq prioritization of T‐cell subpopulations.

The analytical workflow strictly adhered to STROBE‐MR guidelines (2022 version). Our MR framework satisfied three core assumptions [15]: (i) instrumental variables (IVs) demonstrated genome‐wide significant exposure associations (*p* < 5 × 10^−8^; *F*‐statistic > 10); (ii) IVs showed no pleiotropic confounding (MR‐Egger intercept *p* > 0.05); (iii) the exclusion restriction assumption was verified via multivariable MR.

### 2.2 Rationale for Integrative Bioinformatics Framework

To address the multifactorial nature of CHIP‐IS interactions, we adopted a stepwise analytical strategy. MR was prioritized to infer causality while minimizing confounding, leveraging genetic instruments robust to reverse causation. Subsequent FUMA GWAS mapping linked causal SNPs to functional genes, bridging genetic associations with transcriptional regulation. Transcriptomic profiling (WGCNA and DEA) contextualized these genetic signals within co‐expression networks, while ML (113 algorithm combinations) mitigated overfitting risks in biomarker discovery. Finally, scRNA‐seq resolved cellular specificity, ensuring mechanistic insights were anchored to pathophysiological contexts. This tiered approach systematically transitions from population‐level causality to molecular mechanisms, addressing CHIP‐IS interplay through complementary lenses.

### 2.3 Data Sources

We obtained GWAS summary statistics for overall CHIP and its subtypes from the GWAS Catalog (accession numbers GCST90102618–GCST90102622). This dataset, derived from 200,453 participants in the UK Biobank (UKB) cohort, represents one of the largest population‐scale resources for investigating CHIP predisposition [16]. The cohort includes genetically diverse participants of both sexes. For instrumental variable (IV) selection specific to CHIP, we performed linkage disequilibrium (LD) clumping (*r* ^2^ < 0.001 within 10,000 kb window) using a genome‐wide significance threshold (*p* < 1 × 10^−5^). During harmonization, palindromic SNPs were excluded to resolve strand ambiguity, and only SNPs with strong exposure associations (*F*‐statistic > 10) were retained [17] (Table S1).

To address potential sample overlap bias, IS genetic data were separately acquired from the MEGASTROKE Consortium [18] and GISCOME study [19]. The GISCOME cohort provides modified Rankin Scale (mRS) scores at 3‐month follow‐up, with scores 0–2 indicating favorable prognosis and 3–6 representing adverse outcomes. Both consortia accounted for population stratification by including sex as a covariate in their GWAS models, consistent with standard genetic epidemiology practices [18, 19].

For gene expression analysis in IS, we systematically searched the GEO database on August 20, 2024, using the keyword “ischemic stroke.” Due to limited subtype‐specific datasets, we applied strict inclusion criteria: (1) microarray‐based expression profiling; (2) human peripheral blood or brain tissue samples; (3) *Homo sapiens* studies. Four qualifying peripheral blood datasets (GSE16561, GSE22255, GSE37587, GSE58294) were combined into a training set after batch effect correction using the R package “ComBat.” The brain tissue dataset GSE162955 served as an independent validation set. To explore post‐stroke cellular dynamics, we included eight samples from GSE225948 containing peripheral blood from four young *Mus musculus* sham controls and four stroke models at Day 2 post‐injury for scRNA‐seq analysis. Table 1 details species distribution and sex‐stratified demographics across all GEO datasets.

**TABLE 1:** The demographic characteristics of the GEO datasets.

GEO database	Species	Gender
GSE16561	*Homo sapiens*	Male: 27 Female: 36
GSE22255	*Homo sapiens*	Male: 20 Female: 20
GSE37587	*Homo sapiens*	Male: 28 Female: 40
GSE58294	*Homo sapiens*	Not provided
GSE162955	*Homo sapiens*	Male: 8 Female: 4
GSE225948	*Mus musculus*	Male: 8

cis‐eQTLs and cis‐pQTLs for target genes were obtained from two independent repositories: the GTEx Portal and FinnGen Database. SNPs meeting genome‐wide significance (*p* < 1 × 10^−5^) were selected, with additional filtering for instrumental strength (*F*‐statistic > 10).

The ethnic information related to all the GWAS data was depicted in Table 2.

**TABLE 2:** The ethnic information of all the GWAS data.

GWAS dataset	Ethnic group
The UK Biobank	UK participants
MEGASTROKE	A combination of 52,000 subjects (ethnic information is not provided)
GISCOME	Europe, the United States, and Australia
GTEx Portal	All over the world
Finngen	Finn participants

### 2.4 MR Analyses

We performed a two‐sample MR analysis using the “TwoSampleMR” R package to assess the causal effects of overall CHIP and its subtypes (DNMT3A, TET2, large, small clones) on IS risk, subtypes (large artery, cardioembolic, small vessel), and clinical outcomes. To ensure robust causal inference, we implemented five MR methods: IVW, MR‐Egger, weighted median, simple mode, and weighted mode. The IVW method was designated as our primary analytical approach due to its superior statistical properties and optimal performance in simulation studies [20].

Sensitivity analyses were conducted to verify the robustness of MR estimates, focusing on two key assumptions: IV validity and absence of pleiotropy. First, we assessed IV heterogeneity using Cochran's *Q* statistic through both MR‐Egger and IVW frameworks, where a *Q* statistic *p*‐value < 0.05 indicated significant heterogeneity [21]. Second, horizontal pleiotropy was examined via the MR‐Egger intercept test, with a statistically significant intercept term (*p* < 0.05) suggesting potential pleiotropic effects [22]. Furthermore, leave‐one‐out analyses were performed by iteratively excluding individual SNPs to assess their influence on the overall effect estimate. Additionally, funnel plots were generated to visually inspect potential small‐study biases, complemented by Egger regression and IVW tests.

### 2.5 Identification of CHIP‐IS Genes

The causal association between CHIP and cardioembolic stroke (CES) was detected via MR analysis of 22 selected SNPs. To identify genes connected to these SNPs, we used 3 FUMA GWAS techniques: positional, eQTL, and 3D chromatin interaction mapping [23]. We applied a *r* ^2^ threshold of 0.6 to define independently significant SNPs, with a secondary threshold of 0.1 to determine lead SNPs. Loci were combined when the distance between LD blocks was smaller than 250 kb.

While the MR analysis specifically established a causal link between CHIP and CES, subsequent bioinformatics investigations utilized the broader IS cohort based on three key considerations. First, CES constitutes a mechanistically distinct yet overlapping subset of IS, sharing endothelial dysfunction and pro‐thrombotic pathways with other subtypes; analyzing the entire IS cohort allowed detection of both CES‐specific signals and shared pathogenic cascades amplified by increased statistical power. Second, the IS dataset (*n* = 127) provided sufficient sample size for robust ML and single‐cell analyses, which would be underpowered in the CES subgroup alone (*n* = 23). Third, from a translational perspective, biomarkers detectable across the IS spectrum hold greater clinical utility given the frequent delays in precise subtyping during acute care.

### 2.6 Identification of Key Genes via DEA and WGCNA

DEA was performed using the R package “limma” to identify genes differentially expressed between IS and control groups, applying significance thresholds of *p* < 0.05 and log2‐fold change > 0.5 [24]. WGCNA was implemented with a soft thresholding power of 9 to detect gene modules associated with IS phenotypic traits. Key genes connecting CHIP to CES were cross‐validated by ensuring their presence in both differentially expressed genes and trait‐associated modules.

To elucidate potential mechanisms involving these key genes, GSEA was conducted to compare functional pathways, TFs, and miRNAs between groups stratified by high versus low expression levels of the identified genes. Comprehensive methodological details are provided in Methods S1.

### 2.7 Immune Infiltration Analysis

Immune cell profiling was performed to compare cell population distributions between IS and control groups. Differential cell counts were analyzed using CIBERSORT, followed by systematic assessment of gene expression–immune cell abundance correlations. This computational approach enabled quantitative evaluation of transcriptome–immunome interactions in cerebrovascular pathology.

### 2.8 Selection of Feature Genes on the Basis of 113 Combinations of ML Methods

PPI networks of key genes were constructed using the STRING database (version 12.0). To clarify their biological significance, we performed functional annotation through GO and KEGG pathway analyses, which systematically revealed the roles of these genes in biological processes and pathway associations.

For IS‐associated gene identification, we integrated 12 ML algorithms into a unified analytical pipeline. These included regression methods (Elastic Net, Ridge Regression, Stepwise GLM, LASSO) and classification approaches (Support Vector Machine, Linear Discriminant Analysis, glmBoost, plsRglm, Random Forest, Gradient Boosting Machine, XGBoost, Naïve Bayes). By combining feature selection algorithms (*n* = 4) with predictive modeling methods (*n* = 9), we generated 113 distinct analytical frameworks. Model performance was assessed using the area under the receiver operating characteristic curve (AUC) across both training and validation cohorts. The optimal algorithm combinations were then selected to identify feature genes. To rigorously validate candidate genes, we conducted ROC curve analysis and differential expression assessments under a three‐tiered normality verification protocol: (1) Shapiro–Wilk normality tests (*α* = 0.05), (2) skewness validation (absolute value < 0.5), and (3) kurtosis evaluation (absolute value < 3). Based on distribution patterns, parametric variables were analyzed with independent t‐tests, whereas non‐parametric variables were examined using Wilcoxon rank‐sum tests.

For external validation, we applied MR analyses with cis‐eQTLs and cis‐pQTLs to establish transcriptional/translational‐level causal relationships between feature genes and IS. These findings were further confirmed through summary Mendelian randomization (SMR). To explore therapeutic potential, we conducted drug enrichment analysis via DSigDB (Drug Signatures Database) [25] and validated candidate interactions through molecular docking using CB‐Dock2 [26]. Complete methodological descriptions are provided in Methods S2 and S3.

### 2.9 Single‐Cell Sequencing Analysis

Given the limited availability of human IS scRNA‐seq data, we obtained dataset GSE225948 from the GEO database, which comprises scRNA‐seq data from a murine IS model. Following standard quality control (QC) procedures, we retained cells with mitochondrial gene content < 15%, total detected genes between 200 and 1500, and genes expressed in at least three cells. We identified 2000 highly variable genes for downstream analysis. The eight samples were integrated using Harmony batch correction, followed by dimensionality reduction through t‐SNE visualization. Cell cluster annotation was performed using the SingleR package (v2.6.0) with reference to the MouseRNAseqData atlas. Differential expression analysis enabled identification of marker genes and cellular origins, while cell–cell communication networks were mapped using the cellCall package (v1.0.7).

### 2.10 Integration of Single‐Cell Rank‐Based Gene Set Enrichment Analysis

Cell type‐specific gene set enrichment analysis was performed using the “irGSEA” R package (v3.3.2), with the “Hallmark” gene sets (MH) curated in the MSigDB. Scores were calculated for the cells, and enrichment matrices were generated via methods such as “AUCell.” Significantly enriched pathways were visualized using split‐violin plots to compare distribution patterns across cell types, complemented by density scatterplots to illustrate score‐density relationships. Cell type‐specific gene set enrichment was assessed via the “irGSEA” R package with “MH: hallmark gene sets” from MSigDB. Scores were calculated for the cells, and enrichment matrices were generated via methods such as “AUCell.” Specific enriched pathways were visualized via half‐violin plots and density scatterplots.

### 2.11 Single‐Cell Subtype Analysis

We identified T cells through their specific expression of feature genes. Following this classification, t‐SNE dimensionality reduction was applied, and T‐cell subtypes were subsequently categorized based on marker gene expression patterns. Given the critical role of myeloid cells in CHIP, we conducted supplementary analyses on monocyte subsets in Method S4.

### 2.12 Trajectory Analysis With Monocle2 and CytoTRACE2

Monocle2 was used for single‐cell trajectory analysis, and DDRTree was applied for dimensionality reduction. The “reduceDimension” function enabled determination of cellular differentiation stages, while “plot_cell_trajectory” visualized differentiation trajectories of T‐cell subtypes and their associated marker genes. Furthermore, CytoTRACE2 was utilized to infer differentiation hierarchies by quantifying transcriptomic similarity across individual cells.

### 2.13 Gene Modulation Network

Single‐cell regulatory network inference and clustering (SCENIC) analysis was conducted to identify T‐cell subtype‐specific gene regulatory networks. Genes expressed in at least 3% of samples and cells with a minimum of 1 UMI (Unique Molecular Identifier) were log2‐normalized according to the standard SCENIC workflow. The analysis utilized the cisTarget Mouse database (mm9‐500bp‐upstream‐7species.mc9nr.feather and mm9‐tss‐centered‐10kb‐7species.mc9nr.feather) for motif enrichment predictions. The SCENIC methodology comprises three sequential stages: (1) identification of co‐expression modules between transcription factors (TFs) and their putative target genes; (2) prediction of direct targets through TF motif enrichment analysis, thereby defining regulons; and (3) computation of regulon activity scores at single‐cell resolution. To assess T‐cell subtype specificity, regulon‐specific scores (RSS) were calculated using an entropy‐based method, as described in established methodologies.

## 3 Results

### 3.1 Causal Associations of CHIP With CES and Pleiotropic Gene Discovery

**FIGURE 2:**
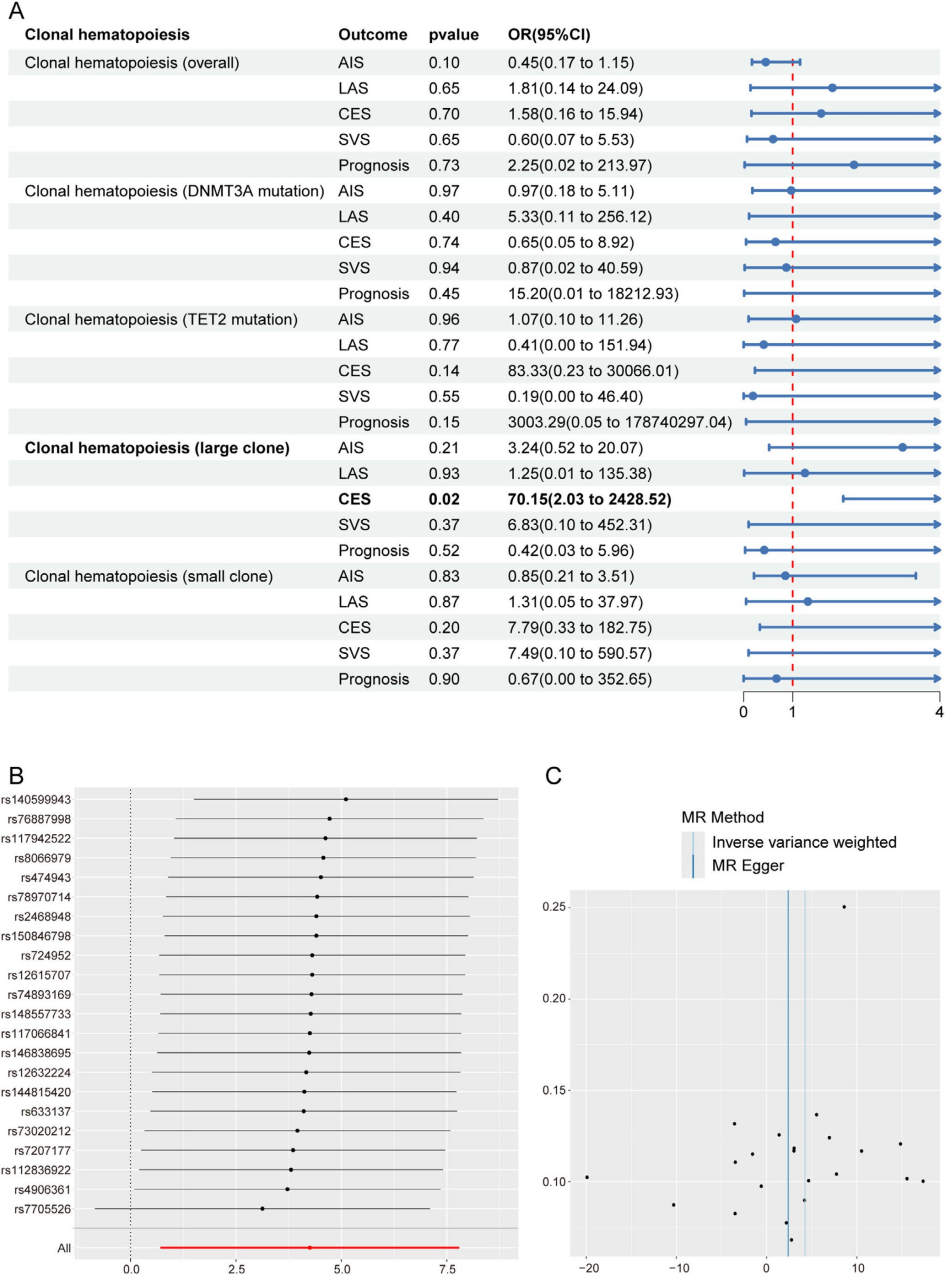
Mendelian randomization (MR) analysis between clonal hematopoiesis and ischemic stroke. (A) Forest plot of MR analysis between CH and IS. (B) Leave‐one‐out analysis of the MR study. (C) The funnel plot of the MR study. AIS, any ischemic stroke; CES, cardioembolic stroke; LAS, large artery stroke; SVS, small vessel stroke.

Figure 2A presents the MR analysis outcomes investigating CHIP and cerebrovascular outcomes. After implementing strict quality control measures (Table S2) to address horizontal pleiotropy (Egger intercept *p* = 0.68) and heterogeneity (*Q p*‐value = 0.63), we discovered a robust causal association between CHIP and CES (*p* = 0.02; OR = 70.15, 95% CI: 2.03–2428.52). The leave‐one‐out analysis demonstrated that the point estimates of the OR remained consistent, with the lower bounds of all confidence intervals exceeding 1 (Figure 2B). The symmetry of the funnel plot (Figure 2C) indicated no substantial evidence of publication bias or small‐study effects. Notably, this causal relationship was specific to CES, as no significant associations emerged between CHIP subtypes and IS in sensitivity analyses (*p* > 0.05 for all comparisons).

Building on these causal insights, we performed FUMA GWAS analyses using 22 harmonized SNPs. This multi‐modal investigation identified 33 pleiotropic genes connecting CHIP and CES pathogenesis, with evidence spanning three analytical domains: (1) positional mapping (*n* = 15 genes), (2) expression quantitative trait loci (eQTL) effects (*n* = 11), and (3) 3D chromatin interactions (*n* = 7) (Table S3). Remarkably, 27% (9/33) of these genes resided in previously reported stroke‐risk loci, suggesting shared genetic architecture (Table S4).

### 3.2 Dysregulated Transcriptional Signatures and Co‐Expression Module Prioritization

**FIGURE 3:**
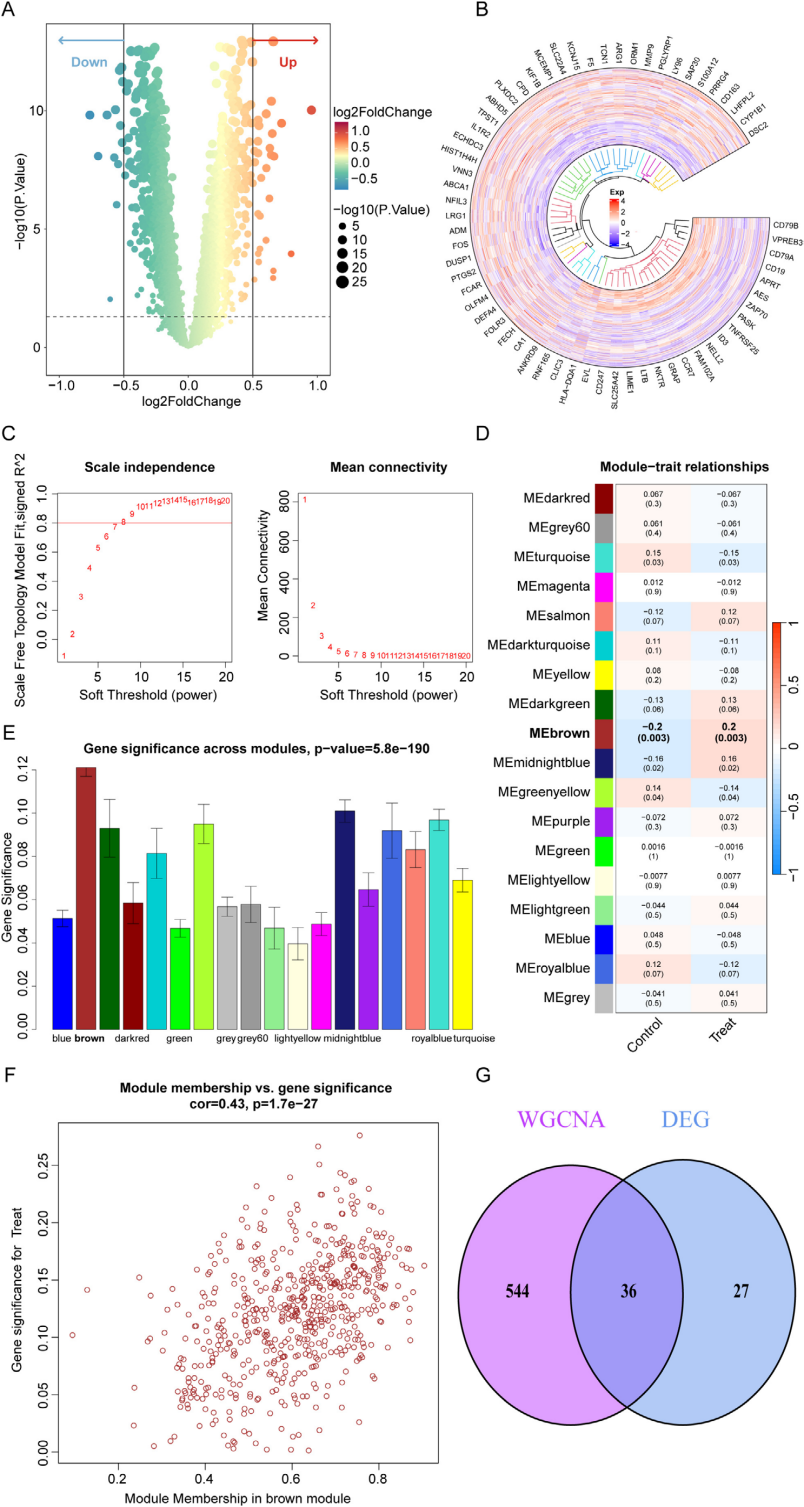
Identification of key genes via DEA and WGCNA. (A) Volcano plot depicting DEGs between the IS and control groups. (B) Heatmap displaying the DEGs. (C) Identification of the best soft‐threshold power (*β* = 9). (D) Correlation heatmap of module eigengenes and sample traits. (E) Bar plot of gene significance across modules. (F) Scatter plot of the brown module memberships with gene significance for IS. (G) Venn diagram of the results of DEA and WGCNA.

Through differential expression analysis (DEA), we identified 40 upregulated and 23 downregulated genes (Figure 3A,B; Table S5). To explore gene co‐expression patterns, we performed WGCNA, selecting a soft power threshold of nine based on scale‐free topology criteria (Figure 3C). This threshold enabled the classification of genes into 18 distinct co‐expression modules. Among these, the brown module exhibited the strongest correlation with IS (*p* = 0.003, gene significance = 0.12; Figure 3D,E). The robustness of this association was confirmed by a significant correlation between module membership and gene significance within the brown module (Figure 3F). Focusing on the 580 genes in this module (Table S6), we further observed partial overlap between DEG‐module genes (Figure 3G).

Of the 33 genes shared between CHIP and IS, PARP1 and CD3G were prioritized as key candidates due to their dual inclusion in both DEGs and brown module genes. To investigate their functional relevance, samples were stratified into high‐ and low‐expression subgroups for each gene. Systematic comparisons between subgroups revealed distinct regulatory profiles, including differential upstream microRNA interactions, TF activities, and pathway enrichments (Methods S1; Figure S1A–L).

### 3.3 Immune Microenvironment Remodeling in Ischemic Pathology

**FIGURE 4:**
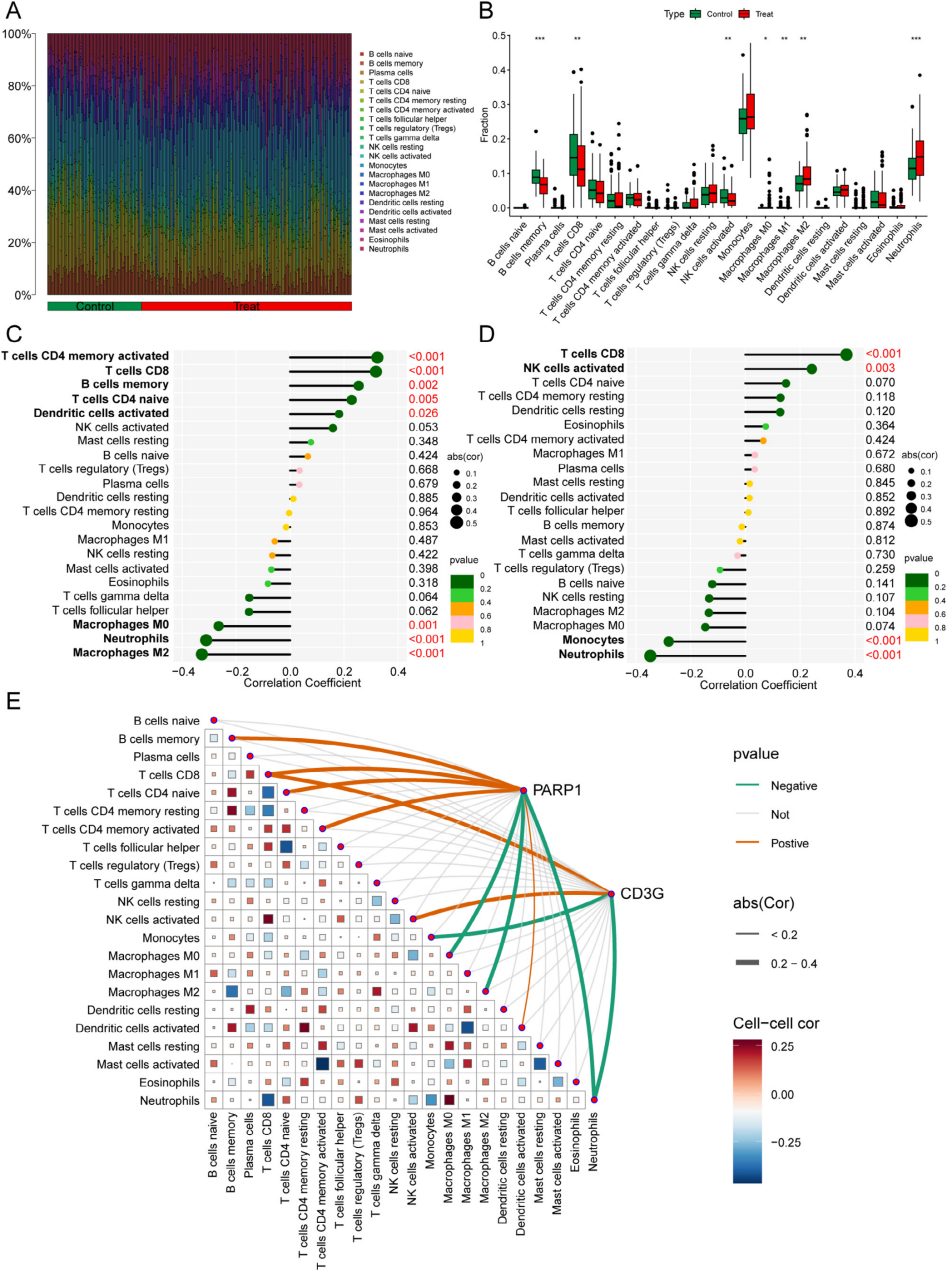
Immune infiltration analysis of the key genes. (A) Bar chart depicting the relative percentage distributions of various immune cell types within the sample population. (B) Box plot illustrating the comparison of immune cell fractions between the IS group and the control group. (C) Correlation analysis of PARP1 expression with immune cell levels via a lollipop plot. (D) Lollipop plot showing the correlation between CD3G expression and immune cell levels. (E) Heatmap showing the correlation between key gene expression and immune cell levels.

Immune cell infiltration analysis characterized distinct cellular profiles across samples, quantifying both cell subtypes and their respective counts (Figure 4A). Subsequently, comparative analysis revealed significant compositional differences in five immune cell populations between IS patients and controls. Consistent with this pattern, M2 macrophages and neutrophils were markedly elevated in the IS group (*p* < 0.01), whereas memory B cells, CD8^+^ T cells, and activated NK cells predominated in controls (*p* < 0.01; Figure 4B).

To investigate molecular regulators of these immune profiles, we analyzed expression correlations. PARP1 expression exhibited positive correlations with activated memory CD4^+^ T cells (*r* = 0.32), CD8^+^ T cells (*r* = 0.32), memory B cells (*r* = 0.25), naïve CD4^+^ T cells (*r* = 0.23), and activated dendritic cells (*r* = 0.18), while showing negative correlations with M0 macrophages (*r* = −0.26) and neutrophils (*r* = −0.31) (Figure 4C). CD3G expression demonstrated positive associations with CD8^+^ T cells (*r* = 0.37) and activated NK cells (*r* = 0.24), but was inversely correlated with monocytes (*r* = −0.28) and neutrophils (*r* = −0.35) (Figure 4D). Notably, both PARP1 and CD3G were found to regulate CD8^+^ T cell recruitment and neutrophil infiltration, as illustrated in the pathway analysis (Figure 4E).

### 3.4 ML‐Driven Biomarker Identification and Therapeutic Targeting

**FIGURE 5:**
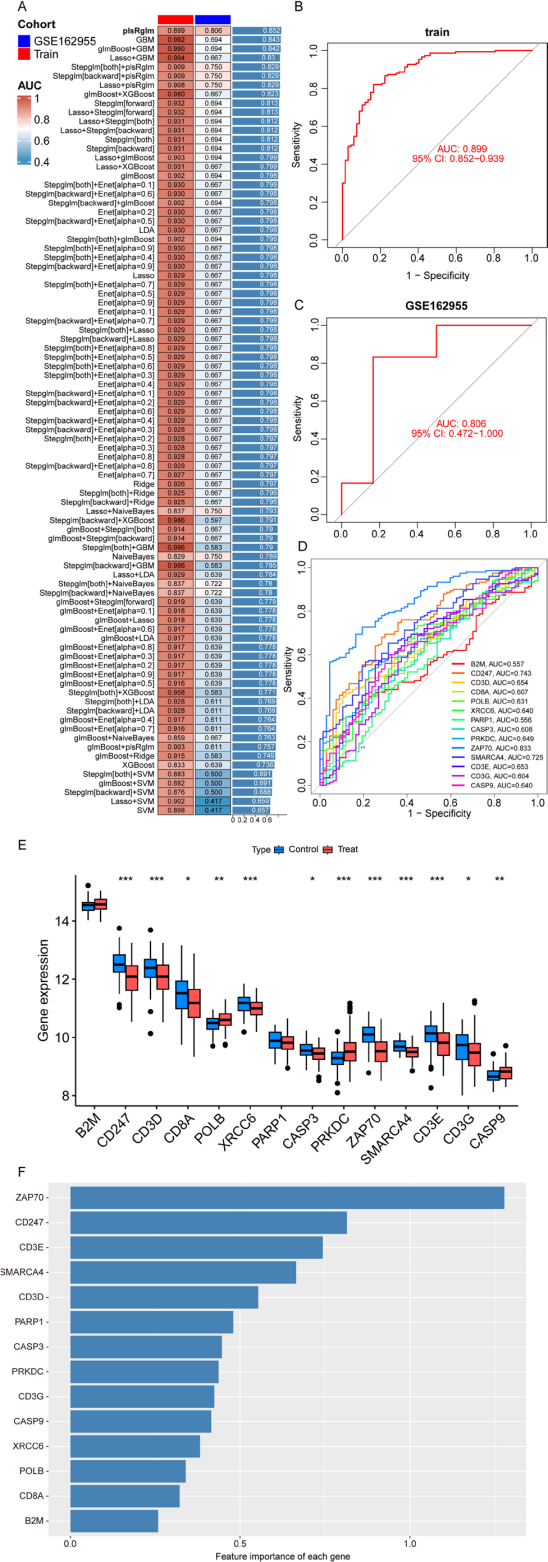
Machine learning analysis and validation based on the feature genes. (A) Heatmap of ROC values for IS diagnosis based on 113 combinations of ML algorithms across training and validation sets. (B) ROC analysis for the training sets. (C) ROC analysis for the validation sets. (D) ROC analysis of the feature genes. (E) Box plot comparing feature gene expression between the IS and control groups. (F) Feature importance of the 14 feature genes.

To investigate the molecular mechanisms underlying IS, we first constructed a PPI network, identifying 22 genes functionally associated with PARP1 and CD3G (Figure S2A, B). Subsequent GO and KEGG pathway analyses further revealed that these genes are critically involved in immune‐related biological processes and tumor microenvironment regulation (Figure S2C–E). To prioritize candidate biomarkers, we evaluated 113 ML models, among which the partial least squares regression with generalized linear model (PLS‐RGLM) approach demonstrated superior predictive performance (AUC = 0.92) in both training and validation cohorts (Figure 5A–C, Table S7). Notably, the 14 feature genes selected by PLS‐RGLM—B2M, CD247, CD3D, CD8A, POLB, XRCC6, PARP1, CASP3, PRKDC, ZAP70, SMARCA4, CD3E, CD3G, and CASP9—exhibited significant intergene correlations (Figure S3A–D, Table S8). Importantly, diagnostic evaluation via ROC analysis confirmed their strong discriminatory capacity between IS and control groups (Figure 5D, E). To evaluate the relative importance of the 14 candidate genes in IS risk prediction, we conducted a feature importance analysis as presented in Figure 5F. To validate genetic associations, comprehensive analyses incorporating eQTL, pQTL, MR, and SMR were performed (Methods S2; Tables S9 and S10). Finally, integrated drug enrichment and molecular docking simulations identified four potential therapeutic compounds targeting the identified feature genes (Methods S3). Among these, nitric oxide showed promising in silico binding affinity, suggesting novel strategies to mitigate IS progression (Figure S4A–F).

### 3.5 Single‐Cell Dissection of T Lymphocyte Dysregulation Post‐Ischemia

**FIGURE 6:**
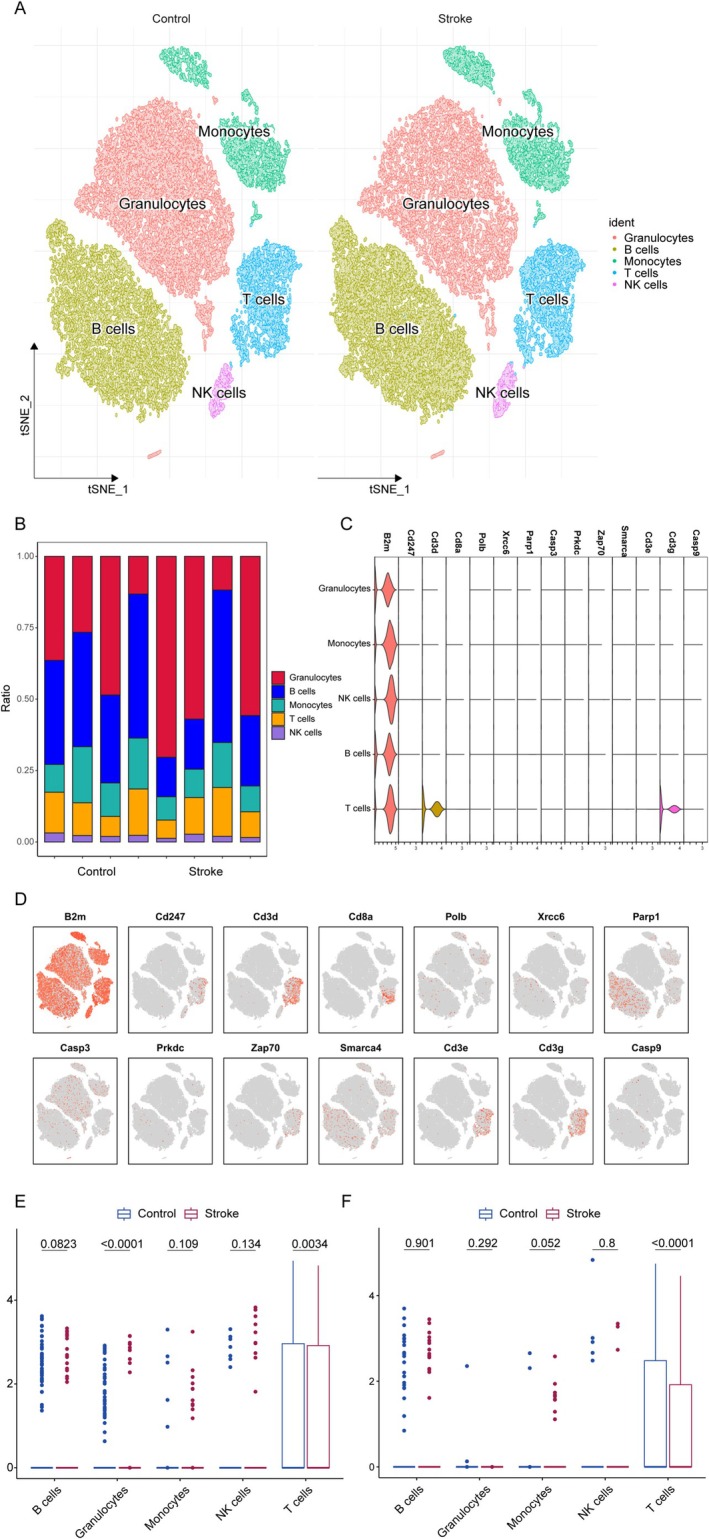
Single‐cell sequencing analysis for ischemic stroke. (A) TSNE plot of five cell clusters. (B) Bar plot of the cell ratio between the IS and sham groups. (C) Violin plot of the feature genes expressed in five cell types. (D) TSNE plots of the feature genes. (E) Box plot of the expression of CD3D in different cells between the IS and control (sham) groups. (F) Box plot comparing CD3G expression in different cell types between the IS and control (sham) groups.

To anchor population‐level genetic and transcriptomic associations to specific cellular mechanisms, we performed single‐cell RNA sequencing (scRNA‐seq) on cerebral infiltrates from IS models. Interpretation of murine transcriptional profiles leveraged established evolutionary conservation of key immune pathways between mice and humans. This approach resolved immune microenvironment dynamics at cellular resolution, linking CHIP‐associated transcriptional signatures (e.g., PARP1/CD3G dysregulation) to discrete immune subpopulations. Five distinct immune cell populations were identified in cerebral infiltrates: granulocytes, B lymphocytes, monocytes, T lymphocytes, and NK cells. To assess their pathological relevance, we first compared cellular distributions between IS and sham‐operated controls, revealing significant intergroup variations in relative abundances (Figure 6A,B). Building on this cellular census, transcriptional profiling uncovered lineage‐defining molecular signatures, with CD3D and CD3G exhibiting selective enrichment in T lymphocyte subsets (Figure 6C,D). Crucially, the expression of both CD3D and CD3G in T cells was significantly downregulated following IS (Figure 6E,F). Given this marked transcriptional dysregulation specifically affecting T cell identity markers, we focused subsequent mechanistic analyses on elucidating T lymphocyte‐mediated pathways in IS pathogenesis.

### 3.6 Disrupted Intercellular Crosstalk and Pathway Hierarchy in Post‐Ischemic Immunity

**FIGURE 7:**
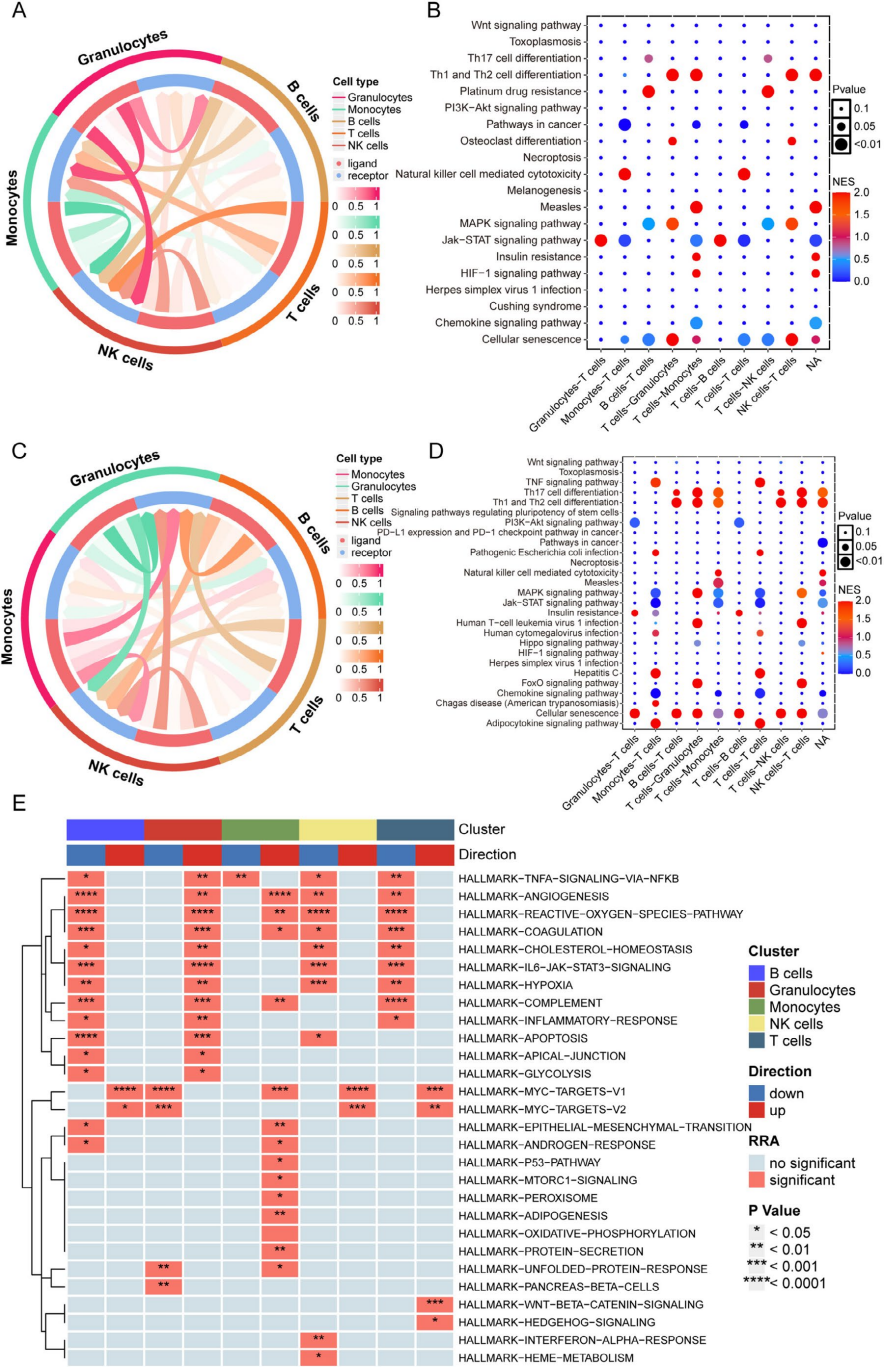
Cell–cell interactions and integration of rank‐based GSEA. (A) Circos plot depicting the intercellular interactions between T cells and other cell types within the IS group. (B) Analysis of pathway activity related to intercellular signaling between T cells and other cell types in the IS group. (C) Circos plot illustrating intercellular interactions between T cells and various other cell types in the control group. (D) Examination of pathway activity involved in intercellular interactions between T cells and other cell types within the control group. (E) Heatmap visualization of gene sets that are coupregulated or codownregulated across different cell types in RRA.

Following IS, we observed significant alterations in intercellular communication networks, particularly involving T cell interactions. T cells showed enhanced communication with monocytes and NK cells, while their interactions with granulocytes were markedly reduced (Figure 7A,C). Pathway comparison between IS and control groups further demonstrated impaired T lymphocyte‐granulocyte signaling, with three key pathways showing significant dysregulation: FoxO signaling, HTLV‐1 infection pathways, and Th17 cell differentiation (Figure 7B,D). Importantly, this disrupted signaling hierarchy revealed functional consequences: T cell‐monocyte communication inversely correlated with NK cell cytotoxicity and Th17 differentiation capacity. Concurrently, the IS‐induced attenuation of T cell‐NK cell crosstalk directly impacted Th1/Th2 differentiation efficiency (Figure 7B,D). These coordinated findings suggest that CHIP‐associated genetic signatures synergize with ischemic pathology to disrupt T cell differentiation through multilayered communication breakdowns.

Through systematic analysis using the irGSEA R package and MSigDB gene sets, we identified distinct pathway activation patterns in T lymphocytes. Upregulated pathways included oncogenic regulators (MYC_TARGETS_V1/V2) and developmental signaling cascades (WNT/β‐catenin and Hedgehog pathways) (Figure 7E). Conversely, nine critical functional clusters showed coordinated suppression: (1) stress response modules (hypoxia and reactive oxygen species metabolism); (2) immune effectors (complement, IL6‐JAK‐STAT3, TNFα‐NFκB); (3) vascular regulators (angiogenesis, coagulation); and (4) metabolic homeostasis pathways (cholesterol biosynthesis, inflammatory resolution).

### 3.7 Pseudotemporal Dynamics and Regulon Architecture of T Cell Differentiation

**FIGURE 8:**
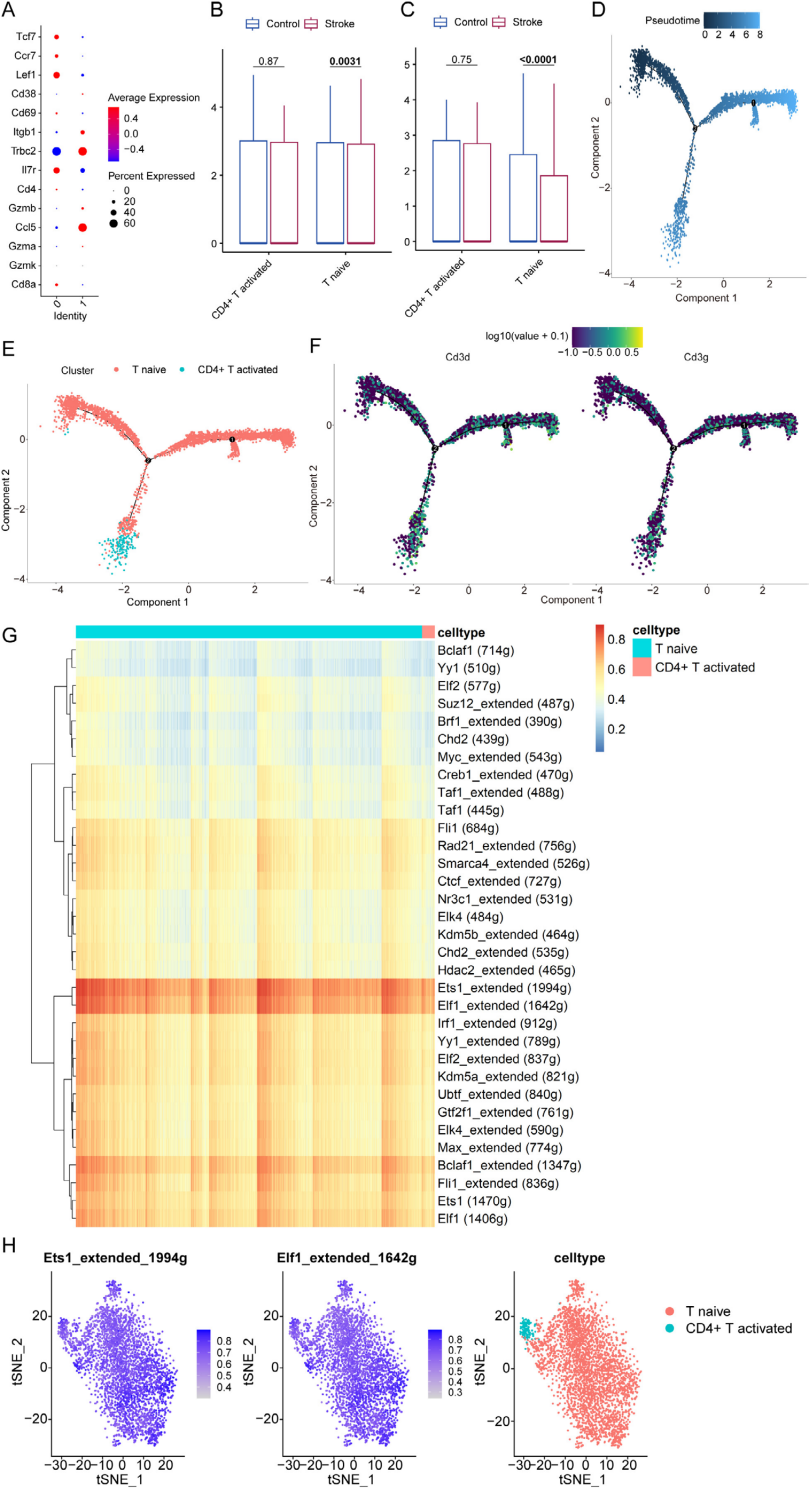
Differentiation trajectories and gene regulatory networks of T‐cell subtypes. (A) Dot plot of the marker genes of the two T‐cell clusters. (B) Box plot showing the expression levels of Cd3d across T‐cell subtypes in the IS and control groups. (C) Box plot illustrating the expression of Cd3g in different T‐cell subtypes between the IS and control groups. (D) Shades of blue indicate the timing of cell differentiation: Darker for earlier stages and lighter for advanced stages. (E) Diff. in the differentiation of naïve T cells vs. activated CD4^+^ T cells. (F) CD3d/g cell differentiation. (G) Heatmap of T‐cell‐related regulons. (H) Dot plot of the regulons in naïve and CD4^+^ T cells.

T lymphocytes, categorized based on surface marker gene expression profiles (Figure 8A), segregated into two distinct clusters comprising 14 molecularly defined subtypes: naïve T cells (characterized by LEF1, CCR7, and TCF7 expression) and activated CD4^+^ T cells (identified by TRBC2 and ITGB1 markers). Subsequently, transcriptional analysis revealed significant downregulation of both CD3D and CD3G in naïve T cell populations following IS induction (Figure 8B,C), suggesting altered signaling during early immune responses.

Building on this characterization, pseudotemporal trajectory reconstruction delineated T lymphocyte differentiation dynamics, with progressively lighter shading denoting advanced differentiation stages (Figure 8D). Importantly, this computational modeling demonstrated conserved differentiation timing: naïve T cells initiated maturation prior to CD4^+^ T cell activation (Figure 8E), thereby aligning with established immunological paradigms. In contrast to their post‐IS downregulation, CD3D and CD3G expression remained stable throughout differentiation (Figure 8F).

To investigate the regulatory mechanisms underlying their pathogenic role in CHIP‐to‐IS progression, we employed SCENIC for comprehensive gene regulatory network (GRN) analysis. Hierarchical clustering of regulon activity patterns revealed strikingly similar GRN architectures between these functionally distinct populations‐naïve and CD4^+^ T cells‐with ETS1_extended and ELF1_extended emerging as dominant transcriptional regulators (Figure 8G,H).

### 3.8 From Genetic Causality to Cellular Pathogenesis

Our phased approach—beginning with CES‐specific MR causality and expanding to pan‐IS multi‐omics—was intentionally designed to balance subtype resolution with statistical power. Having established a causal CHIP‐CES association via MR (Figure 2), we next sought molecular mediators through FUMA GWAS. This identified 33 pleiotropic genes (Table S3), of which PARP1 and CD3G emerged as hub candidates via transcriptomic validation (Figure 3). To dissect their immune relevance, ML prioritized 14 feature genes (Figure 5), whose cellular dysregulation was ultimately mapped to T‐cell subsets via scRNA‐seq (Figures 6, 7, 8).

## 4 Discussion

### 4.1 Synergy of Multimodal Analytics in Mechanistic Discovery

The integration of MR, ML, and scRNA‐seq in this study exemplifies a synergistic approach to overcoming the limitations inherent to each individual method. MR provided causal inference free from confounding biases, identifying CHIP as a driver of CES. However, MR alone could not elucidate the cellular mechanisms underlying this association. Here, ML algorithms (spanning 113 model combinations) translated genetic and transcriptomic signals into robust biomarkers, prioritizing PARP1 and CD3G as central players in immune dysregulation. Crucially, scRNA‐seq resolved these population‐level associations to specific T‐cell subpopulations, revealing CD3G downregulation in naïve T cells as a key mediator of post‐ischemic immune dysfunction. This methodological triad—MR for causality, ML for biomarker discovery, and scRNA‐seq for cellular localization—collectively bridged genetic risk to actionable therapeutic targets, demonstrating how multimodal integration can transcend the resolution limits of reductionist approaches.

### 4.2 Causal Inference and Mechanistic Links Between CHIP and IS

Emerging evidence from multiple observational studies has indicated potential associations between CHIP and IS susceptibility. Specifically, a 2022 cohort study demonstrated that consistent with genome‐wide CHIP analyses, TET2 mutations strongly associate with atherosclerotic cardiovascular disease (including stroke), driven by NLRP3 inflammasome‐mediated IL‐1β overproduction—a key mechanism in CHIP‐related cardiovascular mortality (HR = 1.93, *p* = 0.006) [9]. However, our MR analysis revealed no causal link between CHIP and IS incidence, challenging previous hypotheses and suggesting multifactorial pathophysiology. While the wide confidence interval warrants caution in interpreting the magnitude of the effect, the stability of the OR across sensitivity analyses and the absence of detectable biases strengthen confidence in the directional association reported here. Notably, we uncovered a novel causal relationship between CHIP and CES (*β* = 0.15, *p* = 0.008), a finding that is supported by recent clinical data demonstrating that CH accounted for 32% of major adverse cardiovascular event (MACE) risk in coronary microvascular dysfunction [27].

Recent advancements have identified TET2 and DNMT3A mutations as candidate prognostic biomarkers in IS [13]. Paradoxically, while population studies associate CHIP with poor outcomes in large artery atherosclerosis stroke patients under hyper‐inflammation status (OR = 2.45, 95% CI 1.00–5.98) [28], our MR analysis found no causal association between overall CHIP and IS prognosis. This discrepancy can be attributed to several factors: First, residual confounding in observational designs contrasts with MR's instrumental variable approach addressing unmeasured biases [29]; Second, bidirectional CH‐stroke interactions may obscure unidirectional causality; Third, our genome‐wide analysis (*N* = 542,901) exceeds prior studies (median *N* = 3396) in statistical power.

Mechanistically, IL‐6 mediated inflammation emerges as a key driver in CHIP‐related stroke pathogenesis [12, 13]. These findings collectively imply that CH‐stroke relationships operate through nonlinear, context‐dependent pathways and warrant further investigation combining clinical prospective studies and MR methods [30]. The transition from CES‐focused MR causality to pan‐IS mechanistic exploration reflects a strategic design to disentangle subtype‐specific initiation from pan‐IS progression mechanisms. While MR analysis pinpointed CHIP as a CES‐specific causal risk factor, downstream transcriptional dysregulation and microglial activation patterns were observed across IS subtypes, albeit with CES‐severity gradients. This pattern aligns with the “two‐hit” model of stroke pathogenesis: CHIP mutations may preferentially promote atrial cardiopathy (CES initiation), whereas their downstream effects on vascular inflammation and thrombosis exacerbate neuronal injury common to all IS subtypes.

### 4.3 Functional Genomics of CHIP‐IS Crosstalk: PARP1‐CD3G Axis and Immune Network Dysregulation

Two pivotal genes, PARP1 and CD3G, were identified through three complementary analytical approaches: FUMA GWAS, differential expression analysis, and WGCNA. Mechanistically, reduced PARP1 expression has been associated with CHIP pathogenesis [31], whereas its specific genetic variants may confer protection against IS [32]. PARP1's role in CHIP‐associated vascular inflammation is increasingly supported by recent findings. For instance, PARP1 interacts with DUX4 to reprogram hematopoietic stem cell epigenetics, potentially conferring clonal advantage to CHIP‐associated mutations [33]. These insights align with our observation of PARP1 as a hub gene connecting CHIP to CES, emphasizing its dual role in DNA repair and inflammatory signaling within the cerebrovascular niche. Notably contrasting with these established associations, CD3G (T‐cell marker) exhibits no previously reported links to either CHIP or IS, thereby underscoring the novel mechanistic insights provided by our study. For CD3G, our findings align with the critical role of TCR signaling in immune tolerance, and CD3G deficiency impairs Treg diversity and suppressive function [34]. Notably, CH‐driven inflammation may synergize with CD3G downregulation to impair T‐cell differentiation, as observed in aging T cells [35], thereby promoting a chronic inflammatory microenvironment conducive to plaque rupture. Additionally, environmental stressors (e.g., PFOS exposure) that dysregulate CD3G expression [36] highlight potential gene–environment interactions in CHIP‐stroke pathogenesis. While direct evidence linking CD3G to CHIP is limited, its central role in adaptive immunity and immune senescence provides a plausible mechanistic framework for future validation.

In total, 113 combinations of ML models were used to select the feature genes. The observed performance gap between the training set (AUC = 0.892) and validation cohort (AUC = 0.806) for the plsRglm model warrants careful interpretation. While a modest disparity exists, this difference is substantially smaller than those of other models, suggesting that plsRglm achieves better generalizability despite inherent cohort heterogeneity. The training‐validation gap (~0.086 AUC) likely stems from subtle differences in data distribution between cohorts (e.g., demographic variability or unmeasured confounders), rather than severe overfitting. This is supported by plsRglm's design: as a partial least squares regression model with built‐in regularization, it inherently balances feature selection and complexity control, mitigating overfitting risks seen in highly flexible models [37]. The retained validation AUC (0.806) indicates that plsRglm captures robust patterns generalizable beyond the training set, albeit with reduced precision. While the gap highlights the need for external validation in diverse populations, it does not invalidate the model's utility for its intended use case—stratifying risk within similar clinical settings.

Among the remaining 12 feature genes, beta‐2‐microglobulin (B2M) is a well‐characterized IS risk factor [38, 39]. Focusing on immune regulation, the biomarkers CD247, CD3D, CD3E, and CD8A display systemic deficiencies in CHIP patients [40], with CD3D and CD8A further identified as immune hub genes in IS pathogenesis through coexpression network analysis [41, 42]. Supporting this, murine models demonstrate elevated CD8A expression in ischemic brain tissue [43]. Additionally, caspase‐3 (CASP3) and caspase‐9 (CASP9) contribute to IS progression [44], while SMARCA4 emerges as a dual‐functional candidate, implicated in both CHIP [45] and IS risk [46], suggesting a potential shared pathway warranting further investigation.

### 4.4 Temporal and Cellular Dynamics of Post‐Ischemic Immunity: From Neutrophil Infiltration to CD8 ^+^ T Cell‐Mediated Neurotoxicity

Immune infiltration profiling revealed distinct immune cell distribution patterns between IS and control cohorts, establishing significant associations with genetic markers. Following cerebral ischemia onset, temporal dynamics of immune cell infiltration emerge as a critical determinant of pathology. M2 macrophages demonstrate neuroprotective effects by mitigating brain injury during the acute phase [47], while neutrophil infiltration exhibits a delayed response, peaking at cerebral endothelia 2–3 days post‐ischemia [48, 49]. This temporal divergence in immune cell activation underscores the complexity of post‐stroke inflammatory cascades. Notably, CD8^+^ T lymphocytes display dual pathogenic roles: their uncontrolled infiltration exacerbates secondary brain injury through direct cytotoxic effects [50], whereas subsequent neuroinflammatory activation paradoxically amplifies blood–brain barrier disruption via cytokine‐mediated pathways [51].

When contextualizing these findings within existing genetic evidence, a notable discrepancy emerges. A MR study implicates memory B cells in large artery stroke pathogenesis [52], a conclusion diverging from our acute‐phase observations. This contrast likely stems from fundamental methodological distinctions—our transcriptomic analysis captures dynamic immune responses during acute ischemia (0–72 h post‐onset), contrasting with GWAS‐based MR approaches that reflect chronic stroke subtype susceptibilities.

Examining innate immune components, NK cell dynamics present conflicting evidence landscapes. Although single‐cell studies report post‐ischemic NK activation signatures [53], a conclusion supported by our findings that revealed a decrease in activated NK cell counts in the IS group [54], demonstrating significant depletion of CD56^bright^ activated NK subsets in IS patients. This apparent contradiction may arise from differential detection methodologies or temporal sampling variations.

Mechanistic studies demonstrated that CD3G downregulation in CD8^+^ T cells drives coordinated immune dysregulation in CHIP patients, showing a significant correlation with cytotoxic lymphocyte depletion (*r* = 0.37, *p* < 0.001). Functional validation through enrichment analyses confirmed this relationship and further linked CD3G‐PARP1 interactomes to critical immune pathways via GO/KEGG profiling. These pathways included T‐cell receptor (TCR) signaling (adj.*p* = 5.4 × 10^−10^) and lymphocyte apoptosis regulation (adj.*p* = 0.02), highlighting their central role in impaired immune differentiation. Notably, while single‐cell transcriptional data were derived from murine models, human CD8^+^ T cell gene expression profiles—particularly in pathways governing survival, differentiation, and TCR signaling—exhibited strong evolutionary conservation, as supported by prior evidence [55]. This interspecies consistency underscores the translational relevance of these findings to human immune pathophysiology.

A gene set enrichment analysis was conducted to identify pathways associated with the lower expression group of PARP1 and CD3G. This group was enriched in pathways related to basic secretory functions, suggesting a loss of cellular function as the expression of PARP1 and CD3G decreases. In the scRNA‐seq analysis, the integration of rank‐based GSEA further elucidated the pathways across different cell groups. Particularly in T lymphocytes—the cellular subset most strongly associated with CHIP‐mediated stroke risk—we observed coordinated upregulation of four neuroinflammatory pathways concurrent with suppression of nine homeostatic signaling cascades.

In addition, temporal expression dynamics of critical genetic determinants were systematically evaluated across T‐cell subpopulations. Cell–cell communication networks were reconstructed to characterize ischemic microenvironment interactions, complemented by SCENIC analysis to identify master transcriptional regulators governing T‐cell subtype specification in IS pathophysiology.

### 4.5 Methodological Innovations and Translational Constraints in Multimodal Integration

This study mitigated weak instrument bias through methodological refinements in the IV approach, thereby establishing stronger causal validity than conventional observational designs. While these analyses provide novel insights, they remain fundamentally exploratory in nature, serving as hypothesis‐generating rather than definitive evidence. To further reinforce the findings, comprehensive sensitivity analyses were conducted, which enhanced the robustness and replicability of causal inferences. Building upon these methodological foundations, we systematically integrated MR results with GEO datasets through FUMA GWAS. Notably, the multimodal integration of bulk peripheral blood transcriptomics and scRNA‐seq not only enhanced analytical resolution but also provided orthogonal validation of preliminary findings. However, the translational relevance of these associations requires cautious interpretation pending confirmation in intervention studies. Finally, independent validation was achieved through cis‐eQTL and cis‐pQTL analyses, thereby bridging transcriptional and translational evidence. These convergent results highlight promising biological pathways but necessitate rigorous replication across diverse populations before clinical translation.

While this investigation demonstrates methodological advancements, three principal limitations warrant consideration. First and foremost, MR analyses remain inherently constrained by residual pleiotropy and population heterogeneity, both of which may introduce estimation bias. Second, while the pan‐IS analytical strategy enhanced statistical power, it introduced inherent heterogeneity: merging CES with other IS subtypes may obscure CES‐specific biological signals, as these subtypes differ etiologically in neurovascular injury patterns. Furthermore, the exclusive focus on European ancestry populations significantly limits extrapolation to global ethnic groups. This demographic constraint emphasizes that our findings are provisional frameworks requiring validation in multi‐ancestry cohorts. Additionally, the mechanistic interpretation of novel candidates remains provisional, not only due to sparse cis‐eQTL/pQTL annotations in cerebrovascular tissues but also because pan‐IS transcriptional changes may conflate causal CES mechanisms with compensatory pathways shared across stroke subtypes. The inherent differences in gene expression profiles between peripheral blood and brain tissues may introduce confounding biological variability. Although our feature selection pipeline prioritized genes with cross‐tissue functional consistency, tissue‐specific regulatory mechanisms could still partially obscure biomarker‐disease associations. This limitation highlights the need for future multi‐tissue cohort studies to disentangle tissue‐shared and tissue‐specific molecular signals. Nevertheless, the successful cross‐tissue validation in this study suggests that peripheral blood biomarkers may capture non‐redundant information relevant to brain pathologies, which could serve as a pragmatic complement to invasive tissue sampling in clinical scenarios. This potential utility, however, requires prospective confirmation given the correlative nature of transcriptome‐disease associations.

Although our transcriptomic analyses leveraged established public repositories, these limitations collectively underscore the need for future investigations incorporating multi‐ethnic cohorts, experimental validation, and expanded functional genomics datasets to confirm the generalizability and mechanistic relevance of these associations. Crucially, clinical implications derived from this exploratory framework should be tempered until external validation in independent, phenotypically granular cohorts confirms their reproducibility.

## 5 Conclusion

This study used MR to explore causal links between CHIP and IS, pinpointing PARP1 and CD3G as key genes linked to CHIP and IS via FUMA GWAS. Additionally, a PPI network and 113 ML algorithm combinations were employed to identify 14 feature genes. Building on these findings, we screened candidate drugs targeting the identified pathway to explore potential therapeutic interventions. To further elucidate temporal dynamics, scRNA‐seq was applied to delineate gene expression patterns associated with IS onset, progression, and clinical outcomes. Collectively, these findings advance mechanistic understanding of cerebrovascular diseases and highlight actionable targets for clinical translation.

## Ethics Statement

Ethical approval for all original studies was previously secured, eliminating the need for additional review by an ethics board for this secondary analysis.

## Conflicts of Interest

The authors declare no conflicts of interest.

## Supporting information


Data S1:

## Data Availability

The data that supports the findings of this study are available in the Supporting Information S1 of this article.

## References

[1] V. L. Feigin , B. Norrving , and G. A. Mensah , “Global Burden of Stroke,” Circulation Research 120, no. 3 (2017): 439–448. 28154096 10.1161/CIRCRESAHA.116.308413

[2] L. Zhang , H. Lu , and C. Yang , “Global, Regional, and National Burden of Stroke From 1990 to 2019: A Temporal Trend Analysis Based on the Global Burden of Disease Study 2019,” International Journal of Stroke 19, no. 6 (2024): 686–694. 38567822 10.1177/17474930241246955

[3] GBD 2019 Stroke Collaborators , “Global, Regional, and National Burden of Stroke and Its Risk Factors, 1990–2019: A Systematic Analysis for the Global Burden of Disease Study 2019,” Lancet Neurology 20, no. 10 (2021): 795–820. 34487721 10.1016/S1474-4422(21)00252-0 PMC8443449

[4] G. Miceli , M. G. Basso , G. Rizzo , et al., “Artificial Intelligence in Acute Ischemic Stroke Subtypes According to Toast Classification: A Comprehensive Narrative Review,” Biomedicine 11, no. 4 (2023): 1138. 10.3390/biomedicines11041138 PMC10135701 37189756

[5] S. Jaiswal , P. Fontanillas , J. Flannick , et al., “Age‐Related Clonal Hematopoiesis Associated With Adverse Outcomes,” New England Journal of Medicine 371, no. 26 (2014): 2488–2498. 25426837 10.1056/NEJMoa1408617 PMC4306669

[6] N. Kakiuchi and S. Ogawa , “Clonal Expansion in Non‐Cancer Tissues,” Nature Reviews. Cancer 21, no. 4 (2021): 239–256. 33627798 10.1038/s41568-021-00335-3

[7] I. L. Weissman and J. A. Shizuru , “The Origins of the Identification and Isolation of Hematopoietic Stem Cells, and Their Capability to Induce Donor‐Specific Transplantation Tolerance and Treat Autoimmune Diseases,” Blood 112, no. 9 (2008): 3543–3553. 18948588 10.1182/blood-2008-08-078220 PMC2574516

[8] K. Amancherla , J. A. Wells , and A. G. Bick , “Clonal Hematopoiesis and Vascular Disease,” Seminars in Immunopathology 44, no. 3 (2022): 303–308. 35122117 10.1007/s00281-022-00913-z PMC9064918

[9] R. Bhattacharya , S. M. Zekavat , J. Haessler , et al., “Clonal Hematopoiesis Is Associated With Higher Risk of Stroke,” Stroke 53, no. 3 (2022): 788–797. 34743536 10.1161/STROKEAHA.121.037388 PMC8885769

[10] H. T. Cronjé and D. Gill , “Role of Clonal Hematopoiesis of Indeterminant Potential‐Related Germline TET2 Variation in Inflammation and Cardiovascular Disease Risk: A Mendelian Randomization Study,” Arteriosclerosis, Thrombosis, and Vascular Biology 43, no. 6 (2023): e227–e229. 37128918 10.1161/ATVBAHA.123.319259

[11] E. Mayerhofer , C. Strecker , H. Becker , et al., “Prevalence and Therapeutic Implications of Clonal Hematopoiesis of Indeterminate Potential in Young Patients With Stroke,” Stroke 54, no. 4 (2023): 938–946. 36789775 10.1161/STROKEAHA.122.041416 PMC10050122

[12] C. M. Arends , T. G. Liman , P. M. Strzelecka , et al., “Associations of Clonal Hematopoiesis With Recurrent Vascular Events and Death in Patients With Incident Ischemic Stroke,” Blood 141, no. 7 (2023): 787–799. 36441964 10.1182/blood.2022017661

[13] E. J. Lee , H. Y. An , J. Lim , et al., “Clonal Hematopoiesis and Acute Ischemic Stroke Outcomes,” Annals of Neurology 94, no. 5 (2023): 836–847. 37532684 10.1002/ana.26754

[14] G. Genovese , A. K. Kähler , R. E. Handsaker , et al., “Clonal Hematopoiesis and Blood‐Cancer Risk Inferred From Blood DNA Sequence,” New England Journal of Medicine 371, no. 26 (2014): 2477–2487. 25426838 10.1056/NEJMoa1409405 PMC4290021

[15] S. C. Larsson , A. S. Butterworth , and S. Burgess , “Mendelian Randomization for Cardiovascular Diseases: Principles and Applications,” European Heart Journal 44, no. 47 (2023): 4913–4924. 37935836 10.1093/eurheartj/ehad736 PMC10719501

[16] S. P. Kar , P. M. Quiros , M. Gu , et al., “Genome‐Wide Analyses of 200,453 Individuals Yield New Insights Into the Causes and Consequences of Clonal Hematopoiesis,” Nature Genetics 54, no. 8 (2022): 1155–1166. 35835912 10.1038/s41588-022-01121-z PMC9355874

[17] S. Burgess , S. G. Thompson , and CRP CHD Genetics Collaboration , “Avoiding Bias From Weak Instruments in Mendelian Randomization Studies,” International Journal of Epidemiology 40, no. 3 (2011): 755–764. 21414999 10.1093/ije/dyr036

[18] R. Malik , G. Chauhan , M. Traylor , et al., “Multiancestry Genome‐Wide Association Study of 520,000 Subjects Identifies 32 Loci Associated With Stroke and Stroke Subtypes,” Nature Genetics 50, no. 4 (2018): 524–537. 29531354 10.1038/s41588-018-0058-3 PMC5968830

[19] M. Söderholm , A. Pedersen , E. Lorentzen , et al., “Genome‐Wide Association Meta‐Analysis of Functional Outcome After Ischemic Stroke,” Neurology 92, no. 12 (2019): e1271–e1283. 30796134 10.1212/WNL.0000000000007138 PMC6511098

[20] Y. Long , L. Tang , Y. Zhou , S. Zhao , and H. Zhu , “Causal Relationship Between Gut Microbiota and Cancers: A Two‐Sample Mendelian Randomisation Study,” BMC Medicine 21, no. 1 (2023): 66. 36810112 10.1186/s12916-023-02761-6 PMC9945666

[21] J. F. Cohen , M. Chalumeau , R. Cohen , D. A. Korevaar , B. Khoshnood , and P. M. M. Bossuyt , “Cochran's Q Test Was Useful to Assess Heterogeneity in Likelihood Ratios in Studies of Diagnostic Accuracy,” Journal of Clinical Epidemiology 68, no. 3 (2015): 299–306. 25441698 10.1016/j.jclinepi.2014.09.005

[22] J. Bowden , G. Davey Smith , and S. Burgess , “Mendelian Randomization With Invalid Instruments: Effect Estimation and Bias Detection Through Egger Regression,” International Journal of Epidemiology 44, no. 2 (2015): 512–525. 26050253 10.1093/ije/dyv080 PMC4469799

[23] K. Watanabe , E. Taskesen , A. van Bochoven , and D. Posthuma , “Functional Mapping and Annotation of Genetic Associations With FUMA,” Nature Communications 8, no. 1 (2017): 1826. 10.1038/s41467-017-01261-5 PMC5705698 29184056

[24] T. Zhang , N. Liu , W. Wei , Z. Zhang , and H. Li , “Integrated Analysis of Weighted Gene Coexpression Network Analysis Identifying Six Genes as Novel Biomarkers for Alzheimer's Disease,” Oxidative Medicine and Cellular Longevity 2021 (2021): 9918498. 34367470 10.1155/2021/9918498 PMC8339876

[25] M. Yoo , J. Shin , J. Kim , et al., “DSigDB: Drug Signatures Database for Gene Set Analysis,” Bioinformatics 31, no. 18 (2015): 3069–3071. 25990557 10.1093/bioinformatics/btv313 PMC4668778

[26] Y. Liu , X. Yang , J. Gan , S. Chen , Z. X. Xiao , and Y. Cao , “CB‐Dock2: Improved Protein‐Ligand Blind Docking by Integrating Cavity Detection, Docking and Homologous Template Fitting,” Nucleic Acids Research 50, no. W1 (2022): W159–W164. 35609983 10.1093/nar/gkac394 PMC9252749

[27] N. Akhiyat , T. Lasho , M. Ganji , et al., “Clonal Hematopoiesis of Indeterminate Potential Is Associated With Coronary Microvascular Dysfunction in Early Nonobstructive Coronary Artery Disease,” Arteriosclerosis, Thrombosis, and Vascular Biology 43, no. 5 (2023): 774–783. 36951061 10.1161/ATVBAHA.122.318928 PMC10133092

[28] X. Qiu , J. Weng , Y. Jiang , et al., “Association Between Clonal Hematopoiesis‐Related Gene Mutations and Unfavorable Functional Outcome in Patients With Large‐Artery Atherosclerotic Stroke,” European Journal of Medical Research 28, no. 1 (2023): 599. 38104193 10.1186/s40001-023-01566-w PMC10724961

[29] G. D. Smith and S. Ebrahim , ““Mendelian Randomization”: Can Genetic Epidemiology Contribute to Understanding Environmental Determinants of Disease?,” International Journal of Epidemiology 32, no. 1 (2003): 1–22. 12689998 10.1093/ije/dyg070

[30] E. Gagnon , I. Daghlas , L. Zagkos , et al., “Mendelian Randomization Applied to Neurology: Promises and Challenges,” Neurology 102, no. 4 (2024): e209128. 38261980 10.1212/WNL.0000000000209128 PMC7615637

[31] C. Yu , Y. Sheng , F. Yu , et al., “Foxm1 Haploinsufficiency Drives Clonal Hematopoiesis and Promotes a Stress‐Related Transition to Hematologic Malignancy in Mice,” Journal of Clinical Investigation 133, no. 15 (2023): e163911. 37526082 10.1172/JCI163911 PMC10378147

[32] L. Gu , Q. Wang , G. Xu , and D. Liu , “Functional Genetic Variation in 3′UTR of PARP1 Indicates a Decreased Risk and a Better Severity of Ischemic Stroke,” International Journal of Neuroscience 134, no. 7 (2024): 804–809. 36448327 10.1080/00207454.2022.2151907

[33] L. Zimmerlin , A. Angarita , T. S. Park , et al., “Proteogenomic Reprogramming to a Functional Human Blastomere‐Like Stem Cell State via a PARP‐DUX4 Regulatory Axis,” Cell Reports 44, no. 5 (2025): 115671. 40338744 10.1016/j.celrep.2025.115671 PMC12541798

[34] J. H. Rowe , O. M. Delmonte , S. Keles , et al., “Patients With CD3G Mutations Reveal a Role for Human CD3γ in Treg Diversity and Suppressive Function,” Blood 131, no. 21 (2018): 2335–2344. 29653965 10.1182/blood-2018-02-835561 PMC5969384

[35] B. Suarez‐Álvarez , R. M. Rodríguez , K. Schlangen , et al., “Phenotypic Characteristics of Aged CD4+ CD28null T Lymphocytes Are Determined by Changes in the Whole‐Genome DNA Methylation Pattern,” Aging Cell 16, no. 2 (2017): 293–303. 28026094 10.1111/acel.12552 PMC5334526

[36] Q. Y. Lv , B. Wan , L. H. Guo , Y. Yang , X. M. Ren , and H. Zhang , “In Vivo Immunotoxicity of Perfluorooctane Sulfonate in BALB/c Mice: Identification of T‐Cell Receptor and Calcium‐Mediated Signaling Pathway Disruption Through Gene Expression Profiling of the Spleen,” Chemico‐Biological Interactions 240 (2015): 84–93. 26300304 10.1016/j.cbi.2015.07.015

[37] Y. W. Xu , Y. H. Peng , C. T. Liu , et al., “Machine Learning Technique‐Based Four‐Autoantibody Test for Early Detection of Esophageal Squamous Cell Carcinoma: A Multicenter, Retrospective Study With a Nested Case‐Control Study,” BMC Medicine 23, no. 1 (2025): 235. 40264204 10.1186/s12916-025-04066-2 PMC12016149

[38] J. Zhen , S. Liu , R. Y. L. Kam , et al., “Association of Beta‐2‐Microglobulin, Cystatin C and Lipocalin‐2 With Stroke Risk in the General Chinese Population,” Annals of Medicine 55, no. 1 (2023): 2203516. 37155257 10.1080/07853890.2023.2203516 PMC10167872

[39] P. M. Rist , M. C. Jiménez , and K. M. Rexrode , “Prospective Association Between β2‐Microglobulin Levels and Ischemic Stroke Risk Among Women,” Neurology 88, no. 23 (2017): 2176–2182. 28490653 10.1212/WNL.0000000000004006 PMC5467954

[40] M. Albitar , H. Zhang , A. Charifa , et al., “Using Cell‐Free RNA in Monitoring Immune System and the Demonstration of Significant Systemic Deficiency in Lymphoid and Myeloid Biomarkers in Patients With Cancer,” Journal of Clinical Oncology 42, no. 16_suppl (2024): 3048.

[41] Y. Weng , B. Liu , Z. Chen , et al., “Construction of a Diagnostic Model for Ischemic Stroke Based on Immune‐Related Genes,” Folia Neuropathologica 62, no. 2 (2024): 171–186. 39165204 10.5114/fn.2024.135846

[42] D. Wei , X. Chen , J. Xu , and W. He , “Identification of Molecular Subtypes of Ischaemic Stroke Based on Immune‐Related Genes and Weighted Co‐Expression Network Analysis,” IET Systems Biology 17, no. 2 (2023): 58–69. 36802116 10.1049/syb2.12059 PMC10116020

[43] Y. Yao , W. Ni , L. Feng , et al., “Comprehensive Immune Modulation Mechanisms of Angong Niuhuang Wan in Ischemic Stroke: Insights From Mass Cytometry Analysis,” CNS Neuroscience & Therapeutics 30, no. 7 (2024): e14849. 39075660 10.1111/cns.14849 PMC11286541

[44] B. Y. Lee , J. Chon , H. S. Kim , et al., “Association Between a Polymorphism in CASP3 and CASP9 Genes and Ischemic Stroke,” Annals of Rehabilitation Medicine 41, no. 2 (2017): 197–203. 28503451 10.5535/arm.2017.41.2.197 PMC5426270

[45] S. Corvigno , J. Yao , L. Zhao , et al., “Abstract 5473: Genomic Profiling of Chemotherapy‐Related Clonal Hematopoiesis in Patients With High‐Grade Serous Ovarian Cancer,” Cancer Research 83, no. 7_Supplement (2023): 5473.

[46] Z. Wang , J. Greenbaum , C. Qiu , et al., “Identification of Pleiotropic Genes Between Risk Factors of Stroke by Multivariate metaCCA Analysis,” Molecular Genetics and Genomics 295, no. 5 (2020): 1173–1185. 32474671 10.1007/s00438-020-01692-8 PMC7394724

[47] H. X. Chu , B. R. S. Broughton , H. A. Kim , S. Lee , G. R. Drummond , and C. G. Sobey , “Evidence That Ly6C(Hi) Monocytes Are Protective in Acute Ischemic Stroke by Promoting M2 Macrophage Polarization,” Stroke 46, no. 7 (2015): 1929–1937. 25999385 10.1161/STROKEAHA.115.009426

[48] J. Neumann , M. Riek‐Burchardt , J. Herz , et al., “Very‐Late‐Antigen‐4 (VLA‐4)‐Mediated Brain Invasion by Neutrophils Leads to Interactions With Microglia, Increased Ischemic Injury and Impaired Behavior in Experimental Stroke,” Acta Neuropathologica 129, no. 2 (2015): 259–277. 25391494 10.1007/s00401-014-1355-2

[49] J. Ruhnau , J. Schulze , A. Dressel , and A. Vogelgesang , “Thrombosis, Neuroinflammation, and Poststroke Infection: The Multifaceted Role of Neutrophils in Stroke,” Journal of Immunology Research 2017 (2017): 5140679. 28331857 10.1155/2017/5140679 PMC5346374

[50] Y. Ma , K. Zheng , C. Zhao , et al., “Microglia LILRB4 Upregulation Reduces Brain Damage After Acute Ischemic Stroke by Limiting CD8+ T Cell Recruitment,” Journal of Neuroinflammation 21, no. 1 (2024): 214. 39217343 10.1186/s12974-024-03206-4 PMC11366150

[51] G. L. Suidan , J. W. Dickerson , Y. Chen , et al., “CD8 T Cell‐Initiated Vascular Endothelial Growth Factor Expression Promotes Central Nervous System Vascular Permeability Under Neuroinflammatory Conditions,” Journal of Immunology 184, no. 2 (2010): 1031–1040. 10.4049/jimmunol.0902773 PMC2896014 20008293

[52] M. Wang , X. Zhang , R. Fan , and L. Zhang , “Causal Role of Immune Cell Traits in Stroke: A Mendelian Randomization Study,” Journal of Stroke and Cerebrovascular Diseases 33, no. 5 (2024): 107625. 38316285 10.1016/j.jstrokecerebrovasdis.2024.107625

[53] Y. E. Cho , H. Lee , H. R. Bae , et al., “Circulating Immune Cell Landscape in Patients Who Had Mild Ischaemic Stroke,” Stroke and Vascular Neurology 7, no. 4 (2022): 319–327. 35264400 10.1136/svn-2021-001224 PMC9453838

[54] Y. Feng , Y. Li , Y. Zhang , et al., “miR‐1224 Contributes to Ischemic Stroke‐Mediated Natural Killer Cell Dysfunction by Targeting Sp1 Signaling,” Journal of Neuroinflammation 18, no. 1 (2021): 133. 34118948 10.1186/s12974-021-02181-4 PMC8196447

[55] A. Jiao , C. Zhang , X. Wang , et al., “Single‐Cell Sequencing Reveals the Evolution of Immune Molecules Across Multiple Vertebrate Species,” Journal of Advanced Research 55 (2024): 73–87. 36871615 10.1016/j.jare.2023.02.017 PMC10770119

