# Supplementary material for: Genetic Associations of Clonal Hematopoiesis With Cardioembolic Stroke: Insights From Genome‐Wide Mendelian Randomization, Bulk RNA, Single‐Cell RNA Sequencing
Source: CNS Neurosci Ther. 2025 Jul 23;31(7):e70515. doi: 10.1111/cns.70515 (PMC12287382; doi:10.1111/cns.70515)
Supplement: Supplementary file 1 — Data S1: [file CNS-31-e70515-s001.docx]

# Supplementary Materials

## Supplementary Methods

### Supplementary Method 1: Gene Set Enrichment Analysis of Functional Pathways, microRNAs, and Transcription Factors Between High and Low Expression Groups of Key Genes

The samples were divided into high and low expression groups based on the expression levels of the key genes, PARP1 and CD3G. We downloaded the gene set files “c5.go.Hs.symbols.gmt” “c3.mir.v2024.1.Hs.symbols.gmt” and “c3.tft.v2024.1.Hs.symbols.gmt” from the MSigDB database. The R package “clusterProfiler” was used to calculate the enrichment scores for functional pathways, microRNAs, and transcription factors (TFs) in the high and low expression groups.

As shown in Figure S1A and S1B, the high expression group for PARP1 was primarily enriched in ANTIGEN RECEPTOR MEDIATED SIGNALING PATHWAY, LYMPHOCYTE ACTIVATION, MONONUCLEAR CELL DIFFERENTIATION, T CELL DIFFERENTIATION, and NUCLEOLUS compared to the low expression group, indicating that immune responses were activated with elevated PARP1 expression. Similarly, the high expression group for CD3G was enriched in RIBONUCLEOPROTEIN COMPLEX BIOGENESIS, MITOCHONDRIAL PROTEIN CONTAINING COMPLEX, RIBOSOMAL SUBUNIT, RIBOSOME, and STRUCTURAL CONSTITUENT OF RIBOSOME, while the low expression group was associated with secretory pathways (Figure S1C and S1D).

Additionally, the upstream microRNAs enriched in the high and low expression groups of the key genes are shown in Figure S1E, S1F, S1G, and S1H. Figure S1I, S1J, S1K, and S1L illustrate the differences in TF enrichment between the two groups.

### Supplementary Method 2: MR Analysis of eQTLs, pQTLs, and SMR for the Feature Genes

The results of the eQTL and pQTL analyses are presented in Table S5. At the gene expression level, CASP3 demonstrated strong evidence of a causal association with cardioembolic stroke (CES) (P-value = 0.037). However, no significant association was found at the protein expression level. Additionally, the SMR analysis results, shown in Table S6, further confirmed that CASP3 expression is strongly associated with CES.

### Supplementary Method 3: Drug Enrichment Analysis and Molecular Docking

We conducted a drug enrichment analysis using drug-gene interactions from the DSigDB database. The top four enriched drugs were nitric oxide, pamidronate, fludarabine, and ciprofloxacin, as shown in Figure S4A. Nitric oxide, which bound to the most genes, was selected for further analysis.

We then searched for 'nitric oxide' in the PubChem database and downloaded its two-dimensional structure. Additionally, we retrieved the PDB formats for PARP1 (6ntu), B2M (1hsb), CD8A (2hp4), CASP3 (1nme), and CASP9 (4rhw) from the Protein Data Bank. Using the CB-Dock2 online platform, we performed cavity-detection guided blind docking between nitric oxide and these target genes, although the docking results were suboptimal (Figure S4B-S4F).

### Supplementary Method 4: Single-cell analysis of monocyte subtypes

As illustrated in Figure 6A, a significant increase in monocyte count was observed following IS onset. Initial analysis revealed no statistically significant differences in PARP1 and CD3G expression profiles between IS and sham-operated groups (*p* > 0.05), prompting further investigation through monocyte subpopulation analysis. Utilizing t- t-SNE dimensionality reduction, we identified four distinct monocyte clusters (Figure S5A). These subpopulations were subsequently classified as classical, non-classical, intermediate, and progenitor monocytes through marker gene analysis, employing characteristic monocyte markers CX3CR1, CCR2, and CD14 for phenotypic annotation (Figure S5B). Comparative analysis of PARP1 and CD3G expression patterns across all identified monocyte subtypes (IS vs. control) demonstrated no statistically significant intergroup differences (*p* > 0.05 for all comparisons), as detailed in Figures S5C and S5D.

## Supplementary Figures

**Figure S1 |** (A-D) Gene set enrichment analysis of functional pathways; (E-H) Gene set enrichment analysis of microRNAs; (I-L) Gene set enrichment analysis of TFs in the high and low expression group of PARP1 and CD3G.


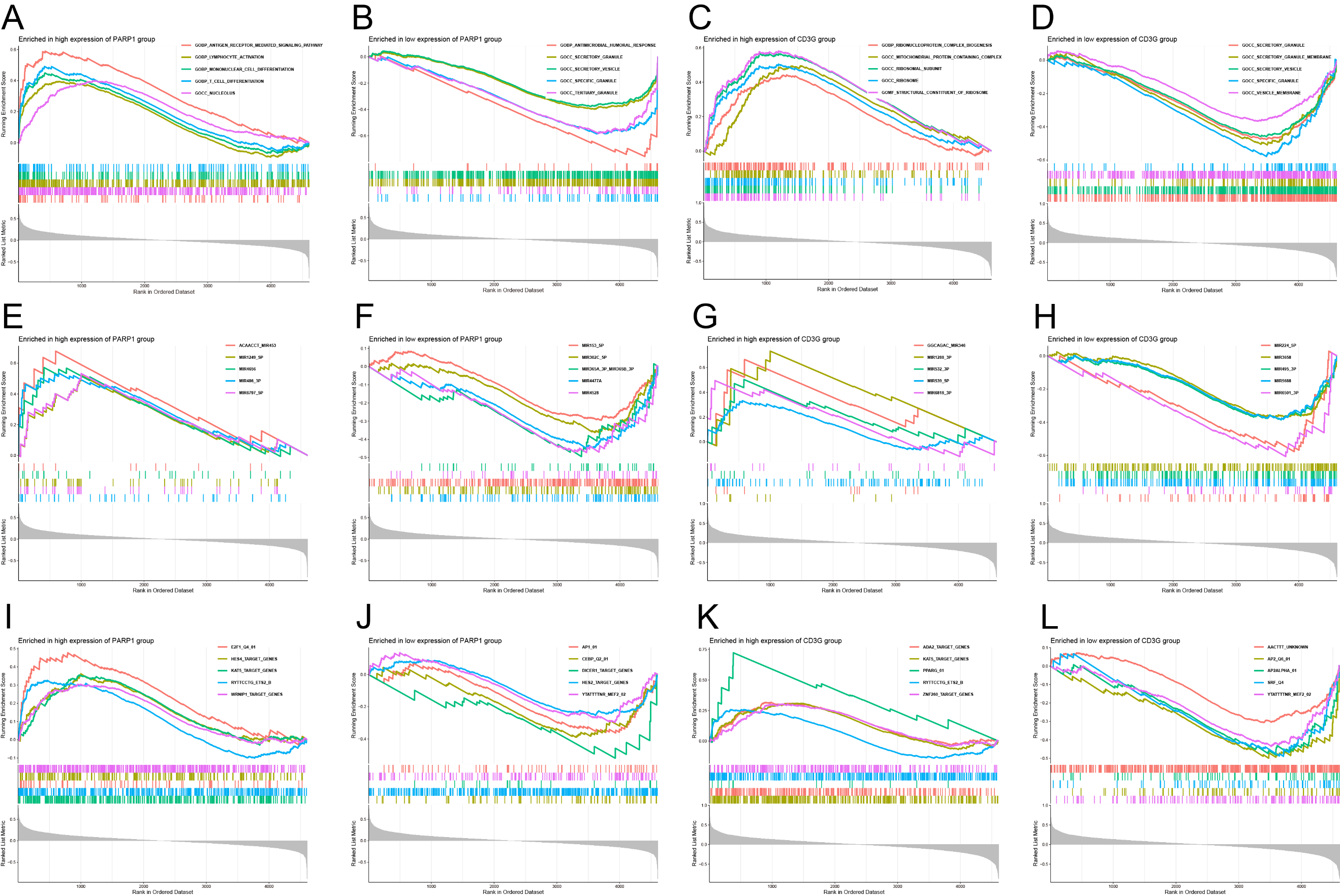


**Figure S2** | (A) PPI network of genes related to PARP1; (B) PPI network of genes related to CD3G; (C) Circle plot of GO analysis; (D) Bubble plot of GO analysis; (E) Bubble plot of KEGG analysis.


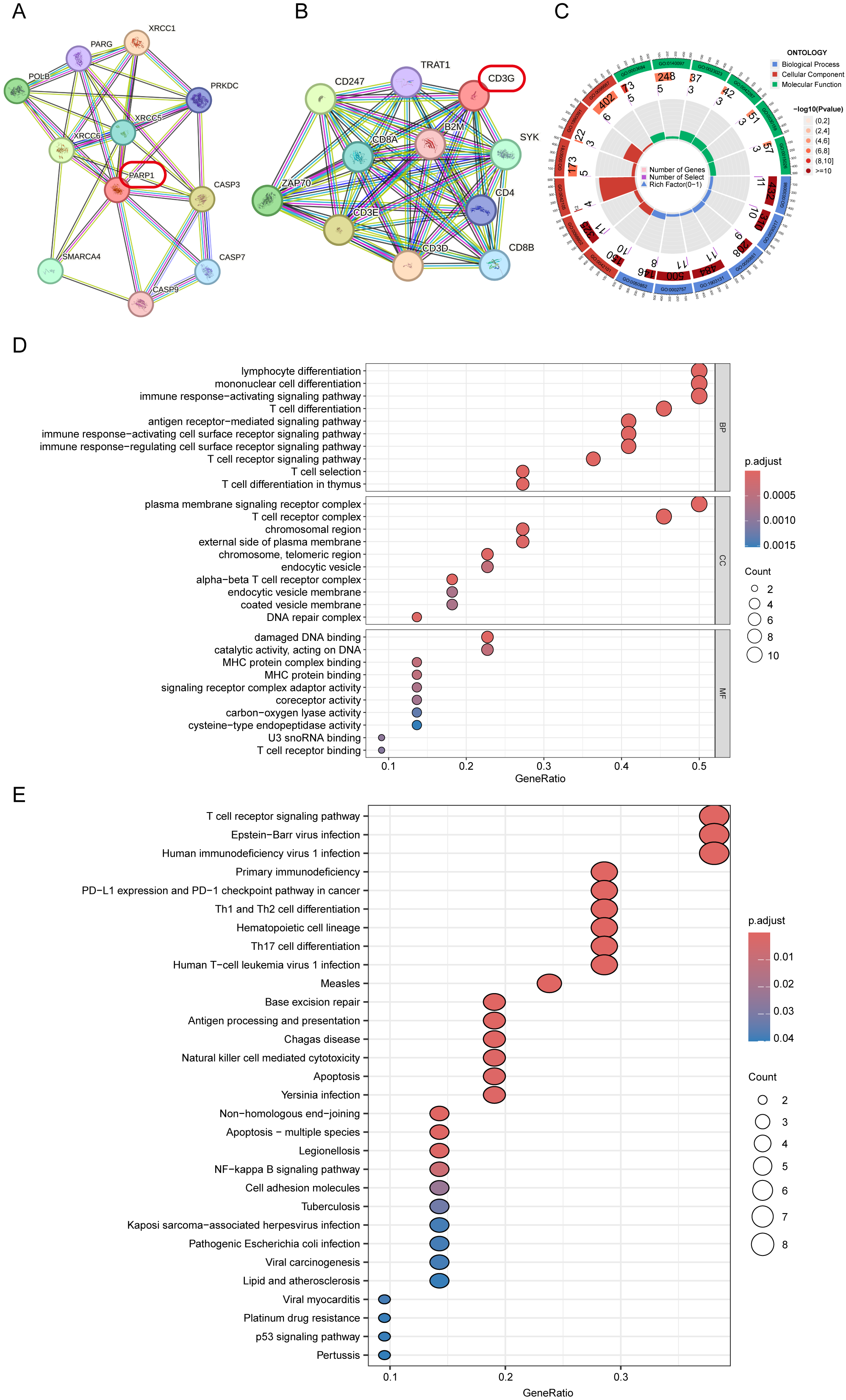


**Figure S3** | (A) The confusion matrix of the training sets; (B) The confusion matrix of the validation sets; (C) Dot plot and distribution plot for the correlation analysis of the feature genes. (D) Q-Q plot of the normality check of the expression of the feature genes.


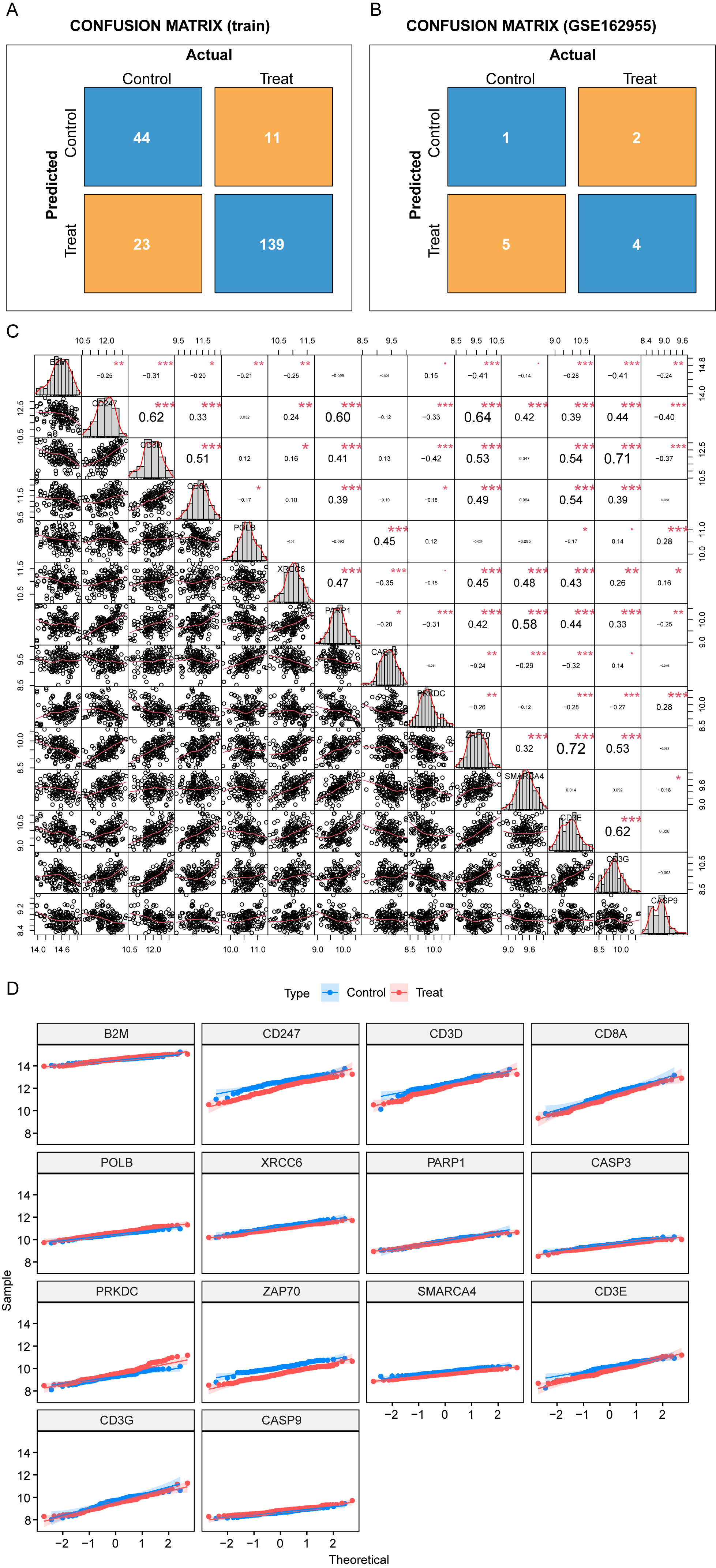


**Figure S4** | (A) Bubble plot for the drug enrichment analysis of the feature genes; (B) Molecular docking between nitric oxide and PARP1; (C) Molecular docking between nitric oxide and B2M; (D) Molecular docking between nitric oxide and CD8A; (E) Molecular docking between nitric oxide and CASP3; (F) Molecular docking between nitric oxide and CASP9.


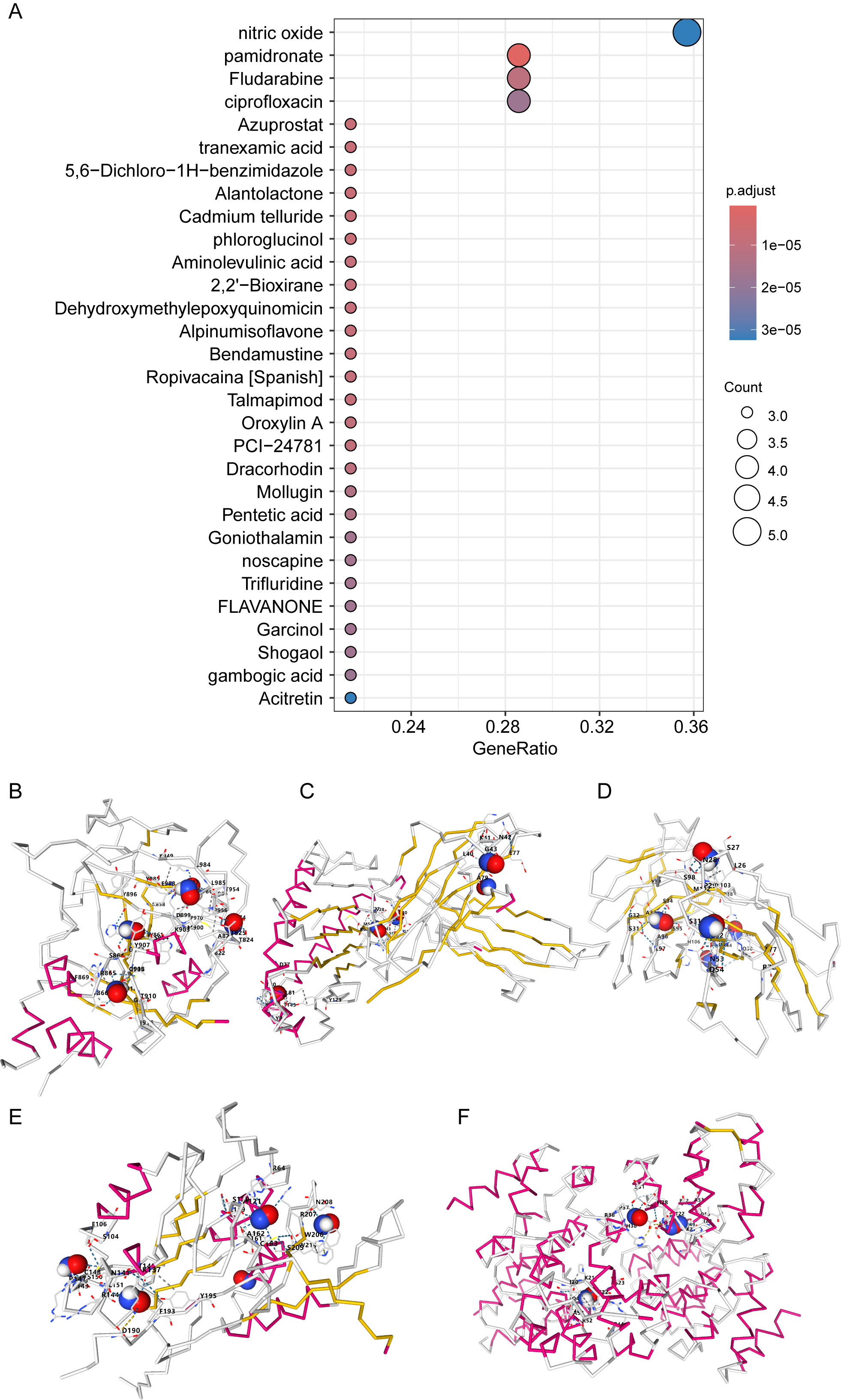


**Figure S5** | (A) TSNE plots of monocyte groups (before cell annotation); (B) TSNE plots of monocyte groups between stroke and control group (after cell annotation); (C) Box plot of the expression of PARP1 in different monocyte subgroups between the IS and control (sham) groups; (D) Box plot of the expression of CD3G in different monocyte subgroups between the IS and control (sham) groups.


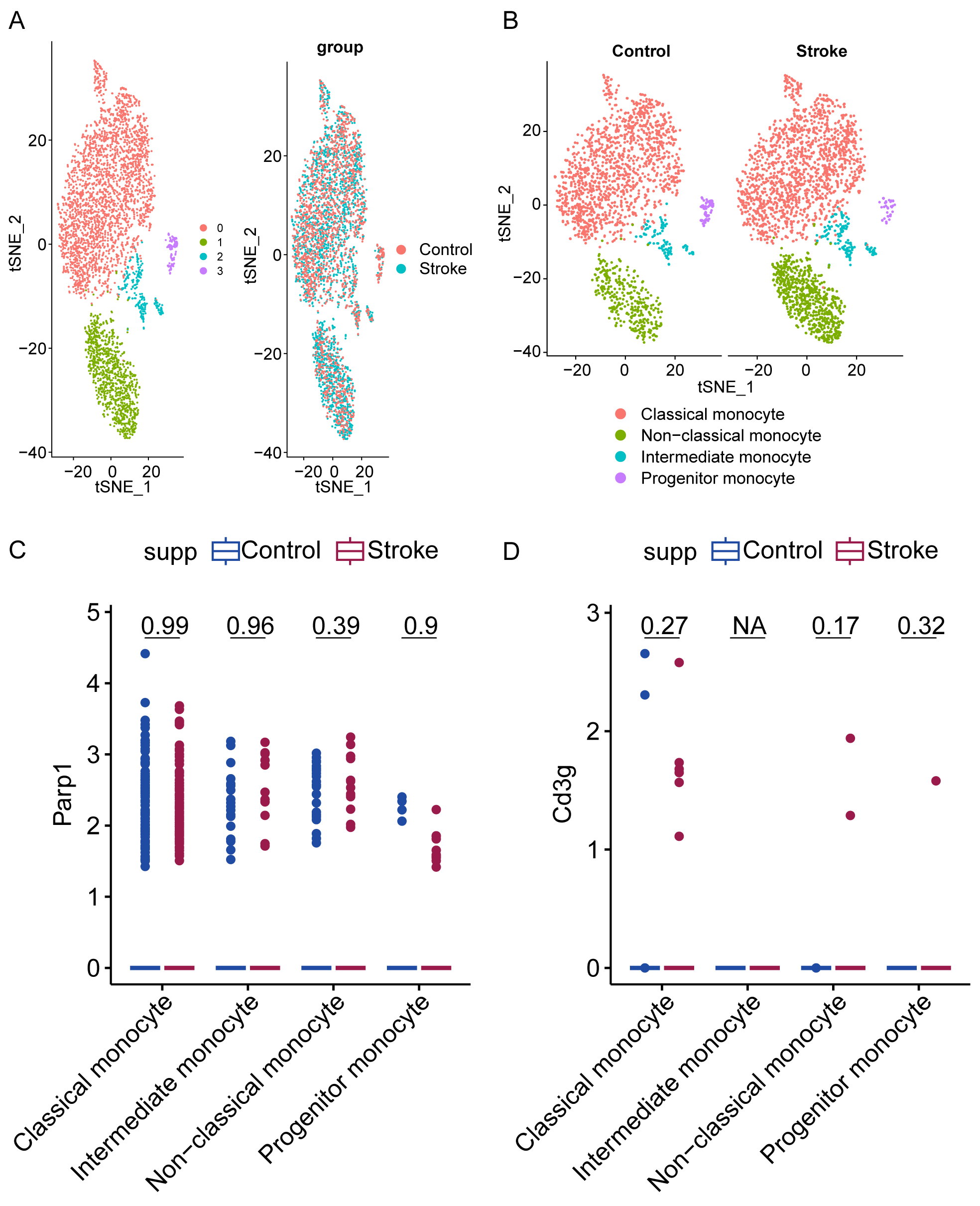


## Supplementary Tables

**Table S1** | The F-statistics for all instrumental variables.

F=F-statistic; meanf=means of F-statistics.

| **SNP** | **Exposure** | **Outcome** | **F** | **meanf** |
| --- | --- | --- | --- | --- |
| rs1029411 | Clonal hematopoiesis (overall) | AIS | 19.809 | 29.387 |
| rs11212666 | Clonal hematopoiesis (overall) | AIS | 34.515 | 29.387 |
| rs113892140 | Clonal hematopoiesis (overall) | AIS | 20.682 | 29.387 |
| rs115137644 | Clonal hematopoiesis (overall) | AIS | 20.720 | 29.387 |
| rs11611479 | Clonal hematopoiesis (overall) | AIS | 23.350 | 29.387 |
| rs11695542 | Clonal hematopoiesis (overall) | AIS | 20.366 | 29.387 |
| rs117314695 | Clonal hematopoiesis (overall) | AIS | 21.544 | 29.387 |
| rs117472123 | Clonal hematopoiesis (overall) | AIS | 20.970 | 29.387 |
| rs117485768 | Clonal hematopoiesis (overall) | AIS | 20.089 | 29.387 |
| rs118121072 | Clonal hematopoiesis (overall) | AIS | 20.306 | 29.387 |
| rs11880163 | Clonal hematopoiesis (overall) | AIS | 20.466 | 29.387 |
| rs12632224 | Clonal hematopoiesis (overall) | AIS | 35.817 | 29.387 |
| rs12893938 | Clonal hematopoiesis (overall) | AIS | 21.603 | 29.387 |
| rs12904882 | Clonal hematopoiesis (overall) | AIS | 20.947 | 29.387 |
| rs13130545 | Clonal hematopoiesis (overall) | AIS | 29.390 | 29.387 |
| rs138239047 | Clonal hematopoiesis (overall) | AIS | 20.519 | 29.387 |
| rs143144207 | Clonal hematopoiesis (overall) | AIS | 20.630 | 29.387 |
| rs17696019 | Clonal hematopoiesis (overall) | AIS | 20.088 | 29.387 |
| rs2077810 | Clonal hematopoiesis (overall) | AIS | 19.806 | 29.387 |
| rs2853677 | Clonal hematopoiesis (overall) | AIS | 222.544 | 29.387 |
| rs35452836 | Clonal hematopoiesis (overall) | AIS | 34.018 | 29.387 |
| rs45564234 | Clonal hematopoiesis (overall) | AIS | 20.533 | 29.387 |
| rs6026564 | Clonal hematopoiesis (overall) | AIS | 19.559 | 29.387 |
| rs61392716 | Clonal hematopoiesis (overall) | AIS | 21.891 | 29.387 |
| rs62105066 | Clonal hematopoiesis (overall) | AIS | 21.475 | 29.387 |
| rs6442623 | Clonal hematopoiesis (overall) | AIS | 21.926 | 29.387 |
| rs6841004 | Clonal hematopoiesis (overall) | AIS | 20.178 | 29.387 |
| rs7207177 | Clonal hematopoiesis (overall) | AIS | 23.482 | 29.387 |
| rs7232918 | Clonal hematopoiesis (overall) | AIS | 24.934 | 29.387 |
| rs72755524 | Clonal hematopoiesis (overall) | AIS | 19.682 | 29.387 |
| rs759656 | Clonal hematopoiesis (overall) | AIS | 29.170 | 29.387 |
| rs1021797 | Clonal hematopoiesis (DNMT3A mutation) | AIS | 25.442 | 31.140 |
| rs10864219 | Clonal hematopoiesis (DNMT3A mutation) | AIS | 24.350 | 31.140 |
| rs10942770 | Clonal hematopoiesis (DNMT3A mutation) | AIS | 24.857 | 31.140 |
| rs11257356 | Clonal hematopoiesis (DNMT3A mutation) | AIS | 20.083 | 31.140 |
| rs112675842 | Clonal hematopoiesis (DNMT3A mutation) | AIS | 23.038 | 31.140 |
| rs116911541 | Clonal hematopoiesis (DNMT3A mutation) | AIS | 20.479 | 31.140 |
| rs117475117 | Clonal hematopoiesis (DNMT3A mutation) | AIS | 21.040 | 31.140 |
| rs11880163 | Clonal hematopoiesis (DNMT3A mutation) | AIS | 21.299 | 31.140 |
| rs12524502 | Clonal hematopoiesis (DNMT3A mutation) | AIS | 70.969 | 31.140 |
| rs12634120 | Clonal hematopoiesis (DNMT3A mutation) | AIS | 25.660 | 31.140 |
| rs12692566 | Clonal hematopoiesis (DNMT3A mutation) | AIS | 23.719 | 31.140 |
| rs12821838 | Clonal hematopoiesis (DNMT3A mutation) | AIS | 20.261 | 31.140 |
| rs228606 | Clonal hematopoiesis (DNMT3A mutation) | AIS | 51.301 | 31.140 |
| rs2296312 | Clonal hematopoiesis (DNMT3A mutation) | AIS | 33.044 | 31.140 |
| rs2841648 | Clonal hematopoiesis (DNMT3A mutation) | AIS | 26.926 | 31.140 |
| rs2853677 | Clonal hematopoiesis (DNMT3A mutation) | AIS | 172.379 | 31.140 |
| rs28689133 | Clonal hematopoiesis (DNMT3A mutation) | AIS | 19.517 | 31.140 |
| rs34795680 | Clonal hematopoiesis (DNMT3A mutation) | AIS | 22.035 | 31.140 |
| rs4234481 | Clonal hematopoiesis (DNMT3A mutation) | AIS | 21.773 | 31.140 |
| rs4662525 | Clonal hematopoiesis (DNMT3A mutation) | AIS | 22.079 | 31.140 |
| rs4669004 | Clonal hematopoiesis (DNMT3A mutation) | AIS | 19.417 | 31.140 |
| rs4779071 | Clonal hematopoiesis (DNMT3A mutation) | AIS | 19.926 | 31.140 |
| rs62237573 | Clonal hematopoiesis (DNMT3A mutation) | AIS | 23.774 | 31.140 |
| rs759656 | Clonal hematopoiesis (DNMT3A mutation) | AIS | 24.364 | 31.140 |
| rs77481539 | Clonal hematopoiesis (DNMT3A mutation) | AIS | 22.486 | 31.140 |
| rs77802645 | Clonal hematopoiesis (DNMT3A mutation) | AIS | 19.476 | 31.140 |
| rs8088824 | Clonal hematopoiesis (DNMT3A mutation) | AIS | 30.300 | 31.140 |
| rs9649961 | Clonal hematopoiesis (DNMT3A mutation) | AIS | 21.921 | 31.140 |
| rs10131341 | Clonal hematopoiesis (TET2 mutation) | AIS | 38.148 | 24.407 |
| rs10782071 | Clonal hematopoiesis (TET2 mutation) | AIS | 23.276 | 24.407 |
| rs11111098 | Clonal hematopoiesis (TET2 mutation) | AIS | 24.004 | 24.407 |
| rs11132125 | Clonal hematopoiesis (TET2 mutation) | AIS | 20.724 | 24.407 |
| rs117150600 | Clonal hematopoiesis (TET2 mutation) | AIS | 19.995 | 24.407 |
| rs12791065 | Clonal hematopoiesis (TET2 mutation) | AIS | 24.724 | 24.407 |
| rs12890144 | Clonal hematopoiesis (TET2 mutation) | AIS | 19.644 | 24.407 |
| rs189506759 | Clonal hematopoiesis (TET2 mutation) | AIS | 19.633 | 24.407 |
| rs2736100 | Clonal hematopoiesis (TET2 mutation) | AIS | 71.774 | 24.407 |
| rs28664269 | Clonal hematopoiesis (TET2 mutation) | AIS | 19.792 | 24.407 |
| rs35484216 | Clonal hematopoiesis (TET2 mutation) | AIS | 22.178 | 24.407 |
| rs41272399 | Clonal hematopoiesis (TET2 mutation) | AIS | 19.534 | 24.407 |
| rs4145796 | Clonal hematopoiesis (TET2 mutation) | AIS | 20.130 | 24.407 |
| rs56327306 | Clonal hematopoiesis (TET2 mutation) | AIS | 21.175 | 24.407 |
| rs57019067 | Clonal hematopoiesis (TET2 mutation) | AIS | 20.911 | 24.407 |
| rs59108483 | Clonal hematopoiesis (TET2 mutation) | AIS | 19.871 | 24.407 |
| rs61951330 | Clonal hematopoiesis (TET2 mutation) | AIS | 19.993 | 24.407 |
| rs73214874 | Clonal hematopoiesis (TET2 mutation) | AIS | 20.799 | 24.407 |
| rs7739373 | Clonal hematopoiesis (TET2 mutation) | AIS | 20.196 | 24.407 |
| rs7816774 | Clonal hematopoiesis (TET2 mutation) | AIS | 20.991 | 24.407 |
| rs78729418 | Clonal hematopoiesis (TET2 mutation) | AIS | 20.553 | 24.407 |
| rs78849954 | Clonal hematopoiesis (TET2 mutation) | AIS | 19.560 | 24.407 |
| rs79020059 | Clonal hematopoiesis (TET2 mutation) | AIS | 22.504 | 24.407 |
| rs79633204 | Clonal hematopoiesis (TET2 mutation) | AIS | 37.592 | 24.407 |
| rs858339 | Clonal hematopoiesis (TET2 mutation) | AIS | 22.478 | 24.407 |
| rs112836922 | Clonal hematopoiesis (large clone) | AIS | 20.772 | 26.317 |
| rs117066841 | Clonal hematopoiesis (large clone) | AIS | 19.860 | 26.317 |
| rs117942522 | Clonal hematopoiesis (large clone) | AIS | 21.291 | 26.317 |
| rs12615707 | Clonal hematopoiesis (large clone) | AIS | 21.510 | 26.317 |
| rs12632224 | Clonal hematopoiesis (large clone) | AIS | 29.101 | 26.317 |
| rs140599943 | Clonal hematopoiesis (large clone) | AIS | 22.898 | 26.317 |
| rs144815420 | Clonal hematopoiesis (large clone) | AIS | 21.173 | 26.317 |
| rs146838695 | Clonal hematopoiesis (large clone) | AIS | 19.983 | 26.317 |
| rs148557733 | Clonal hematopoiesis (large clone) | AIS | 20.368 | 26.317 |
| rs150846798 | Clonal hematopoiesis (large clone) | AIS | 25.945 | 26.317 |
| rs2468948 | Clonal hematopoiesis (large clone) | AIS | 23.046 | 26.317 |
| rs474943 | Clonal hematopoiesis (large clone) | AIS | 20.556 | 26.317 |
| rs4906361 | Clonal hematopoiesis (large clone) | AIS | 22.900 | 26.317 |
| rs633137 | Clonal hematopoiesis (large clone) | AIS | 22.191 | 26.317 |
| rs7207177 | Clonal hematopoiesis (large clone) | AIS | 24.885 | 26.317 |
| rs724952 | Clonal hematopoiesis (large clone) | AIS | 20.633 | 26.317 |
| rs73020212 | Clonal hematopoiesis (large clone) | AIS | 21.809 | 26.317 |
| rs74893169 | Clonal hematopoiesis (large clone) | AIS | 20.997 | 26.317 |
| rs76887998 | Clonal hematopoiesis (large clone) | AIS | 22.655 | 26.317 |
| rs7705526 | Clonal hematopoiesis (large clone) | AIS | 116.907 | 26.317 |
| rs78970714 | Clonal hematopoiesis (large clone) | AIS | 19.756 | 26.317 |
| rs8066979 | Clonal hematopoiesis (large clone) | AIS | 19.742 | 26.317 |
| rs10790722 | Clonal hematopoiesis (small clone) | AIS | 20.009 | 25.508 |
| rs10980423 | Clonal hematopoiesis (small clone) | AIS | 20.006 | 25.508 |
| rs11038147 | Clonal hematopoiesis (small clone) | AIS | 20.688 | 25.508 |
| rs11055736 | Clonal hematopoiesis (small clone) | AIS | 20.185 | 25.508 |
| rs112678842 | Clonal hematopoiesis (small clone) | AIS | 19.972 | 25.508 |
| rs115984969 | Clonal hematopoiesis (small clone) | AIS | 21.811 | 25.508 |
| rs116944293 | Clonal hematopoiesis (small clone) | AIS | 21.110 | 25.508 |
| rs118024410 | Clonal hematopoiesis (small clone) | AIS | 21.141 | 25.508 |
| rs13130545 | Clonal hematopoiesis (small clone) | AIS | 22.341 | 25.508 |
| rs138239047 | Clonal hematopoiesis (small clone) | AIS | 20.360 | 25.508 |
| rs141030142 | Clonal hematopoiesis (small clone) | AIS | 20.089 | 25.508 |
| rs141271281 | Clonal hematopoiesis (small clone) | AIS | 20.126 | 25.508 |
| rs17681776 | Clonal hematopoiesis (small clone) | AIS | 20.711 | 25.508 |
| rs182606956 | Clonal hematopoiesis (small clone) | AIS | 20.747 | 25.508 |
| rs188129997 | Clonal hematopoiesis (small clone) | AIS | 22.095 | 25.508 |
| rs1904398 | Clonal hematopoiesis (small clone) | AIS | 19.701 | 25.508 |
| rs2853677 | Clonal hematopoiesis (small clone) | AIS | 120.370 | 25.508 |
| rs35452836 | Clonal hematopoiesis (small clone) | AIS | 36.219 | 25.508 |
| rs3743840 | Clonal hematopoiesis (small clone) | AIS | 19.830 | 25.508 |
| rs4850108 | Clonal hematopoiesis (small clone) | AIS | 22.042 | 25.508 |
| rs6026564 | Clonal hematopoiesis (small clone) | AIS | 24.644 | 25.508 |
| rs6580637 | Clonal hematopoiesis (small clone) | AIS | 19.785 | 25.508 |
| rs7129527 | Clonal hematopoiesis (small clone) | AIS | 20.589 | 25.508 |
| rs72755524 | Clonal hematopoiesis (small clone) | AIS | 30.114 | 25.508 |
| rs75760715 | Clonal hematopoiesis (small clone) | AIS | 21.586 | 25.508 |
| rs759656 | Clonal hematopoiesis (small clone) | AIS | 24.441 | 25.508 |
| rs7702021 | Clonal hematopoiesis (small clone) | AIS | 21.248 | 25.508 |
| rs79406832 | Clonal hematopoiesis (small clone) | AIS | 22.253 | 25.508 |
| rs1029411 | Clonal hematopoiesis (overall) | LAS | 19.809 | 29.126 |
| rs11212666 | Clonal hematopoiesis (overall) | LAS | 34.515 | 29.126 |
| rs113892140 | Clonal hematopoiesis (overall) | LAS | 20.682 | 29.126 |
| rs115137644 | Clonal hematopoiesis (overall) | LAS | 20.720 | 29.126 |
| rs11611479 | Clonal hematopoiesis (overall) | LAS | 23.350 | 29.126 |
| rs11695542 | Clonal hematopoiesis (overall) | LAS | 20.366 | 29.126 |
| rs117314695 | Clonal hematopoiesis (overall) | LAS | 21.544 | 29.126 |
| rs117472123 | Clonal hematopoiesis (overall) | LAS | 20.970 | 29.126 |
| rs117485768 | Clonal hematopoiesis (overall) | LAS | 20.089 | 29.126 |
| rs118121072 | Clonal hematopoiesis (overall) | LAS | 20.306 | 29.126 |
| rs11880163 | Clonal hematopoiesis (overall) | LAS | 20.466 | 29.126 |
| rs12632224 | Clonal hematopoiesis (overall) | LAS | 35.817 | 29.126 |
| rs12893938 | Clonal hematopoiesis (overall) | LAS | 21.603 | 29.126 |
| rs12904882 | Clonal hematopoiesis (overall) | LAS | 20.947 | 29.126 |
| rs13130545 | Clonal hematopoiesis (overall) | LAS | 29.390 | 29.126 |
| rs138239047 | Clonal hematopoiesis (overall) | LAS | 20.519 | 29.126 |
| rs143144207 | Clonal hematopoiesis (overall) | LAS | 20.630 | 29.126 |
| rs17696019 | Clonal hematopoiesis (overall) | LAS | 20.088 | 29.126 |
| rs2077810 | Clonal hematopoiesis (overall) | LAS | 19.806 | 29.126 |
| rs2853677 | Clonal hematopoiesis (overall) | LAS | 222.544 | 29.126 |
| rs35452836 | Clonal hematopoiesis (overall) | LAS | 34.018 | 29.126 |
| rs45564234 | Clonal hematopoiesis (overall) | LAS | 20.533 | 29.126 |
| rs6026564 | Clonal hematopoiesis (overall) | LAS | 19.559 | 29.126 |
| rs61392716 | Clonal hematopoiesis (overall) | LAS | 21.891 | 29.126 |
| rs62105066 | Clonal hematopoiesis (overall) | LAS | 21.475 | 29.126 |
| rs6442623 | Clonal hematopoiesis (overall) | LAS | 21.926 | 29.126 |
| rs6841004 | Clonal hematopoiesis (overall) | LAS | 20.178 | 29.126 |
| rs7207177 | Clonal hematopoiesis (overall) | LAS | 23.482 | 29.126 |
| rs7232918 | Clonal hematopoiesis (overall) | LAS | 24.934 | 29.126 |
| rs72755524 | Clonal hematopoiesis (overall) | LAS | 19.682 | 29.126 |
| rs759656 | Clonal hematopoiesis (overall) | LAS | 29.170 | 29.126 |
| rs80019846 | Clonal hematopoiesis (overall) | LAS | 21.010 | 29.126 |
| rs1021797 | Clonal hematopoiesis (DNMT3A mutation) | LAS | 25.442 | 31.140 |
| rs10864219 | Clonal hematopoiesis (DNMT3A mutation) | LAS | 24.350 | 31.140 |
| rs10942770 | Clonal hematopoiesis (DNMT3A mutation) | LAS | 24.857 | 31.140 |
| rs11257356 | Clonal hematopoiesis (DNMT3A mutation) | LAS | 20.083 | 31.140 |
| rs112675842 | Clonal hematopoiesis (DNMT3A mutation) | LAS | 23.038 | 31.140 |
| rs116911541 | Clonal hematopoiesis (DNMT3A mutation) | LAS | 20.479 | 31.140 |
| rs117475117 | Clonal hematopoiesis (DNMT3A mutation) | LAS | 21.040 | 31.140 |
| rs11880163 | Clonal hematopoiesis (DNMT3A mutation) | LAS | 21.299 | 31.140 |
| rs12524502 | Clonal hematopoiesis (DNMT3A mutation) | LAS | 70.969 | 31.140 |
| rs12634120 | Clonal hematopoiesis (DNMT3A mutation) | LAS | 25.660 | 31.140 |
| rs12692566 | Clonal hematopoiesis (DNMT3A mutation) | LAS | 23.719 | 31.140 |
| rs12821838 | Clonal hematopoiesis (DNMT3A mutation) | LAS | 20.261 | 31.140 |
| rs228606 | Clonal hematopoiesis (DNMT3A mutation) | LAS | 51.301 | 31.140 |
| rs2296312 | Clonal hematopoiesis (DNMT3A mutation) | LAS | 33.044 | 31.140 |
| rs2841648 | Clonal hematopoiesis (DNMT3A mutation) | LAS | 26.926 | 31.140 |
| rs2853677 | Clonal hematopoiesis (DNMT3A mutation) | LAS | 172.379 | 31.140 |
| rs28689133 | Clonal hematopoiesis (DNMT3A mutation) | LAS | 19.517 | 31.140 |
| rs34795680 | Clonal hematopoiesis (DNMT3A mutation) | LAS | 22.035 | 31.140 |
| rs4234481 | Clonal hematopoiesis (DNMT3A mutation) | LAS | 21.773 | 31.140 |
| rs4662525 | Clonal hematopoiesis (DNMT3A mutation) | LAS | 22.079 | 31.140 |
| rs4669004 | Clonal hematopoiesis (DNMT3A mutation) | LAS | 19.417 | 31.140 |
| rs4779071 | Clonal hematopoiesis (DNMT3A mutation) | LAS | 19.926 | 31.140 |
| rs62237573 | Clonal hematopoiesis (DNMT3A mutation) | LAS | 23.774 | 31.140 |
| rs759656 | Clonal hematopoiesis (DNMT3A mutation) | LAS | 24.364 | 31.140 |
| rs77481539 | Clonal hematopoiesis (DNMT3A mutation) | LAS | 22.486 | 31.140 |
| rs77802645 | Clonal hematopoiesis (DNMT3A mutation) | LAS | 19.476 | 31.140 |
| rs8088824 | Clonal hematopoiesis (DNMT3A mutation) | LAS | 30.300 | 31.140 |
| rs9649961 | Clonal hematopoiesis (DNMT3A mutation) | LAS | 21.921 | 31.140 |
| rs10131341 | Clonal hematopoiesis (TET2 mutation) | LAS | 38.148 | 24.407 |
| rs10782071 | Clonal hematopoiesis (TET2 mutation) | LAS | 23.276 | 24.407 |
| rs11111098 | Clonal hematopoiesis (TET2 mutation) | LAS | 24.004 | 24.407 |
| rs11132125 | Clonal hematopoiesis (TET2 mutation) | LAS | 20.724 | 24.407 |
| rs117150600 | Clonal hematopoiesis (TET2 mutation) | LAS | 19.995 | 24.407 |
| rs12791065 | Clonal hematopoiesis (TET2 mutation) | LAS | 24.724 | 24.407 |
| rs12890144 | Clonal hematopoiesis (TET2 mutation) | LAS | 19.644 | 24.407 |
| rs189506759 | Clonal hematopoiesis (TET2 mutation) | LAS | 19.633 | 24.407 |
| rs2736100 | Clonal hematopoiesis (TET2 mutation) | LAS | 71.774 | 24.407 |
| rs28664269 | Clonal hematopoiesis (TET2 mutation) | LAS | 19.792 | 24.407 |
| rs35484216 | Clonal hematopoiesis (TET2 mutation) | LAS | 22.178 | 24.407 |
| rs41272399 | Clonal hematopoiesis (TET2 mutation) | LAS | 19.534 | 24.407 |
| rs4145796 | Clonal hematopoiesis (TET2 mutation) | LAS | 20.130 | 24.407 |
| rs56327306 | Clonal hematopoiesis (TET2 mutation) | LAS | 21.175 | 24.407 |
| rs57019067 | Clonal hematopoiesis (TET2 mutation) | LAS | 20.911 | 24.407 |
| rs59108483 | Clonal hematopoiesis (TET2 mutation) | LAS | 19.871 | 24.407 |
| rs61951330 | Clonal hematopoiesis (TET2 mutation) | LAS | 19.993 | 24.407 |
| rs73214874 | Clonal hematopoiesis (TET2 mutation) | LAS | 20.799 | 24.407 |
| rs7739373 | Clonal hematopoiesis (TET2 mutation) | LAS | 20.196 | 24.407 |
| rs7816774 | Clonal hematopoiesis (TET2 mutation) | LAS | 20.991 | 24.407 |
| rs78729418 | Clonal hematopoiesis (TET2 mutation) | LAS | 20.553 | 24.407 |
| rs78849954 | Clonal hematopoiesis (TET2 mutation) | LAS | 19.560 | 24.407 |
| rs79020059 | Clonal hematopoiesis (TET2 mutation) | LAS | 22.504 | 24.407 |
| rs79633204 | Clonal hematopoiesis (TET2 mutation) | LAS | 37.592 | 24.407 |
| rs858339 | Clonal hematopoiesis (TET2 mutation) | LAS | 22.478 | 24.407 |
| rs112836922 | Clonal hematopoiesis (large clone) | LAS | 20.772 | 26.317 |
| rs117066841 | Clonal hematopoiesis (large clone) | LAS | 19.860 | 26.317 |
| rs117942522 | Clonal hematopoiesis (large clone) | LAS | 21.291 | 26.317 |
| rs12615707 | Clonal hematopoiesis (large clone) | LAS | 21.510 | 26.317 |
| rs12632224 | Clonal hematopoiesis (large clone) | LAS | 29.101 | 26.317 |
| rs140599943 | Clonal hematopoiesis (large clone) | LAS | 22.898 | 26.317 |
| rs144815420 | Clonal hematopoiesis (large clone) | LAS | 21.173 | 26.317 |
| rs146838695 | Clonal hematopoiesis (large clone) | LAS | 19.983 | 26.317 |
| rs148557733 | Clonal hematopoiesis (large clone) | LAS | 20.368 | 26.317 |
| rs150846798 | Clonal hematopoiesis (large clone) | LAS | 25.945 | 26.317 |
| rs2468948 | Clonal hematopoiesis (large clone) | LAS | 23.046 | 26.317 |
| rs474943 | Clonal hematopoiesis (large clone) | LAS | 20.556 | 26.317 |
| rs4906361 | Clonal hematopoiesis (large clone) | LAS | 22.900 | 26.317 |
| rs633137 | Clonal hematopoiesis (large clone) | LAS | 22.191 | 26.317 |
| rs7207177 | Clonal hematopoiesis (large clone) | LAS | 24.885 | 26.317 |
| rs724952 | Clonal hematopoiesis (large clone) | LAS | 20.633 | 26.317 |
| rs73020212 | Clonal hematopoiesis (large clone) | LAS | 21.809 | 26.317 |
| rs74893169 | Clonal hematopoiesis (large clone) | LAS | 20.997 | 26.317 |
| rs76887998 | Clonal hematopoiesis (large clone) | LAS | 22.655 | 26.317 |
| rs7705526 | Clonal hematopoiesis (large clone) | LAS | 116.907 | 26.317 |
| rs78970714 | Clonal hematopoiesis (large clone) | LAS | 19.756 | 26.317 |
| rs8066979 | Clonal hematopoiesis (large clone) | LAS | 19.742 | 26.317 |
| rs10790722 | Clonal hematopoiesis (small clone) | LAS | 20.009 | 25.508 |
| rs10980423 | Clonal hematopoiesis (small clone) | LAS | 20.006 | 25.508 |
| rs11038147 | Clonal hematopoiesis (small clone) | LAS | 20.688 | 25.508 |
| rs11055736 | Clonal hematopoiesis (small clone) | LAS | 20.185 | 25.508 |
| rs112678842 | Clonal hematopoiesis (small clone) | LAS | 19.972 | 25.508 |
| rs115984969 | Clonal hematopoiesis (small clone) | LAS | 21.811 | 25.508 |
| rs116944293 | Clonal hematopoiesis (small clone) | LAS | 21.110 | 25.508 |
| rs118024410 | Clonal hematopoiesis (small clone) | LAS | 21.141 | 25.508 |
| rs13130545 | Clonal hematopoiesis (small clone) | LAS | 22.341 | 25.508 |
| rs138239047 | Clonal hematopoiesis (small clone) | LAS | 20.360 | 25.508 |
| rs141030142 | Clonal hematopoiesis (small clone) | LAS | 20.089 | 25.508 |
| rs141271281 | Clonal hematopoiesis (small clone) | LAS | 20.126 | 25.508 |
| rs17681776 | Clonal hematopoiesis (small clone) | LAS | 20.711 | 25.508 |
| rs182606956 | Clonal hematopoiesis (small clone) | LAS | 20.747 | 25.508 |
| rs188129997 | Clonal hematopoiesis (small clone) | LAS | 22.095 | 25.508 |
| rs1904398 | Clonal hematopoiesis (small clone) | LAS | 19.701 | 25.508 |
| rs2853677 | Clonal hematopoiesis (small clone) | LAS | 120.370 | 25.508 |
| rs35452836 | Clonal hematopoiesis (small clone) | LAS | 36.219 | 25.508 |
| rs3743840 | Clonal hematopoiesis (small clone) | LAS | 19.830 | 25.508 |
| rs4850108 | Clonal hematopoiesis (small clone) | LAS | 22.042 | 25.508 |
| rs6026564 | Clonal hematopoiesis (small clone) | LAS | 24.644 | 25.508 |
| rs6580637 | Clonal hematopoiesis (small clone) | LAS | 19.785 | 25.508 |
| rs7129527 | Clonal hematopoiesis (small clone) | LAS | 20.589 | 25.508 |
| rs72755524 | Clonal hematopoiesis (small clone) | LAS | 30.114 | 25.508 |
| rs75760715 | Clonal hematopoiesis (small clone) | LAS | 21.586 | 25.508 |
| rs759656 | Clonal hematopoiesis (small clone) | LAS | 24.441 | 25.508 |
| rs7702021 | Clonal hematopoiesis (small clone) | LAS | 21.248 | 25.508 |
| rs79406832 | Clonal hematopoiesis (small clone) | LAS | 22.253 | 25.508 |
| rs1029411 | Clonal hematopoiesis (overall) | CES | 19.809 | 29.126 |
| rs11212666 | Clonal hematopoiesis (overall) | CES | 34.515 | 29.126 |
| rs113892140 | Clonal hematopoiesis (overall) | CES | 20.682 | 29.126 |
| rs115137644 | Clonal hematopoiesis (overall) | CES | 20.720 | 29.126 |
| rs11611479 | Clonal hematopoiesis (overall) | CES | 23.350 | 29.126 |
| rs11695542 | Clonal hematopoiesis (overall) | CES | 20.366 | 29.126 |
| rs117314695 | Clonal hematopoiesis (overall) | CES | 21.544 | 29.126 |
| rs117472123 | Clonal hematopoiesis (overall) | CES | 20.970 | 29.126 |
| rs117485768 | Clonal hematopoiesis (overall) | CES | 20.089 | 29.126 |
| rs118121072 | Clonal hematopoiesis (overall) | CES | 20.306 | 29.126 |
| rs11880163 | Clonal hematopoiesis (overall) | CES | 20.466 | 29.126 |
| rs12632224 | Clonal hematopoiesis (overall) | CES | 35.817 | 29.126 |
| rs12893938 | Clonal hematopoiesis (overall) | CES | 21.603 | 29.126 |
| rs12904882 | Clonal hematopoiesis (overall) | CES | 20.947 | 29.126 |
| rs13130545 | Clonal hematopoiesis (overall) | CES | 29.390 | 29.126 |
| rs138239047 | Clonal hematopoiesis (overall) | CES | 20.519 | 29.126 |
| rs143144207 | Clonal hematopoiesis (overall) | CES | 20.630 | 29.126 |
| rs17696019 | Clonal hematopoiesis (overall) | CES | 20.088 | 29.126 |
| rs2077810 | Clonal hematopoiesis (overall) | CES | 19.806 | 29.126 |
| rs2853677 | Clonal hematopoiesis (overall) | CES | 222.544 | 29.126 |
| rs35452836 | Clonal hematopoiesis (overall) | CES | 34.018 | 29.126 |
| rs45564234 | Clonal hematopoiesis (overall) | CES | 20.533 | 29.126 |
| rs6026564 | Clonal hematopoiesis (overall) | CES | 19.559 | 29.126 |
| rs61392716 | Clonal hematopoiesis (overall) | CES | 21.891 | 29.126 |
| rs62105066 | Clonal hematopoiesis (overall) | CES | 21.475 | 29.126 |
| rs6442623 | Clonal hematopoiesis (overall) | CES | 21.926 | 29.126 |
| rs6841004 | Clonal hematopoiesis (overall) | CES | 20.178 | 29.126 |
| rs7207177 | Clonal hematopoiesis (overall) | CES | 23.482 | 29.126 |
| rs7232918 | Clonal hematopoiesis (overall) | CES | 24.934 | 29.126 |
| rs72755524 | Clonal hematopoiesis (overall) | CES | 19.682 | 29.126 |
| rs759656 | Clonal hematopoiesis (overall) | CES | 29.170 | 29.126 |
| rs80019846 | Clonal hematopoiesis (overall) | CES | 21.010 | 29.126 |
| rs1021797 | Clonal hematopoiesis (DNMT3A mutation) | CES | 25.442 | 31.140 |
| rs10864219 | Clonal hematopoiesis (DNMT3A mutation) | CES | 24.350 | 31.140 |
| rs10942770 | Clonal hematopoiesis (DNMT3A mutation) | CES | 24.857 | 31.140 |
| rs11257356 | Clonal hematopoiesis (DNMT3A mutation) | CES | 20.083 | 31.140 |
| rs112675842 | Clonal hematopoiesis (DNMT3A mutation) | CES | 23.038 | 31.140 |
| rs116911541 | Clonal hematopoiesis (DNMT3A mutation) | CES | 20.479 | 31.140 |
| rs117475117 | Clonal hematopoiesis (DNMT3A mutation) | CES | 21.040 | 31.140 |
| rs11880163 | Clonal hematopoiesis (DNMT3A mutation) | CES | 21.299 | 31.140 |
| rs12524502 | Clonal hematopoiesis (DNMT3A mutation) | CES | 70.969 | 31.140 |
| rs12634120 | Clonal hematopoiesis (DNMT3A mutation) | CES | 25.660 | 31.140 |
| rs12692566 | Clonal hematopoiesis (DNMT3A mutation) | CES | 23.719 | 31.140 |
| rs12821838 | Clonal hematopoiesis (DNMT3A mutation) | CES | 20.261 | 31.140 |
| rs228606 | Clonal hematopoiesis (DNMT3A mutation) | CES | 51.301 | 31.140 |
| rs2296312 | Clonal hematopoiesis (DNMT3A mutation) | CES | 33.044 | 31.140 |
| rs2841648 | Clonal hematopoiesis (DNMT3A mutation) | CES | 26.926 | 31.140 |
| rs2853677 | Clonal hematopoiesis (DNMT3A mutation) | CES | 172.379 | 31.140 |
| rs28689133 | Clonal hematopoiesis (DNMT3A mutation) | CES | 19.517 | 31.140 |
| rs34795680 | Clonal hematopoiesis (DNMT3A mutation) | CES | 22.035 | 31.140 |
| rs4234481 | Clonal hematopoiesis (DNMT3A mutation) | CES | 21.773 | 31.140 |
| rs4662525 | Clonal hematopoiesis (DNMT3A mutation) | CES | 22.079 | 31.140 |
| rs4669004 | Clonal hematopoiesis (DNMT3A mutation) | CES | 19.417 | 31.140 |
| rs4779071 | Clonal hematopoiesis (DNMT3A mutation) | CES | 19.926 | 31.140 |
| rs62237573 | Clonal hematopoiesis (DNMT3A mutation) | CES | 23.774 | 31.140 |
| rs759656 | Clonal hematopoiesis (DNMT3A mutation) | CES | 24.364 | 31.140 |
| rs77481539 | Clonal hematopoiesis (DNMT3A mutation) | CES | 22.486 | 31.140 |
| rs77802645 | Clonal hematopoiesis (DNMT3A mutation) | CES | 19.476 | 31.140 |
| rs8088824 | Clonal hematopoiesis (DNMT3A mutation) | CES | 30.300 | 31.140 |
| rs9649961 | Clonal hematopoiesis (DNMT3A mutation) | CES | 21.921 | 31.140 |
| rs10131341 | Clonal hematopoiesis (TET2 mutation) | CES | 38.148 | 24.407 |
| rs10782071 | Clonal hematopoiesis (TET2 mutation) | CES | 23.276 | 24.407 |
| rs11111098 | Clonal hematopoiesis (TET2 mutation) | CES | 24.004 | 24.407 |
| rs11132125 | Clonal hematopoiesis (TET2 mutation) | CES | 20.724 | 24.407 |
| rs117150600 | Clonal hematopoiesis (TET2 mutation) | CES | 19.995 | 24.407 |
| rs12791065 | Clonal hematopoiesis (TET2 mutation) | CES | 24.724 | 24.407 |
| rs12890144 | Clonal hematopoiesis (TET2 mutation) | CES | 19.644 | 24.407 |
| rs189506759 | Clonal hematopoiesis (TET2 mutation) | CES | 19.633 | 24.407 |
| rs2736100 | Clonal hematopoiesis (TET2 mutation) | CES | 71.774 | 24.407 |
| rs28664269 | Clonal hematopoiesis (TET2 mutation) | CES | 19.792 | 24.407 |
| rs35484216 | Clonal hematopoiesis (TET2 mutation) | CES | 22.178 | 24.407 |
| rs41272399 | Clonal hematopoiesis (TET2 mutation) | CES | 19.534 | 24.407 |
| rs4145796 | Clonal hematopoiesis (TET2 mutation) | CES | 20.130 | 24.407 |
| rs56327306 | Clonal hematopoiesis (TET2 mutation) | CES | 21.175 | 24.407 |
| rs57019067 | Clonal hematopoiesis (TET2 mutation) | CES | 20.911 | 24.407 |
| rs59108483 | Clonal hematopoiesis (TET2 mutation) | CES | 19.871 | 24.407 |
| rs61951330 | Clonal hematopoiesis (TET2 mutation) | CES | 19.993 | 24.407 |
| rs73214874 | Clonal hematopoiesis (TET2 mutation) | CES | 20.799 | 24.407 |
| rs7739373 | Clonal hematopoiesis (TET2 mutation) | CES | 20.196 | 24.407 |
| rs7816774 | Clonal hematopoiesis (TET2 mutation) | CES | 20.991 | 24.407 |
| rs78729418 | Clonal hematopoiesis (TET2 mutation) | CES | 20.553 | 24.407 |
| rs78849954 | Clonal hematopoiesis (TET2 mutation) | CES | 19.560 | 24.407 |
| rs79020059 | Clonal hematopoiesis (TET2 mutation) | CES | 22.504 | 24.407 |
| rs79633204 | Clonal hematopoiesis (TET2 mutation) | CES | 37.592 | 24.407 |
| rs858339 | Clonal hematopoiesis (TET2 mutation) | CES | 22.478 | 24.407 |
| rs112836922 | Clonal hematopoiesis (large clone) | CES | 20.772 | 26.317 |
| rs117066841 | Clonal hematopoiesis (large clone) | CES | 19.860 | 26.317 |
| rs117942522 | Clonal hematopoiesis (large clone) | CES | 21.291 | 26.317 |
| rs12615707 | Clonal hematopoiesis (large clone) | CES | 21.510 | 26.317 |
| rs12632224 | Clonal hematopoiesis (large clone) | CES | 29.101 | 26.317 |
| rs140599943 | Clonal hematopoiesis (large clone) | CES | 22.898 | 26.317 |
| rs144815420 | Clonal hematopoiesis (large clone) | CES | 21.173 | 26.317 |
| rs146838695 | Clonal hematopoiesis (large clone) | CES | 19.983 | 26.317 |
| rs148557733 | Clonal hematopoiesis (large clone) | CES | 20.368 | 26.317 |
| rs150846798 | Clonal hematopoiesis (large clone) | CES | 25.945 | 26.317 |
| rs2468948 | Clonal hematopoiesis (large clone) | CES | 23.046 | 26.317 |
| rs474943 | Clonal hematopoiesis (large clone) | CES | 20.556 | 26.317 |
| rs4906361 | Clonal hematopoiesis (large clone) | CES | 22.900 | 26.317 |
| rs633137 | Clonal hematopoiesis (large clone) | CES | 22.191 | 26.317 |
| rs7207177 | Clonal hematopoiesis (large clone) | CES | 24.885 | 26.317 |
| rs724952 | Clonal hematopoiesis (large clone) | CES | 20.633 | 26.317 |
| rs73020212 | Clonal hematopoiesis (large clone) | CES | 21.809 | 26.317 |
| rs74893169 | Clonal hematopoiesis (large clone) | CES | 20.997 | 26.317 |
| rs76887998 | Clonal hematopoiesis (large clone) | CES | 22.655 | 26.317 |
| rs7705526 | Clonal hematopoiesis (large clone) | CES | 116.907 | 26.317 |
| rs78970714 | Clonal hematopoiesis (large clone) | CES | 19.756 | 26.317 |
| rs8066979 | Clonal hematopoiesis (large clone) | CES | 19.742 | 26.317 |
| rs10790722 | Clonal hematopoiesis (small clone) | CES | 20.009 | 25.508 |
| rs10980423 | Clonal hematopoiesis (small clone) | CES | 20.006 | 25.508 |
| rs11038147 | Clonal hematopoiesis (small clone) | CES | 20.688 | 25.508 |
| rs11055736 | Clonal hematopoiesis (small clone) | CES | 20.185 | 25.508 |
| rs112678842 | Clonal hematopoiesis (small clone) | CES | 19.972 | 25.508 |
| rs115984969 | Clonal hematopoiesis (small clone) | CES | 21.811 | 25.508 |
| rs116944293 | Clonal hematopoiesis (small clone) | CES | 21.110 | 25.508 |
| rs118024410 | Clonal hematopoiesis (small clone) | CES | 21.141 | 25.508 |
| rs13130545 | Clonal hematopoiesis (small clone) | CES | 22.341 | 25.508 |
| rs138239047 | Clonal hematopoiesis (small clone) | CES | 20.360 | 25.508 |
| rs141030142 | Clonal hematopoiesis (small clone) | CES | 20.089 | 25.508 |
| rs141271281 | Clonal hematopoiesis (small clone) | CES | 20.126 | 25.508 |
| rs17681776 | Clonal hematopoiesis (small clone) | CES | 20.711 | 25.508 |
| rs182606956 | Clonal hematopoiesis (small clone) | CES | 20.747 | 25.508 |
| rs188129997 | Clonal hematopoiesis (small clone) | CES | 22.095 | 25.508 |
| rs1904398 | Clonal hematopoiesis (small clone) | CES | 19.701 | 25.508 |
| rs2853677 | Clonal hematopoiesis (small clone) | CES | 120.370 | 25.508 |
| rs35452836 | Clonal hematopoiesis (small clone) | CES | 36.219 | 25.508 |
| rs3743840 | Clonal hematopoiesis (small clone) | CES | 19.830 | 25.508 |
| rs4850108 | Clonal hematopoiesis (small clone) | CES | 22.042 | 25.508 |
| rs6026564 | Clonal hematopoiesis (small clone) | CES | 24.644 | 25.508 |
| rs6580637 | Clonal hematopoiesis (small clone) | CES | 19.785 | 25.508 |
| rs7129527 | Clonal hematopoiesis (small clone) | CES | 20.589 | 25.508 |
| rs72755524 | Clonal hematopoiesis (small clone) | CES | 30.114 | 25.508 |
| rs75760715 | Clonal hematopoiesis (small clone) | CES | 21.586 | 25.508 |
| rs759656 | Clonal hematopoiesis (small clone) | CES | 24.441 | 25.508 |
| rs7702021 | Clonal hematopoiesis (small clone) | CES | 21.248 | 25.508 |
| rs79406832 | Clonal hematopoiesis (small clone) | CES | 22.253 | 25.508 |
| rs1029411 | Clonal hematopoiesis (overall) | SVS | 19.809 | 29.126 |
| rs11212666 | Clonal hematopoiesis (overall) | SVS | 34.515 | 29.126 |
| rs113892140 | Clonal hematopoiesis (overall) | SVS | 20.682 | 29.126 |
| rs115137644 | Clonal hematopoiesis (overall) | SVS | 20.720 | 29.126 |
| rs11611479 | Clonal hematopoiesis (overall) | SVS | 23.350 | 29.126 |
| rs11695542 | Clonal hematopoiesis (overall) | SVS | 20.366 | 29.126 |
| rs117314695 | Clonal hematopoiesis (overall) | SVS | 21.544 | 29.126 |
| rs117472123 | Clonal hematopoiesis (overall) | SVS | 20.970 | 29.126 |
| rs117485768 | Clonal hematopoiesis (overall) | SVS | 20.089 | 29.126 |
| rs118121072 | Clonal hematopoiesis (overall) | SVS | 20.306 | 29.126 |
| rs11880163 | Clonal hematopoiesis (overall) | SVS | 20.466 | 29.126 |
| rs12632224 | Clonal hematopoiesis (overall) | SVS | 35.817 | 29.126 |
| rs12893938 | Clonal hematopoiesis (overall) | SVS | 21.603 | 29.126 |
| rs12904882 | Clonal hematopoiesis (overall) | SVS | 20.947 | 29.126 |
| rs13130545 | Clonal hematopoiesis (overall) | SVS | 29.390 | 29.126 |
| rs138239047 | Clonal hematopoiesis (overall) | SVS | 20.519 | 29.126 |
| rs143144207 | Clonal hematopoiesis (overall) | SVS | 20.630 | 29.126 |
| rs17696019 | Clonal hematopoiesis (overall) | SVS | 20.088 | 29.126 |
| rs2077810 | Clonal hematopoiesis (overall) | SVS | 19.806 | 29.126 |
| rs2853677 | Clonal hematopoiesis (overall) | SVS | 222.544 | 29.126 |
| rs35452836 | Clonal hematopoiesis (overall) | SVS | 34.018 | 29.126 |
| rs45564234 | Clonal hematopoiesis (overall) | SVS | 20.533 | 29.126 |
| rs6026564 | Clonal hematopoiesis (overall) | SVS | 19.559 | 29.126 |
| rs61392716 | Clonal hematopoiesis (overall) | SVS | 21.891 | 29.126 |
| rs62105066 | Clonal hematopoiesis (overall) | SVS | 21.475 | 29.126 |
| rs6442623 | Clonal hematopoiesis (overall) | SVS | 21.926 | 29.126 |
| rs6841004 | Clonal hematopoiesis (overall) | SVS | 20.178 | 29.126 |
| rs7207177 | Clonal hematopoiesis (overall) | SVS | 23.482 | 29.126 |
| rs7232918 | Clonal hematopoiesis (overall) | SVS | 24.934 | 29.126 |
| rs72755524 | Clonal hematopoiesis (overall) | SVS | 19.682 | 29.126 |
| rs759656 | Clonal hematopoiesis (overall) | SVS | 29.170 | 29.126 |
| rs80019846 | Clonal hematopoiesis (overall) | SVS | 21.010 | 29.126 |
| rs1021797 | Clonal hematopoiesis (DNMT3A mutation) | SVS | 25.442 | 31.140 |
| rs10864219 | Clonal hematopoiesis (DNMT3A mutation) | SVS | 24.350 | 31.140 |
| rs10942770 | Clonal hematopoiesis (DNMT3A mutation) | SVS | 24.857 | 31.140 |
| rs11257356 | Clonal hematopoiesis (DNMT3A mutation) | SVS | 20.083 | 31.140 |
| rs112675842 | Clonal hematopoiesis (DNMT3A mutation) | SVS | 23.038 | 31.140 |
| rs116911541 | Clonal hematopoiesis (DNMT3A mutation) | SVS | 20.479 | 31.140 |
| rs117475117 | Clonal hematopoiesis (DNMT3A mutation) | SVS | 21.040 | 31.140 |
| rs11880163 | Clonal hematopoiesis (DNMT3A mutation) | SVS | 21.299 | 31.140 |
| rs12524502 | Clonal hematopoiesis (DNMT3A mutation) | SVS | 70.969 | 31.140 |
| rs12634120 | Clonal hematopoiesis (DNMT3A mutation) | SVS | 25.660 | 31.140 |
| rs12692566 | Clonal hematopoiesis (DNMT3A mutation) | SVS | 23.719 | 31.140 |
| rs12821838 | Clonal hematopoiesis (DNMT3A mutation) | SVS | 20.261 | 31.140 |
| rs228606 | Clonal hematopoiesis (DNMT3A mutation) | SVS | 51.301 | 31.140 |
| rs2296312 | Clonal hematopoiesis (DNMT3A mutation) | SVS | 33.044 | 31.140 |
| rs2841648 | Clonal hematopoiesis (DNMT3A mutation) | SVS | 26.926 | 31.140 |
| rs2853677 | Clonal hematopoiesis (DNMT3A mutation) | SVS | 172.379 | 31.140 |
| rs28689133 | Clonal hematopoiesis (DNMT3A mutation) | SVS | 19.517 | 31.140 |
| rs34795680 | Clonal hematopoiesis (DNMT3A mutation) | SVS | 22.035 | 31.140 |
| rs4234481 | Clonal hematopoiesis (DNMT3A mutation) | SVS | 21.773 | 31.140 |
| rs4662525 | Clonal hematopoiesis (DNMT3A mutation) | SVS | 22.079 | 31.140 |
| rs4669004 | Clonal hematopoiesis (DNMT3A mutation) | SVS | 19.417 | 31.140 |
| rs4779071 | Clonal hematopoiesis (DNMT3A mutation) | SVS | 19.926 | 31.140 |
| rs62237573 | Clonal hematopoiesis (DNMT3A mutation) | SVS | 23.774 | 31.140 |
| rs759656 | Clonal hematopoiesis (DNMT3A mutation) | SVS | 24.364 | 31.140 |
| rs77481539 | Clonal hematopoiesis (DNMT3A mutation) | SVS | 22.486 | 31.140 |
| rs77802645 | Clonal hematopoiesis (DNMT3A mutation) | SVS | 19.476 | 31.140 |
| rs8088824 | Clonal hematopoiesis (DNMT3A mutation) | SVS | 30.300 | 31.140 |
| rs9649961 | Clonal hematopoiesis (DNMT3A mutation) | SVS | 21.921 | 31.140 |
| rs10131341 | Clonal hematopoiesis (TET2 mutation) | SVS | 38.148 | 24.407 |
| rs10782071 | Clonal hematopoiesis (TET2 mutation) | SVS | 23.276 | 24.407 |
| rs11111098 | Clonal hematopoiesis (TET2 mutation) | SVS | 24.004 | 24.407 |
| rs11132125 | Clonal hematopoiesis (TET2 mutation) | SVS | 20.724 | 24.407 |
| rs117150600 | Clonal hematopoiesis (TET2 mutation) | SVS | 19.995 | 24.407 |
| rs12791065 | Clonal hematopoiesis (TET2 mutation) | SVS | 24.724 | 24.407 |
| rs12890144 | Clonal hematopoiesis (TET2 mutation) | SVS | 19.644 | 24.407 |
| rs189506759 | Clonal hematopoiesis (TET2 mutation) | SVS | 19.633 | 24.407 |
| rs2736100 | Clonal hematopoiesis (TET2 mutation) | SVS | 71.774 | 24.407 |
| rs28664269 | Clonal hematopoiesis (TET2 mutation) | SVS | 19.792 | 24.407 |
| rs35484216 | Clonal hematopoiesis (TET2 mutation) | SVS | 22.178 | 24.407 |
| rs41272399 | Clonal hematopoiesis (TET2 mutation) | SVS | 19.534 | 24.407 |
| rs4145796 | Clonal hematopoiesis (TET2 mutation) | SVS | 20.130 | 24.407 |
| rs56327306 | Clonal hematopoiesis (TET2 mutation) | SVS | 21.175 | 24.407 |
| rs57019067 | Clonal hematopoiesis (TET2 mutation) | SVS | 20.911 | 24.407 |
| rs59108483 | Clonal hematopoiesis (TET2 mutation) | SVS | 19.871 | 24.407 |
| rs61951330 | Clonal hematopoiesis (TET2 mutation) | SVS | 19.993 | 24.407 |
| rs73214874 | Clonal hematopoiesis (TET2 mutation) | SVS | 20.799 | 24.407 |
| rs7739373 | Clonal hematopoiesis (TET2 mutation) | SVS | 20.196 | 24.407 |
| rs7816774 | Clonal hematopoiesis (TET2 mutation) | SVS | 20.991 | 24.407 |
| rs78729418 | Clonal hematopoiesis (TET2 mutation) | SVS | 20.553 | 24.407 |
| rs78849954 | Clonal hematopoiesis (TET2 mutation) | SVS | 19.560 | 24.407 |
| rs79020059 | Clonal hematopoiesis (TET2 mutation) | SVS | 22.504 | 24.407 |
| rs79633204 | Clonal hematopoiesis (TET2 mutation) | SVS | 37.592 | 24.407 |
| rs858339 | Clonal hematopoiesis (TET2 mutation) | SVS | 22.478 | 24.407 |
| rs112836922 | Clonal hematopoiesis (large clone) | SVS | 20.772 | 26.317 |
| rs117066841 | Clonal hematopoiesis (large clone) | SVS | 19.860 | 26.317 |
| rs117942522 | Clonal hematopoiesis (large clone) | SVS | 21.291 | 26.317 |
| rs12615707 | Clonal hematopoiesis (large clone) | SVS | 21.510 | 26.317 |
| rs12632224 | Clonal hematopoiesis (large clone) | SVS | 29.101 | 26.317 |
| rs140599943 | Clonal hematopoiesis (large clone) | SVS | 22.898 | 26.317 |
| rs144815420 | Clonal hematopoiesis (large clone) | SVS | 21.173 | 26.317 |
| rs146838695 | Clonal hematopoiesis (large clone) | SVS | 19.983 | 26.317 |
| rs148557733 | Clonal hematopoiesis (large clone) | SVS | 20.368 | 26.317 |
| rs150846798 | Clonal hematopoiesis (large clone) | SVS | 25.945 | 26.317 |
| rs2468948 | Clonal hematopoiesis (large clone) | SVS | 23.046 | 26.317 |
| rs474943 | Clonal hematopoiesis (large clone) | SVS | 20.556 | 26.317 |
| rs4906361 | Clonal hematopoiesis (large clone) | SVS | 22.900 | 26.317 |
| rs633137 | Clonal hematopoiesis (large clone) | SVS | 22.191 | 26.317 |
| rs7207177 | Clonal hematopoiesis (large clone) | SVS | 24.885 | 26.317 |
| rs724952 | Clonal hematopoiesis (large clone) | SVS | 20.633 | 26.317 |
| rs73020212 | Clonal hematopoiesis (large clone) | SVS | 21.809 | 26.317 |
| rs74893169 | Clonal hematopoiesis (large clone) | SVS | 20.997 | 26.317 |
| rs76887998 | Clonal hematopoiesis (large clone) | SVS | 22.655 | 26.317 |
| rs7705526 | Clonal hematopoiesis (large clone) | SVS | 116.907 | 26.317 |
| rs78970714 | Clonal hematopoiesis (large clone) | SVS | 19.756 | 26.317 |
| rs8066979 | Clonal hematopoiesis (large clone) | SVS | 19.742 | 26.317 |
| rs10790722 | Clonal hematopoiesis (small clone) | SVS | 20.009 | 25.508 |
| rs10980423 | Clonal hematopoiesis (small clone) | SVS | 20.006 | 25.508 |
| rs11038147 | Clonal hematopoiesis (small clone) | SVS | 20.688 | 25.508 |
| rs11055736 | Clonal hematopoiesis (small clone) | SVS | 20.185 | 25.508 |
| rs112678842 | Clonal hematopoiesis (small clone) | SVS | 19.972 | 25.508 |
| rs115984969 | Clonal hematopoiesis (small clone) | SVS | 21.811 | 25.508 |
| rs116944293 | Clonal hematopoiesis (small clone) | SVS | 21.110 | 25.508 |
| rs118024410 | Clonal hematopoiesis (small clone) | SVS | 21.141 | 25.508 |
| rs13130545 | Clonal hematopoiesis (small clone) | SVS | 22.341 | 25.508 |
| rs138239047 | Clonal hematopoiesis (small clone) | SVS | 20.360 | 25.508 |
| rs141030142 | Clonal hematopoiesis (small clone) | SVS | 20.089 | 25.508 |
| rs141271281 | Clonal hematopoiesis (small clone) | SVS | 20.126 | 25.508 |
| rs17681776 | Clonal hematopoiesis (small clone) | SVS | 20.711 | 25.508 |
| rs182606956 | Clonal hematopoiesis (small clone) | SVS | 20.747 | 25.508 |
| rs188129997 | Clonal hematopoiesis (small clone) | SVS | 22.095 | 25.508 |
| rs1904398 | Clonal hematopoiesis (small clone) | SVS | 19.701 | 25.508 |
| rs2853677 | Clonal hematopoiesis (small clone) | SVS | 120.370 | 25.508 |
| rs35452836 | Clonal hematopoiesis (small clone) | SVS | 36.219 | 25.508 |
| rs3743840 | Clonal hematopoiesis (small clone) | SVS | 19.830 | 25.508 |
| rs4850108 | Clonal hematopoiesis (small clone) | SVS | 22.042 | 25.508 |
| rs6026564 | Clonal hematopoiesis (small clone) | SVS | 24.644 | 25.508 |
| rs6580637 | Clonal hematopoiesis (small clone) | SVS | 19.785 | 25.508 |
| rs7129527 | Clonal hematopoiesis (small clone) | SVS | 20.589 | 25.508 |
| rs72755524 | Clonal hematopoiesis (small clone) | SVS | 30.114 | 25.508 |
| rs75760715 | Clonal hematopoiesis (small clone) | SVS | 21.586 | 25.508 |
| rs759656 | Clonal hematopoiesis (small clone) | SVS | 24.441 | 25.508 |
| rs7702021 | Clonal hematopoiesis (small clone) | SVS | 21.248 | 25.508 |
| rs79406832 | Clonal hematopoiesis (small clone) | SVS | 22.253 | 25.508 |
| rs1029411 | Clonal hematopoiesis (overall) | Prognosis | 19.809 | 30.620 |
| rs11212666 | Clonal hematopoiesis (overall) | Prognosis | 34.515 | 30.620 |
| rs113892140 | Clonal hematopoiesis (overall) | Prognosis | 20.682 | 30.620 |
| rs115137644 | Clonal hematopoiesis (overall) | Prognosis | 20.720 | 30.620 |
| rs11611479 | Clonal hematopoiesis (overall) | Prognosis | 23.350 | 30.620 |
| rs11695542 | Clonal hematopoiesis (overall) | Prognosis | 20.366 | 30.620 |
| rs117314695 | Clonal hematopoiesis (overall) | Prognosis | 21.544 | 30.620 |
| rs117472123 | Clonal hematopoiesis (overall) | Prognosis | 20.970 | 30.620 |
| rs118121072 | Clonal hematopoiesis (overall) | Prognosis | 20.306 | 30.620 |
| rs12632224 | Clonal hematopoiesis (overall) | Prognosis | 35.817 | 30.620 |
| rs12893938 | Clonal hematopoiesis (overall) | Prognosis | 21.603 | 30.620 |
| rs12904882 | Clonal hematopoiesis (overall) | Prognosis | 20.947 | 30.620 |
| rs13130545 | Clonal hematopoiesis (overall) | Prognosis | 29.390 | 30.620 |
| rs138239047 | Clonal hematopoiesis (overall) | Prognosis | 20.519 | 30.620 |
| rs17696019 | Clonal hematopoiesis (overall) | Prognosis | 20.088 | 30.620 |
| rs2077810 | Clonal hematopoiesis (overall) | Prognosis | 19.806 | 30.620 |
| rs2853677 | Clonal hematopoiesis (overall) | Prognosis | 222.544 | 30.620 |
| rs45564234 | Clonal hematopoiesis (overall) | Prognosis | 20.533 | 30.620 |
| rs6026564 | Clonal hematopoiesis (overall) | Prognosis | 19.559 | 30.620 |
| rs61392716 | Clonal hematopoiesis (overall) | Prognosis | 21.891 | 30.620 |
| rs62105066 | Clonal hematopoiesis (overall) | Prognosis | 21.475 | 30.620 |
| rs6442623 | Clonal hematopoiesis (overall) | Prognosis | 21.926 | 30.620 |
| rs6841004 | Clonal hematopoiesis (overall) | Prognosis | 20.178 | 30.620 |
| rs7207177 | Clonal hematopoiesis (overall) | Prognosis | 23.482 | 30.620 |
| rs7232918 | Clonal hematopoiesis (overall) | Prognosis | 24.934 | 30.620 |
| rs759656 | Clonal hematopoiesis (overall) | Prognosis | 29.170 | 30.620 |
| rs1021797 | Clonal hematopoiesis (DNMT3A mutation) | Prognosis | 25.442 | 32.174 |
| rs10864219 | Clonal hematopoiesis (DNMT3A mutation) | Prognosis | 24.350 | 32.174 |
| rs10942770 | Clonal hematopoiesis (DNMT3A mutation) | Prognosis | 24.857 | 32.174 |
| rs11257356 | Clonal hematopoiesis (DNMT3A mutation) | Prognosis | 20.083 | 32.174 |
| rs112675842 | Clonal hematopoiesis (DNMT3A mutation) | Prognosis | 23.038 | 32.174 |
| rs116911541 | Clonal hematopoiesis (DNMT3A mutation) | Prognosis | 20.479 | 32.174 |
| rs117475117 | Clonal hematopoiesis (DNMT3A mutation) | Prognosis | 21.040 | 32.174 |
| rs12524502 | Clonal hematopoiesis (DNMT3A mutation) | Prognosis | 70.969 | 32.174 |
| rs12634120 | Clonal hematopoiesis (DNMT3A mutation) | Prognosis | 25.660 | 32.174 |
| rs12692566 | Clonal hematopoiesis (DNMT3A mutation) | Prognosis | 23.719 | 32.174 |
| rs12821838 | Clonal hematopoiesis (DNMT3A mutation) | Prognosis | 20.261 | 32.174 |
| rs228606 | Clonal hematopoiesis (DNMT3A mutation) | Prognosis | 51.301 | 32.174 |
| rs2296312 | Clonal hematopoiesis (DNMT3A mutation) | Prognosis | 33.044 | 32.174 |
| rs2841648 | Clonal hematopoiesis (DNMT3A mutation) | Prognosis | 26.926 | 32.174 |
| rs2853677 | Clonal hematopoiesis (DNMT3A mutation) | Prognosis | 172.379 | 32.174 |
| rs28689133 | Clonal hematopoiesis (DNMT3A mutation) | Prognosis | 19.517 | 32.174 |
| rs34795680 | Clonal hematopoiesis (DNMT3A mutation) | Prognosis | 22.035 | 32.174 |
| rs4234481 | Clonal hematopoiesis (DNMT3A mutation) | Prognosis | 21.773 | 32.174 |
| rs4662525 | Clonal hematopoiesis (DNMT3A mutation) | Prognosis | 22.079 | 32.174 |
| rs4669004 | Clonal hematopoiesis (DNMT3A mutation) | Prognosis | 19.417 | 32.174 |
| rs4779071 | Clonal hematopoiesis (DNMT3A mutation) | Prognosis | 19.926 | 32.174 |
| rs759656 | Clonal hematopoiesis (DNMT3A mutation) | Prognosis | 24.364 | 32.174 |
| rs77802645 | Clonal hematopoiesis (DNMT3A mutation) | Prognosis | 19.476 | 32.174 |
| rs8088824 | Clonal hematopoiesis (DNMT3A mutation) | Prognosis | 30.300 | 32.174 |
| rs9649961 | Clonal hematopoiesis (DNMT3A mutation) | Prognosis | 21.921 | 32.174 |
| rs10131341 | Clonal hematopoiesis (TET2 mutation) | Prognosis | 38.148 | 24.304 |
| rs10782071 | Clonal hematopoiesis (TET2 mutation) | Prognosis | 23.276 | 24.304 |
| rs11111098 | Clonal hematopoiesis (TET2 mutation) | Prognosis | 24.004 | 24.304 |
| rs11132125 | Clonal hematopoiesis (TET2 mutation) | Prognosis | 20.724 | 24.304 |
| rs117150600 | Clonal hematopoiesis (TET2 mutation) | Prognosis | 19.995 | 24.304 |
| rs12791065 | Clonal hematopoiesis (TET2 mutation) | Prognosis | 24.724 | 24.304 |
| rs12890144 | Clonal hematopoiesis (TET2 mutation) | Prognosis | 19.644 | 24.304 |
| rs189506759 | Clonal hematopoiesis (TET2 mutation) | Prognosis | 19.633 | 24.304 |
| rs2736100 | Clonal hematopoiesis (TET2 mutation) | Prognosis | 71.774 | 24.304 |
| rs28664269 | Clonal hematopoiesis (TET2 mutation) | Prognosis | 19.792 | 24.304 |
| rs35484216 | Clonal hematopoiesis (TET2 mutation) | Prognosis | 22.178 | 24.304 |
| rs369451404 | Clonal hematopoiesis (TET2 mutation) | Prognosis | 24.183 | 24.304 |
| rs41272399 | Clonal hematopoiesis (TET2 mutation) | Prognosis | 19.534 | 24.304 |
| rs4145796 | Clonal hematopoiesis (TET2 mutation) | Prognosis | 20.130 | 24.304 |
| rs56327306 | Clonal hematopoiesis (TET2 mutation) | Prognosis | 21.175 | 24.304 |
| rs57019067 | Clonal hematopoiesis (TET2 mutation) | Prognosis | 20.911 | 24.304 |
| rs59108483 | Clonal hematopoiesis (TET2 mutation) | Prognosis | 19.871 | 24.304 |
| rs61951330 | Clonal hematopoiesis (TET2 mutation) | Prognosis | 19.993 | 24.304 |
| rs73214874 | Clonal hematopoiesis (TET2 mutation) | Prognosis | 20.799 | 24.304 |
| rs7739373 | Clonal hematopoiesis (TET2 mutation) | Prognosis | 20.196 | 24.304 |
| rs7816774 | Clonal hematopoiesis (TET2 mutation) | Prognosis | 20.991 | 24.304 |
| rs78729418 | Clonal hematopoiesis (TET2 mutation) | Prognosis | 20.553 | 24.304 |
| rs78849954 | Clonal hematopoiesis (TET2 mutation) | Prognosis | 19.560 | 24.304 |
| rs79633204 | Clonal hematopoiesis (TET2 mutation) | Prognosis | 37.592 | 24.304 |
| rs858339 | Clonal hematopoiesis (TET2 mutation) | Prognosis | 22.478 | 24.304 |
| rs9834842 | Clonal hematopoiesis (TET2 mutation) | Prognosis | 20.051 | 24.304 |
| rs112836922 | Clonal hematopoiesis (large clone) | Prognosis | 20.772 | 26.458 |
| rs117066841 | Clonal hematopoiesis (large clone) | Prognosis | 19.860 | 26.458 |
| rs117942522 | Clonal hematopoiesis (large clone) | Prognosis | 21.291 | 26.458 |
| rs12615707 | Clonal hematopoiesis (large clone) | Prognosis | 21.510 | 26.458 |
| rs12632224 | Clonal hematopoiesis (large clone) | Prognosis | 29.101 | 26.458 |
| rs140599943 | Clonal hematopoiesis (large clone) | Prognosis | 22.898 | 26.458 |
| rs144815420 | Clonal hematopoiesis (large clone) | Prognosis | 21.173 | 26.458 |
| rs146838695 | Clonal hematopoiesis (large clone) | Prognosis | 19.983 | 26.458 |
| rs150846798 | Clonal hematopoiesis (large clone) | Prognosis | 25.945 | 26.458 |
| rs200513511 | Clonal hematopoiesis (large clone) | Prognosis | 20.629 | 26.458 |
| rs2468948 | Clonal hematopoiesis (large clone) | Prognosis | 23.046 | 26.458 |
| rs474943 | Clonal hematopoiesis (large clone) | Prognosis | 20.556 | 26.458 |
| rs4906361 | Clonal hematopoiesis (large clone) | Prognosis | 22.900 | 26.458 |
| rs633137 | Clonal hematopoiesis (large clone) | Prognosis | 22.191 | 26.458 |
| rs7207177 | Clonal hematopoiesis (large clone) | Prognosis | 24.885 | 26.458 |
| rs724952 | Clonal hematopoiesis (large clone) | Prognosis | 20.633 | 26.458 |
| rs74893169 | Clonal hematopoiesis (large clone) | Prognosis | 20.997 | 26.458 |
| rs76887998 | Clonal hematopoiesis (large clone) | Prognosis | 22.655 | 26.458 |
| rs7705526 | Clonal hematopoiesis (large clone) | Prognosis | 116.907 | 26.458 |
| rs78970714 | Clonal hematopoiesis (large clone) | Prognosis | 19.756 | 26.458 |
| rs8066979 | Clonal hematopoiesis (large clone) | Prognosis | 19.742 | 26.458 |
| rs9900539 | Clonal hematopoiesis (large clone) | Prognosis | 24.643 | 26.458 |
| rs10790722 | Clonal hematopoiesis (small clone) | Prognosis | 20.009 | 25.055 |
| rs10980423 | Clonal hematopoiesis (small clone) | Prognosis | 20.006 | 25.055 |
| rs11038147 | Clonal hematopoiesis (small clone) | Prognosis | 20.688 | 25.055 |
| rs11055736 | Clonal hematopoiesis (small clone) | Prognosis | 20.185 | 25.055 |
| rs112678842 | Clonal hematopoiesis (small clone) | Prognosis | 19.972 | 25.055 |
| rs116944293 | Clonal hematopoiesis (small clone) | Prognosis | 21.110 | 25.055 |
| rs118024410 | Clonal hematopoiesis (small clone) | Prognosis | 21.141 | 25.055 |
| rs13130545 | Clonal hematopoiesis (small clone) | Prognosis | 22.341 | 25.055 |
| rs138239047 | Clonal hematopoiesis (small clone) | Prognosis | 20.360 | 25.055 |
| rs141030142 | Clonal hematopoiesis (small clone) | Prognosis | 20.089 | 25.055 |
| rs17681776 | Clonal hematopoiesis (small clone) | Prognosis | 20.711 | 25.055 |
| rs182606956 | Clonal hematopoiesis (small clone) | Prognosis | 20.747 | 25.055 |
| rs188129997 | Clonal hematopoiesis (small clone) | Prognosis | 22.095 | 25.055 |
| rs1904398 | Clonal hematopoiesis (small clone) | Prognosis | 19.701 | 25.055 |
| rs2853677 | Clonal hematopoiesis (small clone) | Prognosis | 120.370 | 25.055 |
| rs3743840 | Clonal hematopoiesis (small clone) | Prognosis | 19.830 | 25.055 |
| rs4850108 | Clonal hematopoiesis (small clone) | Prognosis | 22.042 | 25.055 |
| rs6026564 | Clonal hematopoiesis (small clone) | Prognosis | 24.644 | 25.055 |
| rs61892874 | Clonal hematopoiesis (small clone) | Prognosis | 20.433 | 25.055 |
| rs6580637 | Clonal hematopoiesis (small clone) | Prognosis | 19.785 | 25.055 |
| rs7129527 | Clonal hematopoiesis (small clone) | Prognosis | 20.589 | 25.055 |
| rs75760715 | Clonal hematopoiesis (small clone) | Prognosis | 21.586 | 25.055 |
| rs759656 | Clonal hematopoiesis (small clone) | Prognosis | 24.441 | 25.055 |
| rs7702021 | Clonal hematopoiesis (small clone) | Prognosis | 21.248 | 25.055 |
| rs79406832 | Clonal hematopoiesis (small clone) | Prognosis | 22.253 | 25.055 |

**Table S2** | MR-Egger regression outputs (intercept, p-values).

| **Exposure** | **Output** | **Egger intercept** | **Standard error** | **P_value** |
| --- | --- | --- | --- | --- |
| Clonal hematopoiesis (overall) | AIS | 0.011 | 0.008 | 0.146 |
| Clonal hematopoiesis (DNMT3A mutation) | AIS | 0.014 | 0.009 | 0.112 |
| Clonal hematopoiesis (TET2 mutation) | AIS | -0.015 | 0.009 | 0.104 |
| Clonal hematopoiesis (large clone) | AIS | 0.005 | 0.011 | 0.673 |
| Clonal hematopoiesis (small clone) | AIS | -0.009 | 0.007 | 0.178 |
| Clonal hematopoiesis (overall) | LAS | 0.025 | 0.020 | 0.225 |
| Clonal hematopoiesis (DNMT3A mutation) | LAS | 0.013 | 0.021 | 0.547 |
| Clonal hematopoiesis (TET2 mutation) | LAS | -0.041 | 0.023 | 0.086 |
| Clonal hematopoiesis (large clone) | LAS | -0.028 | 0.030 | 0.360 |
| Clonal hematopoiesis (small clone) | LAS | 0.001 | 0.016 | 0.967 |
| Clonal hematopoiesis (overall) | CES | -0.009 | 0.019 | 0.617 |
| Clonal hematopoiesis (DNMT3A mutation) | CES | -0.012 | 0.014 | 0.397 |
| Clonal hematopoiesis (TET2 mutation) | CES | -0.049 | 0.021 | 0.028 |
| Clonal hematopoiesis (large clone) | CES | 0.009 | 0.022 | 0.684 |
| Clonal hematopoiesis (small clone) | CES | -0.026 | 0.015 | 0.085 |
| Clonal hematopoiesis (overall) | SVS | 0.010 | 0.018 | 0.595 |
| Clonal hematopoiesis (DNMT3A mutation) | SVS | 0.011 | 0.020 | 0.587 |
| Clonal hematopoiesis (TET2 mutation) | SVS | -0.012 | 0.021 | 0.590 |
| Clonal hematopoiesis (large clone) | SVS | -0.041 | 0.027 | 0.142 |
| Clonal hematopoiesis (small clone) | SVS | -0.009 | 0.021 | 0.671 |
| Clonal hematopoiesis (overall) | Prognosis | 0.034 | 0.037 | 0.368 |
| Clonal hematopoiesis (DNMT3A mutation) | Prognosis | 0.076 | 0.036 | 0.046 |
| Clonal hematopoiesis (TET2 mutation) | Prognosis | -0.078 | 0.043 | 0.083 |
| Clonal hematopoiesis (large clone) | Prognosis | 0.022 | 0.020 | 0.265 |
| Clonal hematopoiesis (small clone) | Prognosis | 0.019 | 0.030 | 0.529 |

**Table S3** | Identification of 33 genes linked to large clone CH and CES.

symbol = Gene Symbol; chr = chromosome; start = Starting position of the gene; end = Ending position of the gene; IndSigSNPs = rsID of the independent significant SNPs that are in LD with the mapped SNPs.

| **symbol** | **chr** | **start** | **end** | **IndSigSNPs** |
| --- | --- | --- | --- | --- |
| PARP1 | 1 | 226548392 | 226595780 | rs76887998 |
| PRKCE | 2 | 45878484 | 46415129 | rs12615707 |
| GALNT5 | 2 | 158114110 | 158170723 | rs144815420 |
| C3orf80 | 3 | 159943423 | 159945999 | rs12632224 |
| RP11-432B6.3 | 3 | 159945241 | 160167617 | rs12632224 |
| TERT | 5 | 1253262 | 1295184 | rs7705526 |
| NHSL1 | 6 | 138743180 | 139013708 | rs117066841 |
| FGFR1OP | 6 | 167412670 | 167466201 | rs74893169 |
| CCR6 | 6 | 167525295 | 167553184 | rs74893169 |
| ZMYND11 | 10 | 180405 | 300577 | rs117942522 |
| DIP2C | 10 | 320130 | 735683 | rs117942522 |
| CD3G | 11 | 118215059 | 118225876 | rs73020212 |
| UBE4A | 11 | 118230300 | 118269926 | rs73020212 |
| RP11-770J1.5 | 11 | 118267810 | 118272610 | rs73020212 |
| ATP5L | 11 | 118271869 | 118302211 | rs73020212 |
| RP11-770J1.4 | 11 | 118303836 | 118305921 | rs73020212 |
| KMT2A | 11 | 118307205 | 118397539 | rs73020212 |
| SENP1 | 12 | 48436681 | 48500091 | rs2468948 |
| PFKM | 12 | 48498922 | 48540187 | rs2468948 |
| ASB8 | 12 | 48541571 | 48574996 | rs2468948 |
| C12orf68 | 12 | 48577366 | 48579709 | rs2468948 |
| DKFZP779L1853 | 12 | 48592170 | 48595814 | rs2468948 |
| OR10AD1 | 12 | 48596081 | 48597170 | rs2468948 |
| H1FNT | 12 | 48722763 | 48724062 | rs2468948 |
| ZNF641 | 12 | 48733791 | 48745197 | rs2468948 |
| AC024257.1 | 12 | 48759919 | 48761738 | rs2468948 |
| TNFSF11 | 13 | 43136872 | 43182149 | rs633137 |
| CDH13 | 16 | 82660408 | 83830204 | rs724952 |
| OSGIN1 | 16 | 83981887 | 83999937 | rs148557733 |
| NECAB2 | 16 | 84002237 | 84036381 | rs148557733 |
| PITPNC1 | 17 | 65373575 | 65693372 | rs7207177 |
| TK1 | 17 | 76170160 | 76183314 | rs8066979 |
| AFMID | 17 | 76183398 | 76203782 | rs8066979 |

**Table S4** | Related research of 33 genes linked to large clone CH and CES.

| **Name** | **Related research content** | **DOI** |
| --- | --- | --- |
| PARP1 | PARP1 drives post-stroke neuronal death and neuroinflammation through excessive activation causing bioenergetic collapse, oxidative stress, PAR-HK1-mediated mitochondrial dysfunction, and caspase-independent PARthanatos characterized by AIF/MIF nuclear translocation. | 10.1126/science.aad6872, 10.1111/jnc.15464,10.1096/fj.202302559R, |
|  |  | 10.1016/j.ejphar.2024.176557, 10.1016/j.bcp.2024.116174, |
|  |  | 10.1016/j.brainres.2015.07.023, 10.1007/s10495-020-01600-w, |
|  |  | 10.1016/j.neuroscience.2010.10.029, 10.1016/j.expneurol.2009.02.012 |
| SENP1 | SENP1 mitigates stroke damage by deSUMOylating proteins (e.g., Sirt3), reducing oxidative stress, apoptosis, and thrombosis, thereby preserving neurovascular function. | 10.1016/j.freeradbiomed.2021.05.026, 10.1111/cns.13398, |
|  |  | 10.1038/cddis.2016.290 |
| TERT | TERT exerts neuroprotective effects in stroke by inhibiting apoptosis, reducing oxidative stress and inflammation, and promoting neuronal survival and repair mechanisms. | 10.1007/s12013-024-01504-5, 10.1016/j.ijdevneu.2011.07.010, |
|  |  | 10.1016/j.neulet.2012.03.014, 10.7499/j.issn.1008-8830.2016.12.020 |
| CDH13 | CDH13 genetic polymorphisms influence ischemic stroke risk by regulating adiponectin levels and exhibiting synergistic interactions with diabetes | 10.3967/bes2017.004, 10.2337/db10-1321 |
| PRKCE | PRKCE plays a neuroprotective role in stroke by activating the NFE2L2/HMOX1 antioxidant pathway to mitigate cerebral ischemia/reperfusion injury and oxidative stress. | 10.1007/s12035-022-02848-w, 10.1590/1414-431X2024e13388, |
| CCR6 | CCR6 mediates IL-17+ γδ T cell migration into the brain, exacerbating stroke damage; its deficiency limits infiltration and improves outcomes. | 10.3389/fimmu.2021.635076, 10.1161/STROKEAHA.117.016753, |
|  |  | 10.1007/s11064-018-2632-6 |
| PFKM | PFKM exacerbates stroke injury via mTOR/HIF-1α-driven glycolysis and lactate. | 10.1111/cns.70314, 10.1007/s12031-012-9850-1 |
| KMT2A | KMT2A partners with GATA3 to boost H3K4-3me, upregulating NCX3 and enhancing ischemic preconditioning neuroprotection in stroke. | 10.1161/STROKEAHA.121.034637 |
| ATP5L | ATP5L (ATP5MG) in stroke links mitochondrial dysfunction to poor prognosis via ATP synthase. | 10.1515/med-2024-1050 |

**Table S5** | Differentially expressed genes between the control and IS group.

| **SYMBOL** | **log2FoldChange** | ***P*-value** | **change** |
| --- | --- | --- | --- |
| SLC25A42 | -0.510850235 | 7.27E-21 | Down |
| ID3 | -0.676599295 | 8.73E-21 | Down |
| NKTR | -0.521694096 | 1.34E-19 | Down |
| CD79B | -0.847931682 | 2.47E-18 | Down |
| APRT | -0.540736938 | 4.42E-18 | Down |
| FAM102A | -0.647676367 | 4.63E-17 | Down |
| ZAP70 | -0.566507283 | 4.69E-17 | Down |
| TNFRSF25 | -0.669651466 | 6.70E-17 | Down |
| LIME1 | -0.585090823 | 1.52E-15 | Down |
| GRAP | -0.592099602 | 1.78E-14 | Down |
| LTB | -0.516803387 | 1.50E-12 | Down |
| AES | -0.53210568 | 1.96E-12 | Down |
| EVL | -0.508099531 | 6.00E-12 | Down |
| RNF165 | -0.564508072 | 9.10E-11 | Down |
| CD19 | -0.629742238 | 1.51E-10 | Down |
| CCR7 | -0.762920709 | 1.52E-10 | Down |
| PASK | -0.525671245 | 4.22E-10 | Down |
| CD247 | -0.512963549 | 2.22E-09 | Down |
| CD79A | -0.698760026 | 8.29E-09 | Down |
| NELL2 | -0.573739323 | 9.44E-09 | Down |
| CLIC3 | -0.5434063 | 1.81E-08 | Down |
| VPREB3 | -0.555145259 | 9.26E-07 | Down |
| HLA-DQA1 | -0.605579934 | 0.009013441 | Down |
| PLXDC2 | 0.655830246 | 3.21E-26 | Up |
| LHFPL2 | 0.58990002 | 5.60E-21 | Up |
| ARG1 | 1.357154108 | 4.32E-19 | Up |
| SLC22A4 | 0.639025808 | 6.01E-18 | Up |
| CPD | 0.537776119 | 6.11E-16 | Up |
| VNN3 | 0.627293495 | 6.12E-16 | Up |
| CD163 | 0.661927439 | 1.10E-15 | Up |
| SAP30 | 0.743847537 | 1.23E-15 | Up |
| KIF1B | 0.535894935 | 1.50E-15 | Up |
| F5 | 0.629553175 | 2.09E-15 | Up |
| DUSP1 | 0.600411467 | 3.93E-14 | Up |
| ABHD5 | 0.550566932 | 6.17E-14 | Up |
| NFIL3 | 0.658645236 | 1.19E-13 | Up |
| ABCA1 | 0.643982772 | 3.99E-11 | Up |
| MMP9 | 0.954474254 | 9.48E-11 | Up |
| HIST1H4H | 0.603989384 | 1.35E-10 | Up |
| LRG1 | 0.541268064 | 2.48E-10 | Up |
| MCEMP1 | 0.722994814 | 3.39E-10 | Up |
| KCNJ15 | 0.547920843 | 4.28E-10 | Up |
| FCAR | 0.650916845 | 9.24E-10 | Up |
| FOS | 0.539826909 | 1.96E-09 | Up |
| IL1R2 | 0.632045022 | 3.79E-09 | Up |
| PRRG4 | 0.513147761 | 5.03E-09 | Up |
| CYP1B1 | 0.541180966 | 6.20E-09 | Up |
| TPST1 | 0.641814018 | 7.41E-09 | Up |
| TCN1 | 0.665006452 | 8.51E-09 | Up |
| ADM | 0.516265967 | 1.50E-08 | Up |
| ECHDC3 | 0.604920233 | 2.65E-08 | Up |
| DSC2 | 0.565051965 | 6.84E-08 | Up |
| S100A12 | 0.628592641 | 5.07E-07 | Up |
| PGLYRP1 | 0.540558576 | 2.24E-06 | Up |
| ANKRD9 | 0.556341127 | 2.79E-06 | Up |
| FOLR3 | 0.602420478 | 2.93E-05 | Up |
| LY96 | 0.555154877 | 6.81E-05 | Up |
| PTGS2 | 0.542141376 | 0.00010871 | Up |
| DEFA4 | 0.797246638 | 0.000109674 | Up |
| FECH | 0.54577027 | 0.000242235 | Up |
| OLFM4 | 0.659635983 | 0.000572625 | Up |
| CA1 | 0.657461898 | 0.001151392 | Up |

**Table S6** | Identification of module genes using WGCNA.

| SYMBOL | SYMBOL | SYMBOL |
| --- | --- | --- |
| RPL18 | FKBP11 | ASGR2 |
| SRGN | MCM3 | BNIP3 |
| RGS2 | SHMT2 | SAP30 |
| HLA-DRA | ITGAL | TCN1 |
| BTG1 | CYLD | GPR18 |
| CKLF | PARP1 | UCKL1 |
| HCST | NDUFV1 | UBE2G2 |
| S100A10 | PDE4B | ZNF395 |
| LTB | SRPRB | PFKP |
| EEF2 | VAMP1 | NCOA5 |
| PSMB10 | POGK | DPH3 |
| RPS4X | LAT | LTA |
| CDC37 | ARL2 | PPIH |
| CD247 | PPFIA1 | ZNF143 |
| ERP29 | ASGR1 | QDPR |
| FXYD5 | PRMT1 | BEX2 |
| ARHGAP9 | EMILIN2 | KIAA1598 |
| HLA-DPA1 | TAF1C | MAP3K4 |
| CD81 | FCGR1B | SLPI |
| HLA-DMB | EPRS | GTPBP8 |
| TSC22D3 | CYBRD1 | PLCG1 |
| CIB1 | LPIN1 | POLR1E |
| LFNG | AXIN1 | PLP2 |
| MDH2 | SDAD1 | KIAA0556 |
| EIF4B | DDX18 | SMARCD3 |
| ALKBH5 | PUS1 | UBQLN4 |
| CD3D | RBM4 | BST1 |
| NUP214 | C16orf58 | STK39 |
| ARHGEF18 | ARHGAP17 | LY9 |
| CRIP1 | TTC14 | SLC25A25 |
| CASP1 | RNPS1 | IL18R1 |
| LAMP2 | ACSL5 | CKAP5 |
| FNBP1 | RRAGD | PPP3CC |
| IL7R | KRT10 | DPH2 |
| PJA2 | TJAP1 | LXN |
| EDF1 | DCTN5 | ARID5B |
| GSTP1 | ITPR3 | MRPS7 |
| TXN | SPAG9 | UBASH3A |
| CALM1 | STX6 | GEMIN4 |
| MAL | ZNF512 | MRPS9 |
| EVL | DNAJA3 | RAB11FIP3 |
| MRPS6 | NUP88 | ACSL3 |
| SPOCK2 | KYNU | P2RY10 |
| MARCKS | ZBTB4 | RHOT2 |
| RFWD2 | MAP4K1 | ACSS1 |
| LIME1 | DOCK10 | FCGR1A |
| CYFIP2 | TRAPPC2 | ZNF200 |
| CTSH | SLC25A42 | CD163 |
| HLA-DMA | AOAH | ITM2C |
| NOSIP | PIK3R1 | LRIG1 |
| NONO | BCL2 | RP9 |
| CD2 | KIAA1147 | PPP1R16B |
| LPXN | MRPL24 | EXOSC7 |
| MFNG | SLC22A4 | PPIE |
| DARS | RBMS1 | TBC1D2 |
| ICAM2 | NPEPPS | TGIF1 |
| XBP1 | ABHD5 | L3MBTL2 |
| SERPINB1 | CEACAM1 | CIRH1A |
| CIRBP | TMC6 | SUV420H1 |
| NAP1L4 | PCSK7 | NQO2 |
| IMP3 | PRKCQ | CDK4 |
| BRD2 | BCL11B | ADCK2 |
| HIGD2A | MLLT6 | SLC7A6 |
| STX11 | TOMM40 | MGST1 |
| PIM2 | TOMM70A | ICOS |
| CCR7 | SULF2 | ZBTB24 |
| CHIC2 | ATIC | PRPF19 |
| RALGDS | SMARCD1 | DCTN4 |
| ARHGEF3 | SAMD3 | ATP8B2 |
| FAIM3 | RRM1 | CASP8 |
| CAMP | HAX1 | RNMTL1 |
| CTSS | UBE3C | OSGEP |
| CCND2 | SCAMP3 | SWAP70 |
| RPA2 | MACF1 | ID3 |
| CD79B | ZNF689 | IQGAP2 |
| AES | ITPKB | MYO5A |
| SLAMF6 | SUPT16H | PTPN7 |
| CUTA | TTC4 | ZBTB20 |
| HDAC1 | GPS1 | FLT3LG |
| SAE1 | RBP7 | MCCC1 |
| PRDX1 | C19orf12 | GORASP2 |
| CD55 | SBF1 | PAICS |
| DYRK2 | GOLGA3 | ST6GALNAC6 |
| CD7 | TNFRSF25 | PKIA |
| CCT8 | PLEKHG3 | TFB1M |
| EIF4E3 | PHPT1 | HLA-DOB |
| BIN1 | IMPDH2 | TSPAN18 |
| RAB32 | AARS | ANG |
| CENPB | EXOSC10 | CAPS |
| FAM50A | KIAA0355 | HLA-DOA |
| HSP90B1 | METAP1 | ACYP1 |
| LILRB2 | DEXI | PPM1D |
| RBL2 | TYSND1 | GADD45A |
| SNRPB | SUSD3 | C21orf2 |
| PPM1G | WDR54 | GPX7 |
| MLKL | TLR5 | SLC36A4 |
| SEC61A1 | ALDH2 | ARG1 |
| FBL | ADARB1 | SSBP4 |
| FNBP4 | FAM102A | UPF3A |
| GYG1 | WDR18 | OGFOD1 |
| PPP4R1 | CRELD2 | SLC24A4 |
| NGRN | CLEC7A | PRKCA |
| VAMP3 | RANGAP1 | DIDO1 |
| SLC38A1 | TTC19 | DHX30 |
| GRAP | HSPH1 | PASK |
| SVIL | PNPO | DFFA |
| NSMAF | PSMC3 | FKBP2 |
| APEX1 | IDH1 | NOV |
| PELI1 | CBLB | POMGNT1 |
| CD6 | TRPV2 | SNAP29 |
| CCT7 | C7orf26 | PCTP |
| DENND2D | ATP8B4 | AP3M2 |
| NHP2L1 | SLC35E3 | LHFPL2 |
| MCEMP1 | RNF126 | TBC1D4 |
| TUBB | D2HGDH | ASAH1 |
| TRAF3IP3 | SBK1 | UNG |
| CNOT1 | SIRPG | MDC1 |
| P2RY8 | FBXO21 | KLHL3 |
| CDC25B | PAQR8 | SUV39H1 |
| PRKCH | PRPS1 | NCR3 |
| SCPEP1 | KIF1B | FOXK1 |
| SLC30A7 | PRKDC | MRPS26 |
| ITK | ZAP70 | WDR4 |
| CHMP1B | APOL3 | NUP210 |
| NCL | CLSTN1 | OSBPL1A |
| GIMAP6 | DNAJC9 | SLAMF1 |
| CEP63 | CD63 | LDLRAP1 |
| NKTR | TRAF5 | FBXO31 |
| DNMT1 | RAB3IP | SIGIRR |
| CD79A | FN3KRP | CD5 |
| NUP62 | SDCCAG3 | CD19 |
| PTGS2 | TRRAP | TMEM63A |
| RASGRP2 | STUB1 | GNL1 |
| ZDHHC8 | MCM6 | FBXO32 |
| CD47 | BANK1 | ZNF438 |
| ABLIM1 | DDX24 | GSPT2 |
| FYN | DDX56 | TOP1MT |
| MAPRE2 | HSDL2 | WDR41 |
| SF3A3 | SMAD3 | TSPAN3 |
| SRPK1 | ZFP90 | LTB4R |
| CREBBP | CLEC2D | NAT10 |
| IDS | COPS7B | PRKX |
| TMED9 | SKAP1 | SNRPD3 |
| LEF1 | NFIL3 | TMEM116 |
| QRICH1 | IL18BP | CDC14A |
| APRT | CARD11 | KIAA0232 |
| VEGFB | PHF2 | CSTA |
| TRIM4 | RPUSD2 | LTBP3 |
| PEA15 | CXXC5 | PTPLAD1 |
| STAT4 | STIP1 | FCRL2 |
| RBM14 | ADAM17 | LUC7L |
| IMP4 | COQ2 | HIRIP3 |
| ATM | TRADD | ZCCHC11 |
| EML4 | PWP1 | ZNF329 |
| SNRPA1 | CYP1B1 | BTLA |
| CLTA | RNF135 | TCEA2 |
| M6PR | DNASE1L1 | RNASE4 |
| ABCA1 | LZTR1 | NPAT |
| MAP1LC3B | DGKA | SCRN1 |
| AKR1A1 | PHACTR4 | C9orf72 |
| ECHDC2 | CD99 | VPREB3 |
| ITGAM | NELL2 | BCL7B |
| IL11RA | MFGE8 | ARHGAP11A |
| RPUSD4 | GGA2 | TNFAIP8L1 |
| CYBB | H2AFJ | TMEM99 |
| TMEM173 | ILF3 | IARS |
| C9orf142 | ZBTB40 | LIG1 |
| ST6GAL1 | TRIB2 | RNF141 |
| THAP11 | STXBP5 | TRAPPC6A |
| PDE7A | CD3E | SLC25A22 |
| NT5C | AHCTF1 | ACACB |
| ASPSCR1 | RNMT | ZNF500 |
| UROS | ZNF337 | TCEA3 |
| IL10RA | BRSK1 | GIPC1 |
| LBH | TMEM109 | DUS3L |
| CD96 | BCAS4 | BBS2 |
| VDAC1 | SF3B3 | MCTP1 |
| HSD17B12 | LAX1 | ZNF548 |
| MCM7 | STAMBPL1 | KIFAP3 |
| ATP1A1 | CD3G | MTX3 |
| HSP90AB1 | CCDC25 | NANS |
| AKR1B1 | ZNF275 | NUDT9 |
| IVNS1ABP | NUP50 | HSD17B8 |
| MGST2 | PLXDC2 | RORA |
| WDR6 | WBSCR22 | CTDP1 |
| PLEKHA1 | ASNS | CLYBL |
| RGS19 | DSC2 | MUM1 |
| ITGB7 | ERO1L | PHF19 |
| ARL2BP | RNASEH1 | MRPL38 |
| CHST13 | GOT2 | CDK6 |
| FEZ2 | ERCC5 | GTF2H4 |
| TXLNA | MAGED1 | MLLT11 |
| CPD | NUP205 | ASGR2 |
| RCC2 | EIF4G3 | BNIP3 |
| RPL18 | FKBP11 | SAP30 |
| SRGN | MCM3 | TCN1 |
| RGS2 | SHMT2 | GPR18 |
| HLA-DRA | ITGAL | UCKL1 |
| BTG1 | CYLD | UBE2G2 |
| CKLF | PARP1 | ZNF395 |
| HCST | NDUFV1 | PFKP |
| S100A10 | PDE4B | NCOA5 |
| LTB | SRPRB | DPH3 |
| EEF2 | VAMP1 | LTA |
| PSMB10 | POGK | PPIH |
| RPS4X | LAT | ZNF143 |
| CDC37 | ARL2 | QDPR |
| CD247 | PPFIA1 | BEX2 |
| ERP29 | ASGR1 | KIAA1598 |
| FXYD5 | PRMT1 | MAP3K4 |
| ARHGAP9 | EMILIN2 | SLPI |
| HLA-DPA1 | TAF1C | GTPBP8 |
| CD81 | FCGR1B | PLCG1 |
| HLA-DMB | EPRS | POLR1E |
| TSC22D3 | CYBRD1 | PLP2 |
| CIB1 | LPIN1 | KIAA0556 |
| LFNG | AXIN1 | SMARCD3 |
| MDH2 | SDAD1 | UBQLN4 |
| EIF4B | DDX18 | BST1 |
| ALKBH5 | PUS1 | STK39 |
| CD3D | RBM4 | LY9 |
| NUP214 | C16orf58 | SLC25A25 |
| ARHGEF18 | ARHGAP17 | IL18R1 |
| CRIP1 | TTC14 | CKAP5 |
| CASP1 | RNPS1 | PPP3CC |
| LAMP2 | ACSL5 | DPH2 |
| FNBP1 | RRAGD | LXN |
| IL7R | KRT10 | ARID5B |
| PJA2 | TJAP1 | MRPS7 |
| EDF1 | DCTN5 | UBASH3A |
| GSTP1 | ITPR3 | GEMIN4 |
| TXN | SPAG9 | MRPS9 |
| CALM1 | STX6 | RAB11FIP3 |
| MAL | ZNF512 | ACSL3 |
| EVL | DNAJA3 | P2RY10 |
| MRPS6 | NUP88 | RHOT2 |
| SPOCK2 | KYNU | ACSS1 |
| MARCKS | ZBTB4 | FCGR1A |
| RFWD2 | MAP4K1 | ZNF200 |
| LIME1 | DOCK10 | CD163 |
| CYFIP2 | TRAPPC2 | ITM2C |
| CTSH | SLC25A42 | LRIG1 |
| HLA-DMA | AOAH | RP9 |
| NOSIP | PIK3R1 | PPP1R16B |
| NONO | BCL2 | EXOSC7 |
| CD2 | KIAA1147 | PPIE |
| LPXN | MRPL24 | TBC1D2 |
| MFNG | SLC22A4 | TGIF1 |
| DARS | RBMS1 | L3MBTL2 |
| ICAM2 | NPEPPS | CIRH1A |
| XBP1 | ABHD5 | SUV420H1 |
| SERPINB1 | CEACAM1 | NQO2 |
| CIRBP | TMC6 | CDK4 |
| NAP1L4 | PCSK7 | ADCK2 |
| IMP3 | PRKCQ | SLC7A6 |
| BRD2 | BCL11B | MGST1 |
| HIGD2A | MLLT6 | ICOS |
| STX11 | TOMM40 | ZBTB24 |
| PIM2 | TOMM70A | PRPF19 |
| CCR7 | SULF2 | DCTN4 |
| CHIC2 | ATIC | ATP8B2 |
| RALGDS | SMARCD1 | CASP8 |
| ARHGEF3 | SAMD3 | RNMTL1 |
| FAIM3 | RRM1 | OSGEP |
| CAMP | HAX1 | SWAP70 |
| CTSS | UBE3C | ID3 |
| CCND2 | SCAMP3 | IQGAP2 |
| RPA2 | MACF1 | MYO5A |
| CD79B | ZNF689 | PTPN7 |
| AES | ITPKB | ZBTB20 |
| SLAMF6 | SUPT16H | FLT3LG |
| CUTA | TTC4 | MCCC1 |
| HDAC1 | GPS1 | GORASP2 |
| SAE1 | RBP7 | PAICS |
| PRDX1 | C19orf12 | ST6GALNAC6 |
| CD55 | SBF1 | PKIA |
| DYRK2 | GOLGA3 | TFB1M |
| CD7 | TNFRSF25 | HLA-DOB |
| CCT8 | PLEKHG3 | TSPAN18 |
| EIF4E3 | PHPT1 | ANG |
| BIN1 | IMPDH2 | CAPS |
| RAB32 | AARS | HLA-DOA |
| CENPB | EXOSC10 | ACYP1 |
| FAM50A | KIAA0355 | PPM1D |
| HSP90B1 | METAP1 | GADD45A |
| LILRB2 | DEXI | C21orf2 |
| RBL2 | TYSND1 | GPX7 |
| SNRPB | SUSD3 | SLC36A4 |
| PPM1G | WDR54 | ARG1 |
| MLKL | TLR5 | SSBP4 |
| SEC61A1 | ALDH2 | UPF3A |
| FBL | ADARB1 | OGFOD1 |
| FNBP4 | FAM102A | SLC24A4 |
| GYG1 | WDR18 | PRKCA |
| PPP4R1 | CRELD2 | DIDO1 |
| NGRN | CLEC7A | DHX30 |
| VAMP3 | RANGAP1 | PASK |
| SLC38A1 | TTC19 | DFFA |
| GRAP | HSPH1 | FKBP2 |
| SVIL | PNPO | NOV |
| NSMAF | PSMC3 | POMGNT1 |
| APEX1 | IDH1 | SNAP29 |
| PELI1 | CBLB | PCTP |
| CD6 | TRPV2 | AP3M2 |
| CCT7 | C7orf26 | LHFPL2 |
| DENND2D | ATP8B4 | TBC1D4 |
| NHP2L1 | SLC35E3 | ASAH1 |
| MCEMP1 | RNF126 | UNG |
| TUBB | D2HGDH | MDC1 |
| TRAF3IP3 | SBK1 | KLHL3 |
| CNOT1 | SIRPG | SUV39H1 |
| P2RY8 | FBXO21 | NCR3 |
| CDC25B | PAQR8 | FOXK1 |
| PRKCH | PRPS1 | MRPS26 |
| SCPEP1 | KIF1B | WDR4 |
| SLC30A7 | PRKDC | NUP210 |
| ITK | ZAP70 | OSBPL1A |
| CHMP1B | APOL3 | SLAMF1 |
| NCL | CLSTN1 | LDLRAP1 |
| GIMAP6 | DNAJC9 | FBXO31 |
| CEP63 | CD63 | SIGIRR |
| NKTR | TRAF5 | CD5 |
| DNMT1 | RAB3IP | CD19 |
| CD79A | FN3KRP | TMEM63A |
| NUP62 | SDCCAG3 | GNL1 |
| PTGS2 | TRRAP | FBXO32 |
| RASGRP2 | STUB1 | ZNF438 |
| ZDHHC8 | MCM6 | GSPT2 |
| CD47 | BANK1 | TOP1MT |
| ABLIM1 | DDX24 | WDR41 |
| FYN | DDX56 | TSPAN3 |
| MAPRE2 | HSDL2 | LTB4R |
| SF3A3 | SMAD3 | NAT10 |
| SRPK1 | ZFP90 | PRKX |
| CREBBP | CLEC2D | SNRPD3 |
| IDS | COPS7B | TMEM116 |
| TMED9 | SKAP1 | CDC14A |
| LEF1 | NFIL3 | KIAA0232 |
| QRICH1 | IL18BP | CSTA |
| APRT | CARD11 | LTBP3 |
| VEGFB | PHF2 | PTPLAD1 |
| TRIM4 | RPUSD2 | FCRL2 |
| PEA15 | CXXC5 | LUC7L |
| STAT4 | STIP1 | HIRIP3 |
| RBM14 | ADAM17 | ZCCHC11 |
| IMP4 | COQ2 | ZNF329 |
| ATM | TRADD | BTLA |
| EML4 | PWP1 | TCEA2 |
| SNRPA1 | CYP1B1 | RNASE4 |
| CLTA | RNF135 | NPAT |
| M6PR | DNASE1L1 | SCRN1 |
| ABCA1 | LZTR1 | C9orf72 |
| MAP1LC3B | DGKA | VPREB3 |
| AKR1A1 | PHACTR4 | BCL7B |
| ECHDC2 | CD99 | ARHGAP11A |
| ITGAM | NELL2 | TNFAIP8L1 |
| IL11RA | MFGE8 | TMEM99 |
| RPUSD4 | GGA2 | IARS |
| CYBB | H2AFJ | LIG1 |
| TMEM173 | ILF3 | RNF141 |
| C9orf142 | ZBTB40 | TRAPPC6A |
| ST6GAL1 | TRIB2 | SLC25A22 |
| THAP11 | STXBP5 | ACACB |
| PDE7A | CD3E | ZNF500 |
| NT5C | AHCTF1 | TCEA3 |
| ASPSCR1 | RNMT | GIPC1 |
| UROS | ZNF337 | DUS3L |
| IL10RA | BRSK1 | BBS2 |
| LBH | TMEM109 | MCTP1 |
| CD96 | BCAS4 | ZNF548 |
| VDAC1 | SF3B3 | KIFAP3 |
| HSD17B12 | LAX1 | MTX3 |
| MCM7 | STAMBPL1 | NANS |
| ATP1A1 | CD3G | NUDT9 |
| HSP90AB1 | CCDC25 | HSD17B8 |
| AKR1B1 | ZNF275 | RORA |
| IVNS1ABP | NUP50 | CTDP1 |
| MGST2 | PLXDC2 | CLYBL |
| WDR6 | WBSCR22 | MUM1 |
| PLEKHA1 | ASNS | PHF19 |
| RGS19 | DSC2 | MRPL38 |
| ITGB7 | ERO1L | CDK6 |
| ARL2BP | RNASEH1 | GTF2H4 |
| CHST13 | GOT2 | MLLT11 |
| FEZ2 | ERCC5 | ASGR2 |
| TXLNA | MAGED1 |  |
| CPD | NUP205 |  |

**Table S7** | The full performance metrics (AUCs) for all 113 models.

| **Method** | **Train** | **GSE162955** |
| --- | --- | --- |
| Lasso+Stepglm[both] | 0.931 | 0.694 |
| SVM | 0.898 | 0.417 |
| glmBoost+SVM | 0.882 | 0.500 |
| Ridge | 0.926 | 0.667 |
| Lasso+SVM | 0.902 | 0.417 |
| glmBoost+Ridge | 0.915 | 0.583 |
| Enet[alpha=0.1] | 0.929 | 0.667 |
| glmBoost+Enet[alpha=0.1] | 0.918 | 0.639 |
| Enet[alpha=0.2] | 0.930 | 0.667 |
| Enet[alpha=0.3] | 0.928 | 0.667 |
| glmBoost+Enet[alpha=0.3] | 0.917 | 0.639 |
| glmBoost+Enet[alpha=0.2] | 0.917 | 0.639 |
| Enet[alpha=0.4] | 0.929 | 0.667 |
| glmBoost+Enet[alpha=0.4] | 0.917 | 0.611 |
| Lasso+glmBoost | 0.903 | 0.694 |
| Enet[alpha=0.5] | 0.929 | 0.667 |
| glmBoost | 0.902 | 0.694 |
| glmBoost+Enet[alpha=0.5] | 0.916 | 0.639 |
| Enet[alpha=0.6] | 0.929 | 0.667 |
| glmBoost+Enet[alpha=0.6] | 0.917 | 0.639 |
| glmBoost+Enet[alpha=0.7] | 0.916 | 0.611 |
| glmBoost+Enet[alpha=0.8] | 0.917 | 0.639 |
| Enet[alpha=0.8] | 0.928 | 0.667 |
| Enet[alpha=0.9] | 0.929 | 0.667 |
| Lasso | 0.929 | 0.667 |
| Enet[alpha=0.7] | 0.927 | 0.667 |
| glmBoost+Enet[alpha=0.9] | 0.917 | 0.639 |
| glmBoost+Lasso | 0.918 | 0.639 |
| Lasso+plsRglm | 0.908 | 0.750 |
| glmBoost+plsRglm | 0.903 | 0.611 |
| glmBoost+Stepglm[forward] | 0.919 | 0.639 |
| Lasso+Stepglm[forward] | 0.932 | 0.694 |
| Stepglm[forward] | 0.932 | 0.694 |
| plsRglm | 0.899 | 0.806 |
| Stepglm[both]+Ridge | 0.925 | 0.667 |
| Stepglm[backward]+Ridge | 0.925 | 0.667 |
| Stepglm[both]+plsRglm | 0.909 | 0.750 |
| Stepglm[backward]+plsRglm | 0.909 | 0.750 |
| Stepglm[both]+Enet[alpha=0.9] | 0.930 | 0.667 |
| Stepglm[backward]+Enet[alpha=0.9] | 0.930 | 0.667 |
| Stepglm[both]+Enet[alpha=0.1] | 0.930 | 0.667 |
| Stepglm[backward]+Enet[alpha=0.1] | 0.929 | 0.667 |
| Stepglm[both]+Enet[alpha=0.8] | 0.929 | 0.667 |
| Stepglm[backward]+Enet[alpha=0.8] | 0.928 | 0.667 |
| Stepglm[both]+Enet[alpha=0.2] | 0.928 | 0.667 |
| Stepglm[backward]+Enet[alpha=0.2] | 0.929 | 0.667 |
| Stepglm[both]+Lasso | 0.929 | 0.667 |
| Stepglm[backward]+Lasso | 0.929 | 0.667 |
| Stepglm[both]+Enet[alpha=0.6] | 0.929 | 0.667 |
| Stepglm[backward]+Enet[alpha=0.6] | 0.930 | 0.667 |
| glmBoost+GBM | 0.990 | 0.694 |
| Stepglm[both]+Enet[alpha=0.7] | 0.929 | 0.667 |
| Stepglm[backward]+Enet[alpha=0.7] | 0.929 | 0.667 |
| Lasso+Stepglm[backward] | 0.931 | 0.694 |
| Stepglm[both] | 0.931 | 0.694 |
| Stepglm[backward] | 0.931 | 0.694 |
| glmBoost+Stepglm[both] | 0.914 | 0.667 |
| glmBoost+Stepglm[backward] | 0.914 | 0.667 |
| Stepglm[both]+Enet[alpha=0.4] | 0.930 | 0.667 |
| Stepglm[backward]+Enet[alpha=0.4] | 0.929 | 0.667 |
| Stepglm[both]+Enet[alpha=0.3] | 0.929 | 0.667 |
| Stepglm[backward]+Enet[alpha=0.3] | 0.928 | 0.667 |
| Stepglm[both]+glmBoost | 0.902 | 0.694 |
| Stepglm[backward]+glmBoost | 0.902 | 0.694 |
| Stepglm[both]+Enet[alpha=0.5] | 0.929 | 0.667 |
| Stepglm[backward]+Enet[alpha=0.5] | 0.930 | 0.667 |
| Lasso+GBM | 0.994 | 0.667 |
| GBM | 0.992 | 0.694 |
| Stepglm[both]+SVM | 0.883 | 0.500 |
| Stepglm[backward]+SVM | 0.876 | 0.500 |
| Stepglm[both]+GBM | 0.996 | 0.583 |
| Stepglm[backward]+GBM | 0.986 | 0.583 |
| LDA | 0.930 | 0.667 |
| glmBoost+LDA | 0.917 | 0.639 |
| Stepglm[both]+LDA | 0.928 | 0.611 |
| Stepglm[backward]+LDA | 0.928 | 0.611 |
| Lasso+LDA | 0.929 | 0.639 |
| XGBoost | 0.833 | 0.639 |
| Lasso+XGBoost | 0.931 | 0.667 |
| glmBoost+XGBoost | 0.980 | 0.667 |
| Stepglm[both]+XGBoost | 0.958 | 0.583 |
| Stepglm[backward]+XGBoost | 0.986 | 0.597 |
| NaiveBayes | 0.829 | 0.750 |
| Lasso+NaiveBayes | 0.837 | 0.750 |
| glmBoost+NaiveBayes | 0.859 | 0.667 |
| Stepglm[both]+NaiveBayes | 0.837 | 0.722 |
| Stepglm[backward]+NaiveBayes | 0.837 | 0.722 |

**Table S8** | The detailed features selected by 113 combinations of ML models.

| **Features** | **Algorithm** |
| --- | --- |
| B2M | Lasso+Stepglm[both] |
| CD3D | Lasso+Stepglm[both] |
| SYK | Lasso+Stepglm[both] |
| POLB | Lasso+Stepglm[both] |
| PARP1 | Lasso+Stepglm[both] |
| CASP3 | Lasso+Stepglm[both] |
| ZAP70 | Lasso+Stepglm[both] |
| SMARCA4 | Lasso+Stepglm[both] |
| CD3E | Lasso+Stepglm[both] |
| CD3G | Lasso+Stepglm[both] |
| B2M | SVM |
| CD247 | SVM |
| CD3D | SVM |
| SYK | SVM |
| CD8A | SVM |
| POLB | SVM |
| XRCC6 | SVM |
| PARP1 | SVM |
| CASP3 | SVM |
| PRKDC | SVM |
| ZAP70 | SVM |
| SMARCA4 | SVM |
| XRCC5 | SVM |
| CD3E | SVM |
| CD3G | SVM |
| CASP9 | SVM |
| B2M | glmBoost+SVM |
| SYK | glmBoost+SVM |
| POLB | glmBoost+SVM |
| PARP1 | glmBoost+SVM |
| CASP3 | glmBoost+SVM |
| ZAP70 | glmBoost+SVM |
| SMARCA4 | glmBoost+SVM |
| CD3G | glmBoost+SVM |
| CASP9 | glmBoost+SVM |
| B2M | Ridge |
| CD247 | Ridge |
| CD3D | Ridge |
| SYK | Ridge |
| CD8A | Ridge |
| POLB | Ridge |
| XRCC6 | Ridge |
| PARP1 | Ridge |
| CASP3 | Ridge |
| PRKDC | Ridge |
| ZAP70 | Ridge |
| SMARCA4 | Ridge |
| XRCC5 | Ridge |
| CD3E | Ridge |
| CD3G | Ridge |
| CASP9 | Ridge |
| B2M | Lasso+SVM |
| CD247 | Lasso+SVM |
| CD3D | Lasso+SVM |
| SYK | Lasso+SVM |
| POLB | Lasso+SVM |
| PARP1 | Lasso+SVM |
| CASP3 | Lasso+SVM |
| PRKDC | Lasso+SVM |
| ZAP70 | Lasso+SVM |
| SMARCA4 | Lasso+SVM |
| CD3E | Lasso+SVM |
| CD3G | Lasso+SVM |
| CASP9 | Lasso+SVM |
| B2M | glmBoost+Ridge |
| SYK | glmBoost+Ridge |
| POLB | glmBoost+Ridge |
| PARP1 | glmBoost+Ridge |
| CASP3 | glmBoost+Ridge |
| ZAP70 | glmBoost+Ridge |
| SMARCA4 | glmBoost+Ridge |
| CD3G | glmBoost+Ridge |
| CASP9 | glmBoost+Ridge |
| B2M | Enet[alpha=0.1] |
| CD247 | Enet[alpha=0.1] |
| CD3D | Enet[alpha=0.1] |
| SYK | Enet[alpha=0.1] |
| POLB | Enet[alpha=0.1] |
| XRCC6 | Enet[alpha=0.1] |
| PARP1 | Enet[alpha=0.1] |
| CASP3 | Enet[alpha=0.1] |
| PRKDC | Enet[alpha=0.1] |
| ZAP70 | Enet[alpha=0.1] |
| SMARCA4 | Enet[alpha=0.1] |
| XRCC5 | Enet[alpha=0.1] |
| CD3E | Enet[alpha=0.1] |
| CD3G | Enet[alpha=0.1] |
| CASP9 | Enet[alpha=0.1] |
| B2M | glmBoost+Enet[alpha=0.1] |
| SYK | glmBoost+Enet[alpha=0.1] |
| POLB | glmBoost+Enet[alpha=0.1] |
| PARP1 | glmBoost+Enet[alpha=0.1] |
| CASP3 | glmBoost+Enet[alpha=0.1] |
| ZAP70 | glmBoost+Enet[alpha=0.1] |
| SMARCA4 | glmBoost+Enet[alpha=0.1] |
| CD3G | glmBoost+Enet[alpha=0.1] |
| CASP9 | glmBoost+Enet[alpha=0.1] |
| B2M | Enet[alpha=0.2] |
| CD247 | Enet[alpha=0.2] |
| CD3D | Enet[alpha=0.2] |
| SYK | Enet[alpha=0.2] |
| POLB | Enet[alpha=0.2] |
| XRCC6 | Enet[alpha=0.2] |
| PARP1 | Enet[alpha=0.2] |
| CASP3 | Enet[alpha=0.2] |
| PRKDC | Enet[alpha=0.2] |
| ZAP70 | Enet[alpha=0.2] |
| SMARCA4 | Enet[alpha=0.2] |
| XRCC5 | Enet[alpha=0.2] |
| CD3E | Enet[alpha=0.2] |
| CD3G | Enet[alpha=0.2] |
| CASP9 | Enet[alpha=0.2] |
| B2M | Enet[alpha=0.3] |
| CD247 | Enet[alpha=0.3] |
| CD3D | Enet[alpha=0.3] |
| SYK | Enet[alpha=0.3] |
| POLB | Enet[alpha=0.3] |
| XRCC6 | Enet[alpha=0.3] |
| PARP1 | Enet[alpha=0.3] |
| CASP3 | Enet[alpha=0.3] |
| PRKDC | Enet[alpha=0.3] |
| ZAP70 | Enet[alpha=0.3] |
| SMARCA4 | Enet[alpha=0.3] |
| CD3E | Enet[alpha=0.3] |
| CD3G | Enet[alpha=0.3] |
| CASP9 | Enet[alpha=0.3] |
| B2M | glmBoost+Enet[alpha=0.3] |
| SYK | glmBoost+Enet[alpha=0.3] |
| POLB | glmBoost+Enet[alpha=0.3] |
| PARP1 | glmBoost+Enet[alpha=0.3] |
| CASP3 | glmBoost+Enet[alpha=0.3] |
| ZAP70 | glmBoost+Enet[alpha=0.3] |
| SMARCA4 | glmBoost+Enet[alpha=0.3] |
| CD3G | glmBoost+Enet[alpha=0.3] |
| CASP9 | glmBoost+Enet[alpha=0.3] |
| B2M | glmBoost+Enet[alpha=0.2] |
| SYK | glmBoost+Enet[alpha=0.2] |
| POLB | glmBoost+Enet[alpha=0.2] |
| PARP1 | glmBoost+Enet[alpha=0.2] |
| CASP3 | glmBoost+Enet[alpha=0.2] |
| ZAP70 | glmBoost+Enet[alpha=0.2] |
| SMARCA4 | glmBoost+Enet[alpha=0.2] |
| CD3G | glmBoost+Enet[alpha=0.2] |
| CASP9 | glmBoost+Enet[alpha=0.2] |
| B2M | Enet[alpha=0.4] |
| CD247 | Enet[alpha=0.4] |
| CD3D | Enet[alpha=0.4] |
| SYK | Enet[alpha=0.4] |
| POLB | Enet[alpha=0.4] |
| XRCC6 | Enet[alpha=0.4] |
| PARP1 | Enet[alpha=0.4] |
| CASP3 | Enet[alpha=0.4] |
| PRKDC | Enet[alpha=0.4] |
| ZAP70 | Enet[alpha=0.4] |
| SMARCA4 | Enet[alpha=0.4] |
| CD3E | Enet[alpha=0.4] |
| CD3G | Enet[alpha=0.4] |
| CASP9 | Enet[alpha=0.4] |
| B2M | glmBoost+Enet[alpha=0.4] |
| SYK | glmBoost+Enet[alpha=0.4] |
| POLB | glmBoost+Enet[alpha=0.4] |
| PARP1 | glmBoost+Enet[alpha=0.4] |
| CASP3 | glmBoost+Enet[alpha=0.4] |
| ZAP70 | glmBoost+Enet[alpha=0.4] |
| SMARCA4 | glmBoost+Enet[alpha=0.4] |
| CD3G | glmBoost+Enet[alpha=0.4] |
| CASP9 | glmBoost+Enet[alpha=0.4] |
| B2M | Lasso+glmBoost |
| SYK | Lasso+glmBoost |
| POLB | Lasso+glmBoost |
| PARP1 | Lasso+glmBoost |
| CASP3 | Lasso+glmBoost |
| ZAP70 | Lasso+glmBoost |
| SMARCA4 | Lasso+glmBoost |
| CD3G | Lasso+glmBoost |
| CASP9 | Lasso+glmBoost |
| B2M | Enet[alpha=0.5] |
| CD247 | Enet[alpha=0.5] |
| CD3D | Enet[alpha=0.5] |
| SYK | Enet[alpha=0.5] |
| POLB | Enet[alpha=0.5] |
| XRCC6 | Enet[alpha=0.5] |
| PARP1 | Enet[alpha=0.5] |
| CASP3 | Enet[alpha=0.5] |
| PRKDC | Enet[alpha=0.5] |
| ZAP70 | Enet[alpha=0.5] |
| SMARCA4 | Enet[alpha=0.5] |
| CD3E | Enet[alpha=0.5] |
| CD3G | Enet[alpha=0.5] |
| CASP9 | Enet[alpha=0.5] |
| B2M | glmBoost |
| SYK | glmBoost |
| POLB | glmBoost |
| PARP1 | glmBoost |
| CASP3 | glmBoost |
| ZAP70 | glmBoost |
| SMARCA4 | glmBoost |
| CD3G | glmBoost |
| CASP9 | glmBoost |
| B2M | glmBoost+Enet[alpha=0.5] |
| SYK | glmBoost+Enet[alpha=0.5] |
| POLB | glmBoost+Enet[alpha=0.5] |
| PARP1 | glmBoost+Enet[alpha=0.5] |
| CASP3 | glmBoost+Enet[alpha=0.5] |
| ZAP70 | glmBoost+Enet[alpha=0.5] |
| SMARCA4 | glmBoost+Enet[alpha=0.5] |
| CD3G | glmBoost+Enet[alpha=0.5] |
| CASP9 | glmBoost+Enet[alpha=0.5] |
| B2M | Enet[alpha=0.6] |
| CD247 | Enet[alpha=0.6] |
| CD3D | Enet[alpha=0.6] |
| SYK | Enet[alpha=0.6] |
| POLB | Enet[alpha=0.6] |
| XRCC6 | Enet[alpha=0.6] |
| PARP1 | Enet[alpha=0.6] |
| CASP3 | Enet[alpha=0.6] |
| PRKDC | Enet[alpha=0.6] |
| ZAP70 | Enet[alpha=0.6] |
| SMARCA4 | Enet[alpha=0.6] |
| CD3E | Enet[alpha=0.6] |
| CD3G | Enet[alpha=0.6] |
| CASP9 | Enet[alpha=0.6] |
| B2M | glmBoost+Enet[alpha=0.6] |
| SYK | glmBoost+Enet[alpha=0.6] |
| POLB | glmBoost+Enet[alpha=0.6] |
| PARP1 | glmBoost+Enet[alpha=0.6] |
| CASP3 | glmBoost+Enet[alpha=0.6] |
| ZAP70 | glmBoost+Enet[alpha=0.6] |
| SMARCA4 | glmBoost+Enet[alpha=0.6] |
| CD3G | glmBoost+Enet[alpha=0.6] |
| CASP9 | glmBoost+Enet[alpha=0.6] |
| B2M | glmBoost+Enet[alpha=0.7] |
| SYK | glmBoost+Enet[alpha=0.7] |
| POLB | glmBoost+Enet[alpha=0.7] |
| PARP1 | glmBoost+Enet[alpha=0.7] |
| CASP3 | glmBoost+Enet[alpha=0.7] |
| ZAP70 | glmBoost+Enet[alpha=0.7] |
| SMARCA4 | glmBoost+Enet[alpha=0.7] |
| CD3G | glmBoost+Enet[alpha=0.7] |
| CASP9 | glmBoost+Enet[alpha=0.7] |
| B2M | glmBoost+Enet[alpha=0.8] |
| SYK | glmBoost+Enet[alpha=0.8] |
| POLB | glmBoost+Enet[alpha=0.8] |
| PARP1 | glmBoost+Enet[alpha=0.8] |
| CASP3 | glmBoost+Enet[alpha=0.8] |
| ZAP70 | glmBoost+Enet[alpha=0.8] |
| SMARCA4 | glmBoost+Enet[alpha=0.8] |
| CD3G | glmBoost+Enet[alpha=0.8] |
| CASP9 | glmBoost+Enet[alpha=0.8] |
| B2M | Enet[alpha=0.8] |
| CD247 | Enet[alpha=0.8] |
| CD3D | Enet[alpha=0.8] |
| SYK | Enet[alpha=0.8] |
| POLB | Enet[alpha=0.8] |
| PARP1 | Enet[alpha=0.8] |
| CASP3 | Enet[alpha=0.8] |
| PRKDC | Enet[alpha=0.8] |
| ZAP70 | Enet[alpha=0.8] |
| SMARCA4 | Enet[alpha=0.8] |
| CD3E | Enet[alpha=0.8] |
| CD3G | Enet[alpha=0.8] |
| CASP9 | Enet[alpha=0.8] |
| B2M | Enet[alpha=0.9] |
| CD247 | Enet[alpha=0.9] |
| CD3D | Enet[alpha=0.9] |
| SYK | Enet[alpha=0.9] |
| POLB | Enet[alpha=0.9] |
| PARP1 | Enet[alpha=0.9] |
| CASP3 | Enet[alpha=0.9] |
| PRKDC | Enet[alpha=0.9] |
| ZAP70 | Enet[alpha=0.9] |
| SMARCA4 | Enet[alpha=0.9] |
| CD3E | Enet[alpha=0.9] |
| CD3G | Enet[alpha=0.9] |
| CASP9 | Enet[alpha=0.9] |
| B2M | Lasso |
| CD247 | Lasso |
| CD3D | Lasso |
| SYK | Lasso |
| POLB | Lasso |
| PARP1 | Lasso |
| CASP3 | Lasso |
| PRKDC | Lasso |
| ZAP70 | Lasso |
| SMARCA4 | Lasso |
| CD3E | Lasso |
| CD3G | Lasso |
| CASP9 | Lasso |
| B2M | Enet[alpha=0.7] |
| CD247 | Enet[alpha=0.7] |
| CD3D | Enet[alpha=0.7] |
| SYK | Enet[alpha=0.7] |
| POLB | Enet[alpha=0.7] |
| PARP1 | Enet[alpha=0.7] |
| CASP3 | Enet[alpha=0.7] |
| PRKDC | Enet[alpha=0.7] |
| ZAP70 | Enet[alpha=0.7] |
| SMARCA4 | Enet[alpha=0.7] |
| CD3E | Enet[alpha=0.7] |
| CD3G | Enet[alpha=0.7] |
| CASP9 | Enet[alpha=0.7] |
| B2M | glmBoost+Enet[alpha=0.9] |
| SYK | glmBoost+Enet[alpha=0.9] |
| POLB | glmBoost+Enet[alpha=0.9] |
| PARP1 | glmBoost+Enet[alpha=0.9] |
| CASP3 | glmBoost+Enet[alpha=0.9] |
| ZAP70 | glmBoost+Enet[alpha=0.9] |
| SMARCA4 | glmBoost+Enet[alpha=0.9] |
| CD3G | glmBoost+Enet[alpha=0.9] |
| CASP9 | glmBoost+Enet[alpha=0.9] |
| B2M | glmBoost+Lasso |
| SYK | glmBoost+Lasso |
| POLB | glmBoost+Lasso |
| PARP1 | glmBoost+Lasso |
| CASP3 | glmBoost+Lasso |
| ZAP70 | glmBoost+Lasso |
| SMARCA4 | glmBoost+Lasso |
| CD3G | glmBoost+Lasso |
| CASP9 | glmBoost+Lasso |
| B2M | Lasso+plsRglm |
| CD247 | Lasso+plsRglm |
| CD3D | Lasso+plsRglm |
| SYK | Lasso+plsRglm |
| POLB | Lasso+plsRglm |
| PARP1 | Lasso+plsRglm |
| CASP3 | Lasso+plsRglm |
| PRKDC | Lasso+plsRglm |
| ZAP70 | Lasso+plsRglm |
| SMARCA4 | Lasso+plsRglm |
| CD3E | Lasso+plsRglm |
| CD3G | Lasso+plsRglm |
| CASP9 | Lasso+plsRglm |
| POLB | glmBoost+plsRglm |
| PARP1 | glmBoost+plsRglm |
| CASP3 | glmBoost+plsRglm |
| ZAP70 | glmBoost+plsRglm |
| SMARCA4 | glmBoost+plsRglm |
| CD3G | glmBoost+plsRglm |
| CASP9 | glmBoost+plsRglm |
| B2M | glmBoost+Stepglm[forward] |
| SYK | glmBoost+Stepglm[forward] |
| POLB | glmBoost+Stepglm[forward] |
| PARP1 | glmBoost+Stepglm[forward] |
| CASP3 | glmBoost+Stepglm[forward] |
| ZAP70 | glmBoost+Stepglm[forward] |
| SMARCA4 | glmBoost+Stepglm[forward] |
| CD3G | glmBoost+Stepglm[forward] |
| CASP9 | glmBoost+Stepglm[forward] |
| B2M | Lasso+Stepglm[forward] |
| CD247 | Lasso+Stepglm[forward] |
| CD3D | Lasso+Stepglm[forward] |
| SYK | Lasso+Stepglm[forward] |
| POLB | Lasso+Stepglm[forward] |
| PARP1 | Lasso+Stepglm[forward] |
| CASP3 | Lasso+Stepglm[forward] |
| PRKDC | Lasso+Stepglm[forward] |
| ZAP70 | Lasso+Stepglm[forward] |
| SMARCA4 | Lasso+Stepglm[forward] |
| CD3E | Lasso+Stepglm[forward] |
| CD3G | Lasso+Stepglm[forward] |
| CASP9 | Lasso+Stepglm[forward] |
| B2M | Stepglm[forward] |
| CD247 | Stepglm[forward] |
| CD3D | Stepglm[forward] |
| SYK | Stepglm[forward] |
| CD8A | Stepglm[forward] |
| POLB | Stepglm[forward] |
| XRCC6 | Stepglm[forward] |
| PARP1 | Stepglm[forward] |
| CASP3 | Stepglm[forward] |
| PRKDC | Stepglm[forward] |
| ZAP70 | Stepglm[forward] |
| SMARCA4 | Stepglm[forward] |
| XRCC5 | Stepglm[forward] |
| CD3E | Stepglm[forward] |
| CD3G | Stepglm[forward] |
| CASP9 | Stepglm[forward] |
| B2M | plsRglm |
| CD247 | plsRglm |
| CD3D | plsRglm |
| CD8A | plsRglm |
| POLB | plsRglm |
| XRCC6 | plsRglm |
| PARP1 | plsRglm |
| CASP3 | plsRglm |
| PRKDC | plsRglm |
| ZAP70 | plsRglm |
| SMARCA4 | plsRglm |
| CD3E | plsRglm |
| CD3G | plsRglm |
| CASP9 | plsRglm |
| B2M | Stepglm[both]+Ridge |
| CD3D | Stepglm[both]+Ridge |
| SYK | Stepglm[both]+Ridge |
| POLB | Stepglm[both]+Ridge |
| PARP1 | Stepglm[both]+Ridge |
| CASP3 | Stepglm[both]+Ridge |
| ZAP70 | Stepglm[both]+Ridge |
| SMARCA4 | Stepglm[both]+Ridge |
| CD3E | Stepglm[both]+Ridge |
| CD3G | Stepglm[both]+Ridge |
| B2M | Stepglm[backward]+Ridge |
| CD3D | Stepglm[backward]+Ridge |
| SYK | Stepglm[backward]+Ridge |
| POLB | Stepglm[backward]+Ridge |
| PARP1 | Stepglm[backward]+Ridge |
| CASP3 | Stepglm[backward]+Ridge |
| ZAP70 | Stepglm[backward]+Ridge |
| SMARCA4 | Stepglm[backward]+Ridge |
| CD3E | Stepglm[backward]+Ridge |
| CD3G | Stepglm[backward]+Ridge |
| B2M | Stepglm[both]+plsRglm |
| CD3D | Stepglm[both]+plsRglm |
| POLB | Stepglm[both]+plsRglm |
| PARP1 | Stepglm[both]+plsRglm |
| CASP3 | Stepglm[both]+plsRglm |
| ZAP70 | Stepglm[both]+plsRglm |
| SMARCA4 | Stepglm[both]+plsRglm |
| CD3E | Stepglm[both]+plsRglm |
| CD3G | Stepglm[both]+plsRglm |
| B2M | Stepglm[backward]+plsRglm |
| CD3D | Stepglm[backward]+plsRglm |
| POLB | Stepglm[backward]+plsRglm |
| PARP1 | Stepglm[backward]+plsRglm |
| CASP3 | Stepglm[backward]+plsRglm |
| ZAP70 | Stepglm[backward]+plsRglm |
| SMARCA4 | Stepglm[backward]+plsRglm |
| CD3E | Stepglm[backward]+plsRglm |
| CD3G | Stepglm[backward]+plsRglm |
| B2M | Stepglm[both]+Enet[alpha=0.9] |
| CD3D | Stepglm[both]+Enet[alpha=0.9] |
| SYK | Stepglm[both]+Enet[alpha=0.9] |
| POLB | Stepglm[both]+Enet[alpha=0.9] |
| PARP1 | Stepglm[both]+Enet[alpha=0.9] |
| CASP3 | Stepglm[both]+Enet[alpha=0.9] |
| ZAP70 | Stepglm[both]+Enet[alpha=0.9] |
| SMARCA4 | Stepglm[both]+Enet[alpha=0.9] |
| CD3E | Stepglm[both]+Enet[alpha=0.9] |
| CD3G | Stepglm[both]+Enet[alpha=0.9] |
| B2M | Stepglm[backward]+Enet[alpha=0.9] |
| CD3D | Stepglm[backward]+Enet[alpha=0.9] |
| SYK | Stepglm[backward]+Enet[alpha=0.9] |
| POLB | Stepglm[backward]+Enet[alpha=0.9] |
| PARP1 | Stepglm[backward]+Enet[alpha=0.9] |
| CASP3 | Stepglm[backward]+Enet[alpha=0.9] |
| ZAP70 | Stepglm[backward]+Enet[alpha=0.9] |
| SMARCA4 | Stepglm[backward]+Enet[alpha=0.9] |
| CD3E | Stepglm[backward]+Enet[alpha=0.9] |
| CD3G | Stepglm[backward]+Enet[alpha=0.9] |
| B2M | Stepglm[both]+Enet[alpha=0.1] |
| CD3D | Stepglm[both]+Enet[alpha=0.1] |
| SYK | Stepglm[both]+Enet[alpha=0.1] |
| POLB | Stepglm[both]+Enet[alpha=0.1] |
| PARP1 | Stepglm[both]+Enet[alpha=0.1] |
| CASP3 | Stepglm[both]+Enet[alpha=0.1] |
| ZAP70 | Stepglm[both]+Enet[alpha=0.1] |
| SMARCA4 | Stepglm[both]+Enet[alpha=0.1] |
| CD3E | Stepglm[both]+Enet[alpha=0.1] |
| CD3G | Stepglm[both]+Enet[alpha=0.1] |
| B2M | Stepglm[backward]+Enet[alpha=0.1] |
| CD3D | Stepglm[backward]+Enet[alpha=0.1] |
| SYK | Stepglm[backward]+Enet[alpha=0.1] |
| POLB | Stepglm[backward]+Enet[alpha=0.1] |
| PARP1 | Stepglm[backward]+Enet[alpha=0.1] |
| CASP3 | Stepglm[backward]+Enet[alpha=0.1] |
| ZAP70 | Stepglm[backward]+Enet[alpha=0.1] |
| SMARCA4 | Stepglm[backward]+Enet[alpha=0.1] |
| CD3E | Stepglm[backward]+Enet[alpha=0.1] |
| CD3G | Stepglm[backward]+Enet[alpha=0.1] |
| B2M | Stepglm[both]+Enet[alpha=0.8] |
| CD3D | Stepglm[both]+Enet[alpha=0.8] |
| SYK | Stepglm[both]+Enet[alpha=0.8] |
| POLB | Stepglm[both]+Enet[alpha=0.8] |
| PARP1 | Stepglm[both]+Enet[alpha=0.8] |
| CASP3 | Stepglm[both]+Enet[alpha=0.8] |
| ZAP70 | Stepglm[both]+Enet[alpha=0.8] |
| SMARCA4 | Stepglm[both]+Enet[alpha=0.8] |
| CD3E | Stepglm[both]+Enet[alpha=0.8] |
| CD3G | Stepglm[both]+Enet[alpha=0.8] |
| B2M | Stepglm[backward]+Enet[alpha=0.8] |
| CD3D | Stepglm[backward]+Enet[alpha=0.8] |
| SYK | Stepglm[backward]+Enet[alpha=0.8] |
| POLB | Stepglm[backward]+Enet[alpha=0.8] |
| PARP1 | Stepglm[backward]+Enet[alpha=0.8] |
| CASP3 | Stepglm[backward]+Enet[alpha=0.8] |
| ZAP70 | Stepglm[backward]+Enet[alpha=0.8] |
| SMARCA4 | Stepglm[backward]+Enet[alpha=0.8] |
| CD3E | Stepglm[backward]+Enet[alpha=0.8] |
| CD3G | Stepglm[backward]+Enet[alpha=0.8] |
| B2M | Stepglm[both]+Enet[alpha=0.2] |
| CD3D | Stepglm[both]+Enet[alpha=0.2] |
| SYK | Stepglm[both]+Enet[alpha=0.2] |
| POLB | Stepglm[both]+Enet[alpha=0.2] |
| PARP1 | Stepglm[both]+Enet[alpha=0.2] |
| CASP3 | Stepglm[both]+Enet[alpha=0.2] |
| ZAP70 | Stepglm[both]+Enet[alpha=0.2] |
| SMARCA4 | Stepglm[both]+Enet[alpha=0.2] |
| CD3E | Stepglm[both]+Enet[alpha=0.2] |
| CD3G | Stepglm[both]+Enet[alpha=0.2] |
| B2M | Stepglm[backward]+Enet[alpha=0.2] |
| CD3D | Stepglm[backward]+Enet[alpha=0.2] |
| SYK | Stepglm[backward]+Enet[alpha=0.2] |
| POLB | Stepglm[backward]+Enet[alpha=0.2] |
| PARP1 | Stepglm[backward]+Enet[alpha=0.2] |
| CASP3 | Stepglm[backward]+Enet[alpha=0.2] |
| ZAP70 | Stepglm[backward]+Enet[alpha=0.2] |
| SMARCA4 | Stepglm[backward]+Enet[alpha=0.2] |
| CD3E | Stepglm[backward]+Enet[alpha=0.2] |
| CD3G | Stepglm[backward]+Enet[alpha=0.2] |
| B2M | Stepglm[both]+Lasso |
| CD3D | Stepglm[both]+Lasso |
| SYK | Stepglm[both]+Lasso |
| POLB | Stepglm[both]+Lasso |
| PARP1 | Stepglm[both]+Lasso |
| CASP3 | Stepglm[both]+Lasso |
| ZAP70 | Stepglm[both]+Lasso |
| SMARCA4 | Stepglm[both]+Lasso |
| CD3E | Stepglm[both]+Lasso |
| CD3G | Stepglm[both]+Lasso |
| B2M | Stepglm[backward]+Lasso |
| CD3D | Stepglm[backward]+Lasso |
| SYK | Stepglm[backward]+Lasso |
| POLB | Stepglm[backward]+Lasso |
| PARP1 | Stepglm[backward]+Lasso |
| CASP3 | Stepglm[backward]+Lasso |
| ZAP70 | Stepglm[backward]+Lasso |
| SMARCA4 | Stepglm[backward]+Lasso |
| CD3E | Stepglm[backward]+Lasso |
| CD3G | Stepglm[backward]+Lasso |
| B2M | Stepglm[both]+Enet[alpha=0.6] |
| CD3D | Stepglm[both]+Enet[alpha=0.6] |
| SYK | Stepglm[both]+Enet[alpha=0.6] |
| POLB | Stepglm[both]+Enet[alpha=0.6] |
| PARP1 | Stepglm[both]+Enet[alpha=0.6] |
| CASP3 | Stepglm[both]+Enet[alpha=0.6] |
| ZAP70 | Stepglm[both]+Enet[alpha=0.6] |
| SMARCA4 | Stepglm[both]+Enet[alpha=0.6] |
| CD3E | Stepglm[both]+Enet[alpha=0.6] |
| CD3G | Stepglm[both]+Enet[alpha=0.6] |
| B2M | Stepglm[backward]+Enet[alpha=0.6] |
| CD3D | Stepglm[backward]+Enet[alpha=0.6] |
| SYK | Stepglm[backward]+Enet[alpha=0.6] |
| POLB | Stepglm[backward]+Enet[alpha=0.6] |
| PARP1 | Stepglm[backward]+Enet[alpha=0.6] |
| CASP3 | Stepglm[backward]+Enet[alpha=0.6] |
| ZAP70 | Stepglm[backward]+Enet[alpha=0.6] |
| SMARCA4 | Stepglm[backward]+Enet[alpha=0.6] |
| CD3E | Stepglm[backward]+Enet[alpha=0.6] |
| CD3G | Stepglm[backward]+Enet[alpha=0.6] |
| ZAP70 | glmBoost+GBM |
| CASP3 | glmBoost+GBM |
| CASP9 | glmBoost+GBM |
| SMARCA4 | glmBoost+GBM |
| PARP1 | glmBoost+GBM |
| CD3G | glmBoost+GBM |
| SYK | glmBoost+GBM |
| POLB | glmBoost+GBM |
| B2M | glmBoost+GBM |
| B2M | Stepglm[both]+Enet[alpha=0.7] |
| CD3D | Stepglm[both]+Enet[alpha=0.7] |
| SYK | Stepglm[both]+Enet[alpha=0.7] |
| POLB | Stepglm[both]+Enet[alpha=0.7] |
| PARP1 | Stepglm[both]+Enet[alpha=0.7] |
| CASP3 | Stepglm[both]+Enet[alpha=0.7] |
| ZAP70 | Stepglm[both]+Enet[alpha=0.7] |
| SMARCA4 | Stepglm[both]+Enet[alpha=0.7] |
| CD3E | Stepglm[both]+Enet[alpha=0.7] |
| CD3G | Stepglm[both]+Enet[alpha=0.7] |
| B2M | Stepglm[backward]+Enet[alpha=0.7] |
| CD3D | Stepglm[backward]+Enet[alpha=0.7] |
| SYK | Stepglm[backward]+Enet[alpha=0.7] |
| POLB | Stepglm[backward]+Enet[alpha=0.7] |
| PARP1 | Stepglm[backward]+Enet[alpha=0.7] |
| CASP3 | Stepglm[backward]+Enet[alpha=0.7] |
| ZAP70 | Stepglm[backward]+Enet[alpha=0.7] |
| SMARCA4 | Stepglm[backward]+Enet[alpha=0.7] |
| CD3E | Stepglm[backward]+Enet[alpha=0.7] |
| CD3G | Stepglm[backward]+Enet[alpha=0.7] |
| B2M | Lasso+Stepglm[backward] |
| CD3D | Lasso+Stepglm[backward] |
| SYK | Lasso+Stepglm[backward] |
| POLB | Lasso+Stepglm[backward] |
| PARP1 | Lasso+Stepglm[backward] |
| CASP3 | Lasso+Stepglm[backward] |
| ZAP70 | Lasso+Stepglm[backward] |
| SMARCA4 | Lasso+Stepglm[backward] |
| CD3E | Lasso+Stepglm[backward] |
| CD3G | Lasso+Stepglm[backward] |
| B2M | Stepglm[both] |
| CD3D | Stepglm[both] |
| SYK | Stepglm[both] |
| POLB | Stepglm[both] |
| PARP1 | Stepglm[both] |
| CASP3 | Stepglm[both] |
| ZAP70 | Stepglm[both] |
| SMARCA4 | Stepglm[both] |
| CD3E | Stepglm[both] |
| CD3G | Stepglm[both] |
| B2M | Stepglm[backward] |
| CD3D | Stepglm[backward] |
| SYK | Stepglm[backward] |
| POLB | Stepglm[backward] |
| PARP1 | Stepglm[backward] |
| CASP3 | Stepglm[backward] |
| ZAP70 | Stepglm[backward] |
| SMARCA4 | Stepglm[backward] |
| CD3E | Stepglm[backward] |
| CD3G | Stepglm[backward] |
| B2M | glmBoost+Stepglm[both] |
| SYK | glmBoost+Stepglm[both] |
| POLB | glmBoost+Stepglm[both] |
| PARP1 | glmBoost+Stepglm[both] |
| CASP3 | glmBoost+Stepglm[both] |
| ZAP70 | glmBoost+Stepglm[both] |
| SMARCA4 | glmBoost+Stepglm[both] |
| B2M | glmBoost+Stepglm[backward] |
| SYK | glmBoost+Stepglm[backward] |
| POLB | glmBoost+Stepglm[backward] |
| PARP1 | glmBoost+Stepglm[backward] |
| CASP3 | glmBoost+Stepglm[backward] |
| ZAP70 | glmBoost+Stepglm[backward] |
| SMARCA4 | glmBoost+Stepglm[backward] |
| B2M | Stepglm[both]+Enet[alpha=0.4] |
| CD3D | Stepglm[both]+Enet[alpha=0.4] |
| SYK | Stepglm[both]+Enet[alpha=0.4] |
| POLB | Stepglm[both]+Enet[alpha=0.4] |
| PARP1 | Stepglm[both]+Enet[alpha=0.4] |
| CASP3 | Stepglm[both]+Enet[alpha=0.4] |
| ZAP70 | Stepglm[both]+Enet[alpha=0.4] |
| SMARCA4 | Stepglm[both]+Enet[alpha=0.4] |
| CD3E | Stepglm[both]+Enet[alpha=0.4] |
| CD3G | Stepglm[both]+Enet[alpha=0.4] |
| B2M | Stepglm[backward]+Enet[alpha=0.4] |
| CD3D | Stepglm[backward]+Enet[alpha=0.4] |
| SYK | Stepglm[backward]+Enet[alpha=0.4] |
| POLB | Stepglm[backward]+Enet[alpha=0.4] |
| PARP1 | Stepglm[backward]+Enet[alpha=0.4] |
| CASP3 | Stepglm[backward]+Enet[alpha=0.4] |
| ZAP70 | Stepglm[backward]+Enet[alpha=0.4] |
| SMARCA4 | Stepglm[backward]+Enet[alpha=0.4] |
| CD3E | Stepglm[backward]+Enet[alpha=0.4] |
| CD3G | Stepglm[backward]+Enet[alpha=0.4] |
| B2M | Stepglm[both]+Enet[alpha=0.3] |
| CD3D | Stepglm[both]+Enet[alpha=0.3] |
| SYK | Stepglm[both]+Enet[alpha=0.3] |
| POLB | Stepglm[both]+Enet[alpha=0.3] |
| PARP1 | Stepglm[both]+Enet[alpha=0.3] |
| CASP3 | Stepglm[both]+Enet[alpha=0.3] |
| ZAP70 | Stepglm[both]+Enet[alpha=0.3] |
| SMARCA4 | Stepglm[both]+Enet[alpha=0.3] |
| CD3E | Stepglm[both]+Enet[alpha=0.3] |
| CD3G | Stepglm[both]+Enet[alpha=0.3] |
| B2M | Stepglm[backward]+Enet[alpha=0.3] |
| CD3D | Stepglm[backward]+Enet[alpha=0.3] |
| SYK | Stepglm[backward]+Enet[alpha=0.3] |
| POLB | Stepglm[backward]+Enet[alpha=0.3] |
| PARP1 | Stepglm[backward]+Enet[alpha=0.3] |
| CASP3 | Stepglm[backward]+Enet[alpha=0.3] |
| ZAP70 | Stepglm[backward]+Enet[alpha=0.3] |
| SMARCA4 | Stepglm[backward]+Enet[alpha=0.3] |
| CD3E | Stepglm[backward]+Enet[alpha=0.3] |
| CD3G | Stepglm[backward]+Enet[alpha=0.3] |
| B2M | Stepglm[both]+glmBoost |
| SYK | Stepglm[both]+glmBoost |
| POLB | Stepglm[both]+glmBoost |
| PARP1 | Stepglm[both]+glmBoost |
| CASP3 | Stepglm[both]+glmBoost |
| ZAP70 | Stepglm[both]+glmBoost |
| SMARCA4 | Stepglm[both]+glmBoost |
| CD3G | Stepglm[both]+glmBoost |
| B2M | Stepglm[backward]+glmBoost |
| SYK | Stepglm[backward]+glmBoost |
| POLB | Stepglm[backward]+glmBoost |
| PARP1 | Stepglm[backward]+glmBoost |
| CASP3 | Stepglm[backward]+glmBoost |
| ZAP70 | Stepglm[backward]+glmBoost |
| SMARCA4 | Stepglm[backward]+glmBoost |
| CD3G | Stepglm[backward]+glmBoost |
| B2M | Stepglm[both]+Enet[alpha=0.5] |
| CD3D | Stepglm[both]+Enet[alpha=0.5] |
| SYK | Stepglm[both]+Enet[alpha=0.5] |
| POLB | Stepglm[both]+Enet[alpha=0.5] |
| PARP1 | Stepglm[both]+Enet[alpha=0.5] |
| CASP3 | Stepglm[both]+Enet[alpha=0.5] |
| ZAP70 | Stepglm[both]+Enet[alpha=0.5] |
| SMARCA4 | Stepglm[both]+Enet[alpha=0.5] |
| CD3E | Stepglm[both]+Enet[alpha=0.5] |
| CD3G | Stepglm[both]+Enet[alpha=0.5] |
| B2M | Stepglm[backward]+Enet[alpha=0.5] |
| CD3D | Stepglm[backward]+Enet[alpha=0.5] |
| SYK | Stepglm[backward]+Enet[alpha=0.5] |
| POLB | Stepglm[backward]+Enet[alpha=0.5] |
| PARP1 | Stepglm[backward]+Enet[alpha=0.5] |
| CASP3 | Stepglm[backward]+Enet[alpha=0.5] |
| ZAP70 | Stepglm[backward]+Enet[alpha=0.5] |
| SMARCA4 | Stepglm[backward]+Enet[alpha=0.5] |
| CD3E | Stepglm[backward]+Enet[alpha=0.5] |
| CD3G | Stepglm[backward]+Enet[alpha=0.5] |
| ZAP70 | Lasso+GBM |
| CASP3 | Lasso+GBM |
| CASP9 | Lasso+GBM |
| SMARCA4 | Lasso+GBM |
| CD247 | Lasso+GBM |
| PRKDC | Lasso+GBM |
| PARP1 | Lasso+GBM |
| SYK | Lasso+GBM |
| CD3G | Lasso+GBM |
| CD3E | Lasso+GBM |
| POLB | Lasso+GBM |
| B2M | Lasso+GBM |
| CD3D | Lasso+GBM |
| ZAP70 | GBM |
| CASP3 | GBM |
| CASP9 | GBM |
| SMARCA4 | GBM |
| CD247 | GBM |
| PRKDC | GBM |
| PARP1 | GBM |
| SYK | GBM |
| CD3G | GBM |
| CD3E | GBM |
| B2M | GBM |
| POLB | GBM |
| XRCC5 | GBM |
| XRCC6 | GBM |
| CD3D | GBM |
| CD8A | GBM |
| B2M | Stepglm[both]+SVM |
| CD3D | Stepglm[both]+SVM |
| SYK | Stepglm[both]+SVM |
| POLB | Stepglm[both]+SVM |
| PARP1 | Stepglm[both]+SVM |
| CASP3 | Stepglm[both]+SVM |
| ZAP70 | Stepglm[both]+SVM |
| SMARCA4 | Stepglm[both]+SVM |
| CD3E | Stepglm[both]+SVM |
| CD3G | Stepglm[both]+SVM |
| B2M | Stepglm[backward]+SVM |
| CD3D | Stepglm[backward]+SVM |
| SYK | Stepglm[backward]+SVM |
| POLB | Stepglm[backward]+SVM |
| PARP1 | Stepglm[backward]+SVM |
| CASP3 | Stepglm[backward]+SVM |
| ZAP70 | Stepglm[backward]+SVM |
| SMARCA4 | Stepglm[backward]+SVM |
| CD3E | Stepglm[backward]+SVM |
| CD3G | Stepglm[backward]+SVM |
| ZAP70 | Stepglm[both]+GBM |
| CASP3 | Stepglm[both]+GBM |
| SMARCA4 | Stepglm[both]+GBM |
| PARP1 | Stepglm[both]+GBM |
| POLB | Stepglm[both]+GBM |
| B2M | Stepglm[both]+GBM |
| CD3E | Stepglm[both]+GBM |
| CD3G | Stepglm[both]+GBM |
| SYK | Stepglm[both]+GBM |
| CD3D | Stepglm[both]+GBM |
| ZAP70 | Stepglm[backward]+GBM |
| CASP3 | Stepglm[backward]+GBM |
| SMARCA4 | Stepglm[backward]+GBM |
| B2M | Stepglm[backward]+GBM |
| POLB | Stepglm[backward]+GBM |
| PARP1 | Stepglm[backward]+GBM |
| CD3E | Stepglm[backward]+GBM |
| SYK | Stepglm[backward]+GBM |
| CD3G | Stepglm[backward]+GBM |
| CD3D | Stepglm[backward]+GBM |
| B2M | LDA |
| CD247 | LDA |
| CD3D | LDA |
| SYK | LDA |
| CD8A | LDA |
| POLB | LDA |
| XRCC6 | LDA |
| PARP1 | LDA |
| CASP3 | LDA |
| PRKDC | LDA |
| ZAP70 | LDA |
| SMARCA4 | LDA |
| XRCC5 | LDA |
| CD3E | LDA |
| CD3G | LDA |
| CASP9 | LDA |
| B2M | glmBoost+LDA |
| SYK | glmBoost+LDA |
| POLB | glmBoost+LDA |
| PARP1 | glmBoost+LDA |
| CASP3 | glmBoost+LDA |
| ZAP70 | glmBoost+LDA |
| SMARCA4 | glmBoost+LDA |
| CD3G | glmBoost+LDA |
| CASP9 | glmBoost+LDA |
| B2M | Stepglm[both]+LDA |
| CD3D | Stepglm[both]+LDA |
| SYK | Stepglm[both]+LDA |
| POLB | Stepglm[both]+LDA |
| PARP1 | Stepglm[both]+LDA |
| CASP3 | Stepglm[both]+LDA |
| ZAP70 | Stepglm[both]+LDA |
| SMARCA4 | Stepglm[both]+LDA |
| CD3E | Stepglm[both]+LDA |
| CD3G | Stepglm[both]+LDA |
| B2M | Stepglm[backward]+LDA |
| CD3D | Stepglm[backward]+LDA |
| SYK | Stepglm[backward]+LDA |
| POLB | Stepglm[backward]+LDA |
| PARP1 | Stepglm[backward]+LDA |
| CASP3 | Stepglm[backward]+LDA |
| ZAP70 | Stepglm[backward]+LDA |
| SMARCA4 | Stepglm[backward]+LDA |
| CD3E | Stepglm[backward]+LDA |
| CD3G | Stepglm[backward]+LDA |
| B2M | Lasso+LDA |
| CD247 | Lasso+LDA |
| CD3D | Lasso+LDA |
| SYK | Lasso+LDA |
| POLB | Lasso+LDA |
| PARP1 | Lasso+LDA |
| CASP3 | Lasso+LDA |
| PRKDC | Lasso+LDA |
| ZAP70 | Lasso+LDA |
| SMARCA4 | Lasso+LDA |
| CD3E | Lasso+LDA |
| CD3G | Lasso+LDA |
| CASP9 | Lasso+LDA |
| B2M | XGBoost |
| CD247 | XGBoost |
| CD3D | XGBoost |
| SYK | XGBoost |
| CD8A | XGBoost |
| POLB | XGBoost |
| XRCC6 | XGBoost |
| PARP1 | XGBoost |
| CASP3 | XGBoost |
| PRKDC | XGBoost |
| ZAP70 | XGBoost |
| SMARCA4 | XGBoost |
| XRCC5 | XGBoost |
| CD3E | XGBoost |
| CD3G | XGBoost |
| CASP9 | XGBoost |
| B2M | Lasso+XGBoost |
| CD247 | Lasso+XGBoost |
| CD3D | Lasso+XGBoost |
| SYK | Lasso+XGBoost |
| POLB | Lasso+XGBoost |
| PARP1 | Lasso+XGBoost |
| CASP3 | Lasso+XGBoost |
| PRKDC | Lasso+XGBoost |
| ZAP70 | Lasso+XGBoost |
| SMARCA4 | Lasso+XGBoost |
| CD3E | Lasso+XGBoost |
| CD3G | Lasso+XGBoost |
| CASP9 | Lasso+XGBoost |
| B2M | glmBoost+XGBoost |
| SYK | glmBoost+XGBoost |
| POLB | glmBoost+XGBoost |
| PARP1 | glmBoost+XGBoost |
| CASP3 | glmBoost+XGBoost |
| ZAP70 | glmBoost+XGBoost |
| SMARCA4 | glmBoost+XGBoost |
| CD3G | glmBoost+XGBoost |
| CASP9 | glmBoost+XGBoost |
| B2M | Stepglm[both]+XGBoost |
| CD3D | Stepglm[both]+XGBoost |
| SYK | Stepglm[both]+XGBoost |
| POLB | Stepglm[both]+XGBoost |
| PARP1 | Stepglm[both]+XGBoost |
| CASP3 | Stepglm[both]+XGBoost |
| ZAP70 | Stepglm[both]+XGBoost |
| SMARCA4 | Stepglm[both]+XGBoost |
| CD3E | Stepglm[both]+XGBoost |
| CD3G | Stepglm[both]+XGBoost |
| B2M | Stepglm[backward]+XGBoost |
| CD3D | Stepglm[backward]+XGBoost |
| SYK | Stepglm[backward]+XGBoost |
| POLB | Stepglm[backward]+XGBoost |
| PARP1 | Stepglm[backward]+XGBoost |
| CASP3 | Stepglm[backward]+XGBoost |
| ZAP70 | Stepglm[backward]+XGBoost |
| SMARCA4 | Stepglm[backward]+XGBoost |
| CD3E | Stepglm[backward]+XGBoost |
| CD3G | Stepglm[backward]+XGBoost |
| B2M | NaiveBayes |
| CD247 | NaiveBayes |
| CD3D | NaiveBayes |
| SYK | NaiveBayes |
| CD8A | NaiveBayes |
| POLB | NaiveBayes |
| XRCC6 | NaiveBayes |
| PARP1 | NaiveBayes |
| CASP3 | NaiveBayes |
| PRKDC | NaiveBayes |
| ZAP70 | NaiveBayes |
| SMARCA4 | NaiveBayes |
| XRCC5 | NaiveBayes |
| CD3E | NaiveBayes |
| CD3G | NaiveBayes |
| CASP9 | NaiveBayes |
| B2M | Lasso+NaiveBayes |
| CD247 | Lasso+NaiveBayes |
| CD3D | Lasso+NaiveBayes |
| SYK | Lasso+NaiveBayes |
| POLB | Lasso+NaiveBayes |
| PARP1 | Lasso+NaiveBayes |
| CASP3 | Lasso+NaiveBayes |
| PRKDC | Lasso+NaiveBayes |
| ZAP70 | Lasso+NaiveBayes |
| SMARCA4 | Lasso+NaiveBayes |
| CD3E | Lasso+NaiveBayes |
| CD3G | Lasso+NaiveBayes |
| CASP9 | Lasso+NaiveBayes |
| B2M | glmBoost+NaiveBayes |
| SYK | glmBoost+NaiveBayes |
| POLB | glmBoost+NaiveBayes |
| PARP1 | glmBoost+NaiveBayes |
| CASP3 | glmBoost+NaiveBayes |
| ZAP70 | glmBoost+NaiveBayes |
| SMARCA4 | glmBoost+NaiveBayes |
| CD3G | glmBoost+NaiveBayes |
| CASP9 | glmBoost+NaiveBayes |
| B2M | Stepglm[both]+NaiveBayes |
| CD3D | Stepglm[both]+NaiveBayes |
| SYK | Stepglm[both]+NaiveBayes |
| POLB | Stepglm[both]+NaiveBayes |
| PARP1 | Stepglm[both]+NaiveBayes |
| CASP3 | Stepglm[both]+NaiveBayes |
| ZAP70 | Stepglm[both]+NaiveBayes |
| SMARCA4 | Stepglm[both]+NaiveBayes |
| CD3E | Stepglm[both]+NaiveBayes |
| CD3G | Stepglm[both]+NaiveBayes |
| B2M | Stepglm[backward]+NaiveBayes |
| CD3D | Stepglm[backward]+NaiveBayes |
| SYK | Stepglm[backward]+NaiveBayes |
| POLB | Stepglm[backward]+NaiveBayes |
| PARP1 | Stepglm[backward]+NaiveBayes |
| CASP3 | Stepglm[backward]+NaiveBayes |
| ZAP70 | Stepglm[backward]+NaiveBayes |
| SMARCA4 | Stepglm[backward]+NaiveBayes |
| CD3E | Stepglm[backward]+NaiveBayes |
| CD3G | Stepglm[backward]+NaiveBayes |

**Table S9** | MR analyses between feature genes (eQTLs and pQTLs) and cardioembolic stroke.

|  | **Exposure** | **Outcome** | **MR Methods** | **N SNPs** | **OR (95%CI)** | ***P*-value** | **Heterogeneity** | **Horizontal Pleiotropy** |
| --- | --- | --- | --- | --- | --- | --- | --- | --- |
|  |  |  |  |  |  |  | **IVW *P*-value** | **MR Egger *P*-value** |
| eQTL | CASP3 | CES | IVW | 2 | 1.25 (1.01-1.56) | 0.037 | 0.41 | NA |
|  | CASP9 | CES | Wald ratio | 1 | 1.06 (0.81-1.38) | 0.688 | NA | NA |
|  | PARP1 | CES | Wald ratio | 1 | 0.93 (0.54-1.58) | 0.776 | NA | NA |
| pQTL | B2M | CES | IVW | 15 | 1.01 (0.97-1.05) | 0.762 | 0.53 | 0.996 |
|  | CASP3 | CES | IVW | 14 | 1.00 (0.96-1.05) | 0.856 | 0.22 | 0.990 |
|  | CASP9 | CES | IVW | 21 | 1.01 (0.98-1.04) | 0.480 | 0.57 | 0.946 |
|  | CD3D | CES | IVW | 14 | 1.03 (0.99-1.07) | 0.126 | 0.35 | 0.800 |
|  | CD3E | CES | IVW | 16 | 1.03 (0.99-1.08) | 0.161 | 0.16 | 0.818 |
|  | CD3G | CES | IVW | 9 | 1.05 (0.98-1.13) | 0.148 | 0.09 | 0.229 |
|  | CD8A | CES | IVW | 14 | 1.00 (0.97-1.03) | 0.947 | 0.84 | 0.322 |
|  | PARP1 | CES | IVW | 11 | 1.02 (0.96-1.08) | 0.590 | 0.11 | 0.544 |

**Table S10** | SMR analyses between feature genes and cardioembolic stroke.

| **Gene** | **b_SMR** | **se_SMR** | **p_SMR** |
| --- | --- | --- | --- |
| CASP9 | 0.0531671 | 0.132891 | 6.89E-01 |
| PARP1 | 0.26854 | 0.275985 | 3.31E-01 |
| CASP3 | 0.293232 | 0.14398 | 4.17E-02 |
| CD3E | -0.0447181 | 0.16137 | 7.82E-01 |
| B2M | 0.182043 | 0.331072 | 5.82E-01 |
| XRCC6 | 0.204482 | 0.350021 | 5.59E-01 |
